# MERTK-Dependent Ensheathment of Photoreceptor Outer Segments by Human Pluripotent Stem Cell-Derived Retinal Pigment Epithelium

**DOI:** 10.1016/j.stemcr.2020.02.004

**Published:** 2020-03-10

**Authors:** Seba Almedawar, Katerina Vafia, Sven Schreiter, Katrin Neumann, Shahryar Khattak, Thomas Kurth, Marius Ader, Mike O. Karl, Stephen H. Tsang, Elly M. Tanaka

**Affiliations:** 1 Technische Universität Dresden, Center for Molecular and Cellular Bioengineering (CMCB), Center for Regenerative Therapies Dresden (CRTD), Fetscherstraße 105, 01307 Dresden, Germany; 2 Technische Universität Dresden, Center for Molecular and Cellular Bioengineering (CMCB), Technology Platform, Fetscherstraße 105, 01307 Dresden, Germany; 3 German Center for Neurodegenerative Diseases (DZNE) Dresden, Tatzberg 41, 01307 Dresden, Germany; 4 CUMC/Edward S. Harkness Eye Institute, 635 West 165th Street, New York, NY 10032, USA; 5 Research Institute of Molecular Pathology (IMP), Vienna Biocenter (VBC), Campus Vienna Biocenter 1, 1030 Vienna, Austria

**Keywords:** retinal pigment epithelium, photoreceptor outer segments, human embryonic stem cells, human pluripotent stem cells, phagocytosis, ensheathment, MERTK, GAS6, MFGE8, PROS1

## Abstract

Maintenance of a healthy photoreceptor-retinal pigment epithelium (RPE) interface is essential for vision. At the center of this interface, apical membrane protrusions stemming from the RPE ensheath photoreceptor outer segments (POS), and are possibly involved in the recycling of POS through phagocytosis. The molecules that regulate POS ensheathment and its relationship to phagocytosis remain to be deciphered. By means of ultrastructural analysis, we revealed that Mer receptor tyrosine kinase (MERTK) ligands, GAS6 and PROS1, rather than αVβ5 integrin receptor ligands, triggered POS ensheathment by human embryonic stem cell (hESC)-derived RPE. Furthermore, we found that ensheathment is required for POS fragmentation before internalization. Consistently, POS ensheathment, fragmentation, and internalization were abolished in *MERTK* mutant RPE, and rescue of MERTK expression in retinitis pigmentosa (RP38) patient RPE counteracted these defects. Our results suggest that loss of ensheathment due to MERTK dysfunction might contribute to vision impairment in RP38 patients.

## Highlights

• POS are ensheathed *in vitro* by human embryonic stem cell-derived RPE
• POS ensheathment is upregulated by MERTK ligands: GAS6 and PROS1
• αVβ5 integrin receptor ligands do not stimulate POS ensheathment
• MERTK is essential for POS ensheathment and fragmentation before internalization

## Graphical Abstract

**Figure:**
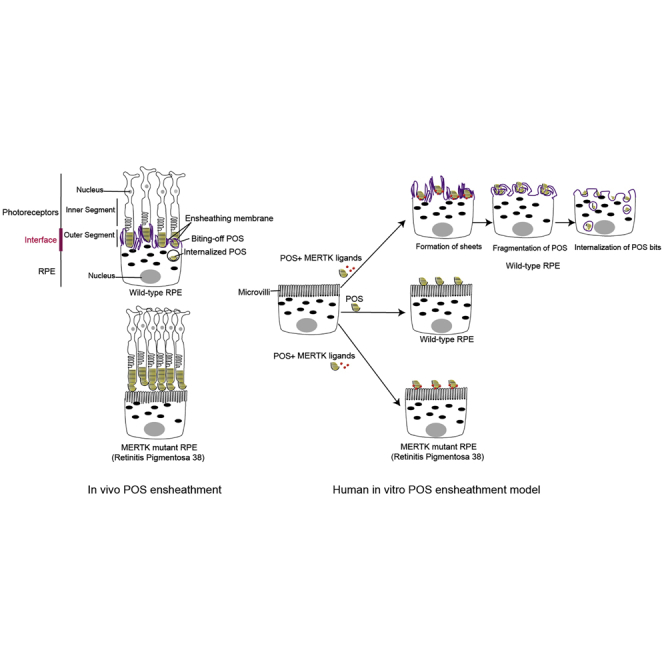

## Teaser

Using ultrastructural scanning electron microscopy (SEM)-based analysis, Almedawar et al. show that MERTK ligands, GAS6 and PROS1, upregulate photoreceptor outer segments (POS) ensheathment in human embryonic stem cell-derived retinal pigment epithelial cells. The authors also demonstrate that MERTK is essential for POS ensheathment and fragmentation before internalization, and suggest that ensheathment defects contribute to the pathology of retinitis pigmentosa type 38.

## Introduction

The retinal pigment epithelium (RPE) is a monolayer of pigmented epithelial cells that are essential for the function and survival of the overlying retinal photoreceptors and ultimately for the preservation of vision. During early human development, the RPE connects with the undifferentiated photoreceptors via adherens and gap junctions (Fisher and Lindberg, 1975). As soon as the photoreceptors differentiate and form outer segments, these junctions disappear and are replaced by sheets that interdigitate with the newly formed photoreceptor outer segments (POS) in the interphotoreceptor matrix (IPM). POS, found at the tips of photoreceptors, are formed from stacked membrane discs rich in opsin (rhodopsin [RHO] in the case of rods) and enclosed within the photoreceptor plasma membrane. RPE apical membrane protrusions rich in pigment granules extend in the form of “sheets” and wrap around POS in a process termed “POS ensheathment” (Steinberg et al., 1977). RPE ensheathing membranes contribute to the adhesion of the retina to the RPE (Wickham et al., 2012), and are at the site of phagocytosis of POS, which are detached daily from the overlying photoreceptors for recycling with the onset of light (Besharse et al., 1977) and under circadian control (LaVail, 1976). POS renewal and clearance are required to avoid accumulation of oxidized lipids and light-damaged proteins in the IPM and for the recycling of molecules involved in the light cycle (Strauss, 2005). However, it remains unclear whether POS are actively shed by photoreceptors, removed by extrinsic processes, or a combination of both. Of note, *in vivo* histology studies showed that ensheathing RPE membranes also invade POS structure, possibly during or before POS separation, suggesting that RPE might be “biting-off” POS tips, which could be part of the phagocytosis process (Steinberg et al., 1977). The study of POS ensheathment, separation, and phagocytosis has so far been limited by the low availability of primary human eyes, and thus hampered our understanding of the molecules that regulate ensheathment and its relationship to phagocytosis.

POS phagocytosis has been extensively studied *in vitro* and *in vivo* and key receptors and ligands that initiate signaling pathways, leading to phagocytosis, have been identified. Separation of POS leads to the curling of membrane discs (Steinberg et al., 1977) and exposes phosphatidylserines (PS), which are “eat me” signals that bind to CD36 receptors on the apical surface of RPE leading to activation of phagocytosis (Finnemann and Silverstein, 2001). Milk fat globule-EGF8 (MFGE8), secreted by the RPE, binds to exposed PS on POS, and serves as an “opsonin” that bridges POS to αVβ5 integrin receptor (Nandrot et al., 2007), which is found on the apical surface of the RPE. Binding of MFGE8-opsonized POS particles to αVβ5 integrin initiates two downstream signaling pathways. On the one hand, it stimulates Mer receptor tyrosine kinase (MERTK) via αVβ5 integrin-associated focal adhesion kinase (Nandrot et al., 2004). On the other hand, it activates RAC1-GTPase (Mao and Finnemann, 2012), which leads to F-actin recruitment to the phagocytic cup. Vitronectin (VTN) is another ligand for the αVβ5 integrin, but it does not seem to stimulate POS binding and internalization (Nandrot et al., 2007). MERTK is essential for POS phagocytosis (Feng et al., 2002, Karl et al., 2008). Two other ligands secreted by the RPE, namely growth arrest-specific protein 6 (GAS6) and protein S (PROS1), bind to PS on the surface of POS and to MERTK on the surface of RPE, and lead to MERTK phosphorylation and activation (Hall et al., 2001, Law et al., 2015). Phosphorylation of MERTK generates docking sites for Src homology 2 (SH2) proteins, such as phosphoinositide 3-kinases (Shelby et al., 2013), which is required for F-actin recruitment to the phagocytic cup (Bulloj et al., 2013). Previous studies have shown that ensheathing membranes are actin rich, and that blocking actin polymerization prevents POS separation and ensheathment (Matsumoto et al., 1987). Although downstream signaling of both αVβ5 integrin and MERTK leads to actin recruitment, their differential role during POS ensheathment is not known.

Mutations in RPE surface receptors result in phagocytosis defects in animal models of retinal degeneration and in humans. *Mertk* knockout rats (Bok and Hall, 1971) and mice (Duncan et al., 2002) lack a functional MERTK, which causes accumulation of POS in the IPM and subsequent photoreceptor degeneration. Similarly, mutations in *MERTK* lead to retinitis pigmentosa type 38 (RP38) in humans causing early onset retinal degeneration and blindness (Parinot and Nandrot, 2016). At present, the exact role of MERTK during POS phagocytosis, and the molecular and cellular events leading to loss of phagocytosis in its absence have not been fully deciphered.

POS ensheathment has been observed in human RPE explants (Steinberg et al., 1977), but to our knowledge it has not been explicitly studied in cultured human RPE. Human pluripotent stem cell (hPSC)-derived RPE have been shown useful for studying basic disease mechanisms (Ramsden et al., 2017).

In this study, we used hPSC-RPE to reconstitute and analyze homogeneously fragmented POS (F-POS) and whole full-sized POS (W-POS) ensheathment *in vitro* using SEM-based ultrastructural analysis. We observed that RPE cells extend membrane sheets that capture and ensheath F-POS *in vitro* within 3 h in a serum-dependent manner. Surprisingly, MERTK ligands, GAS6 and PROS1, rather than αVβ5 integrin receptor ligands, MFGE8 and VTN, stimulate ensheathment. We further showed that ensheathment participates in fragmenting W-POS before internalization. Consequently, RPE derived from human embryonic stem cells (hESCs), in which *MERTK* was knocked out using CRISPR/Cas9, or from an RP38 patient iPSC cell line with homozygous *MERTK* deletion, failed to ensheath, fragment, and internalize W-POS, which shows that MERTK is required for ensheathment and ensheathment-mediated fragmentation of POS, and implicates the loss of which in RP38 disease pathology and vision loss in patients.

## Results

### RPE Derived from Pluripotent Stem Cell Lines Display Mature RPE Characteristics

**Figure 1:**
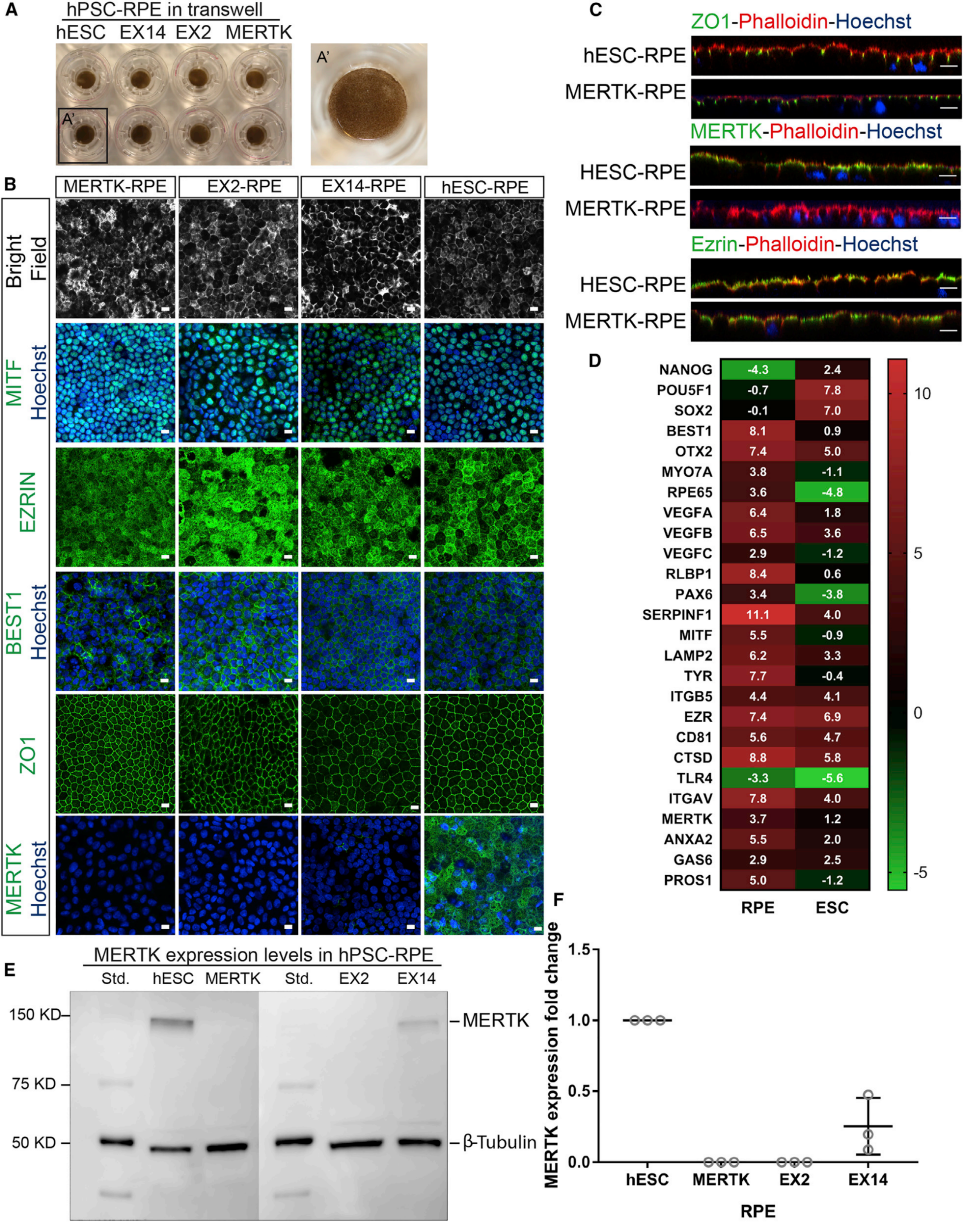
RPE Derived from Pluripotent Stem Cell Lines Display Mature RPE Characteristics (A) RPE derived from wild-type and *MERTK* mutant hPSC grown in Transwells. (B) HPSC-derived RPE are pigmented and express mature RPE markers, including MITF, EZRIN, bestrophin 1 (BEST1), and ZO1. MERTK is expressed in hESC-RPE, but not in EX2 and MERTK-RPE. It is weakly expressed in EX14-RPE. Scale bar, 10 μm. (C) Cross-sections of immunofluorescence images of RPE markers in hESC-RPE and MERTK-RPE. Note the polarized expression of ZO1, EZRIN, and MERTK on the apical surface of the RPE. Scale bar, 10 μm. (D) Heatmap of the mean of log2 values of reads per kilobase of transcript per million mapped reads of selected pluripotency genes and mature RPE markers. N = 3 biological repeats. Presented data are derived from RNA-seq analysis performed on hESCs and hESC-RPE. Please refer to Excel Table S1 for raw data. The accession number for the RNAseq data reported here is GEO: GSE127352. (E) Immunoblot (western blot) analysis of MERTK using total protein extracts from wild-type and *MERTK* mutant RPE. (F) Analysis of three MERTK immunoblots using ImageJ showed a decrease in MERTK expression in EX14-RPE, and no MERTK expression in EX2-RPE and MERTK-RPE. Data are represented as means ± SD. N = 3 biological repeats.

**Figure 2:**
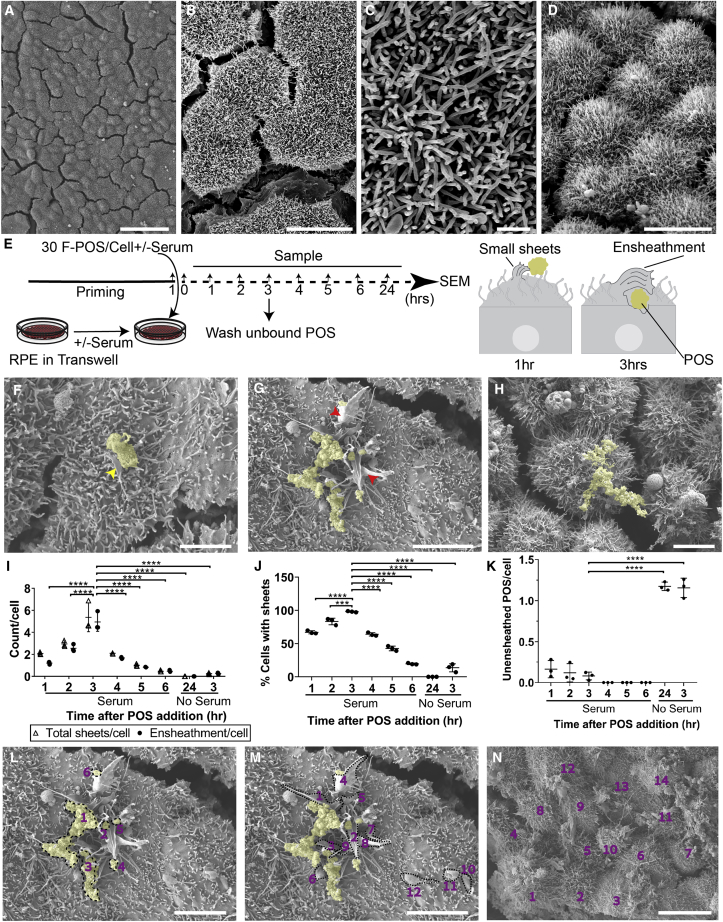
HESC-Derived RPE Recapitulate Dynamic POS Ensheathment *In Vitro* (A–D) SEM of hESC-RPE without POS. Scale bars, 50 μm (A), 10 μm (B), 1 μm (C), and 10 μm (D). (E) A schematic of the experiment. HESC-RPE cells were primed with 30% serum for 1 h before addition of F-POS or left untreated. After seeding F-POS, cells were incubated for 1, 2, or 3 h. After 3 h unbound POS were washed off with medium containing serum and cells were fixed at 4, 5, or 6 h. Samples without serum were incubated for 3 and 24 h before they were fixed and processed for SEM. (F–H) SEM images of hESC-RPE with F-POS. F-POS are artificially colored yellow. Scale bar, 5 μm (F). Cells that were treated with POS and serum for 1 h show smaller and fewer sheets. Yellow arrow points to small sheets with POS. (G) Cells that were treated with POS and serum for 3 h show larger and numerous sheets. Red arrow points to the big sheets surrounding POS. See also Figures S1 and S2. (H) Cells that were treated with POS without serum for 3 h did not form sheets. (I–K) Ten images containing around ten cells from each condition were analyzed. (I) The presence of serum and POS increases the number of total sheets per cell, and the number of sheets associated with POS. In the absence of serum very few sheets are observed. The number of sheets/cell is highest at 3 h after POS addition (pulse) and significantly decreases 3 h after POS pulse. Significance was calculated using two-way ANOVA comparing all samples to 3 h. (J) The percentage of cells that extend sheets in response to POS and serum increases significantly at 3 h and decreases again after 6 h. In contrast, cells that received POS without serum do not show ensheathment either at 3 h or at 24 h. (K) In the absence of serum most of the bound POS are unensheathed. Statistical significance for (J and K) was calculated using one-way ANOVA comparing all samples to 3 h. Data are represented as means ± SD. N = 3 biological repeats. ns > 0.05, ^∗^p < 0.05, ^∗∗^p < 0.01, ^∗∗∗^p < 0.001, ^∗∗∗∗^p < 0.0001. (L–N) Examples for quantifying F-POS, sheets, and cells within a field of view. (L) Manual segmentation of F-POS. Six POS fragments were counted. Scale bar, 5 μm. (M) Manual segmentation of sheets. Twelve sheets were counted. Scale bar, 5 μm. (N) Manual quantification of the cells. Fourteen cells were counted. Scale bar, 10 μm.

All RPE differentiated from wild-type hESCs, and *MERTK* mutant hESC and hiPSC lines, were highly pigmented (Figure 1A) and expressed mature RPE markers in a polarized fashion, including ZO1, bestrophin 1 (BEST1), MITF, and EZRIN (Figures 1B and 1C). RNA sequencing analysis of hESC-derived RPE (hESC-RPE) compared with hESCs also showed upregulation of mature RPE markers, including phagocytosis-related genes, and downregulation of pluripotency markers (Figure 1D). *MERTK* mutant RPE showed either no expression or very weak expression of MERTK as shown by immunostaining (Figure 1B) and immunoblot analysis (Figures 1E and 1F). Ultrastructural SEM analysis of hESC-RPE showed that cells have a defined hexagonal shape and long microvilli on their apical surface (Figures 2A–2D).

### HESC-Derived RPE Recapitulate Dynamic POS Ensheathment *In Vitro*

To characterize POS ensheathment in the RPE we fragmented our POS preparation before addition to the cells to obtain homogeneously sized POS (F-POS) and expose the inner membrane containing eat me signals required for phagocytosis (Figure S1A). In addition, to prepare the RPE cells for the incoming POS, we primed RPE for 1 h with serum, as it is known to induce phagocytosis (Karl et al., 2008). One hour after treatment with F-POS and 30% serum, the membrane of the RPE started to grow around POS (Figures 2E and 2F) and formed sheets that wrapped around them by 3 h (Figures 2G and S1B–S1G). In contrast, RPE treated with POS alone showed no sheet formation even after 24 h (Figure 2H). Treatment with serum in the absence of POS did not result in sheet formation either (N = 3). Quantification of RPE membrane sheets with and without associated POS (Figure 2I) showed that the percentage of cells harboring ensheathing membranes increased with time to reach around 90% after 3 h in the presence of serum, while in its absence the percentage of cells presenting sheets was negligible (Figure 2J). Consistently, the absence of serum led to an increase in the amount of POS that were not ensheathed by the RPE (unensheathed POS) (Figure 2K). Notably, the number of ensheathed POS in the presence of serum and the percentage of cells harboring ensheathing membranes following a 3-h pulse decreased gradually to reach minor levels after 6 h, indicating the dynamic nature of the formed sheets. Therefore, POS ensheathment by human RPE can be recapitulated *in vitro* in the presence of serum, which stimulates their formation.

In the mammalian retina, rods are more abundant than cones and are the first to degenerate in MERTK-related retinitis pigmentosa (RP38). The percentage of RHO-positive F-POS (rods outer segment marker) employed in all experiments was around 83% (Figure S2A) and membrane discs were visualized by transmission electron microscopy (TEM) (Figure S2B). RHO-positive F-POS were also seen interacting with membrane sheets by means of correlative fluorescence and SEM (Figures S2C–S2F), and by immunogold labeling in TEM (Figure S2G).

### Ensheathment by Human RPE Is Specific for POS

**Figure 3:**
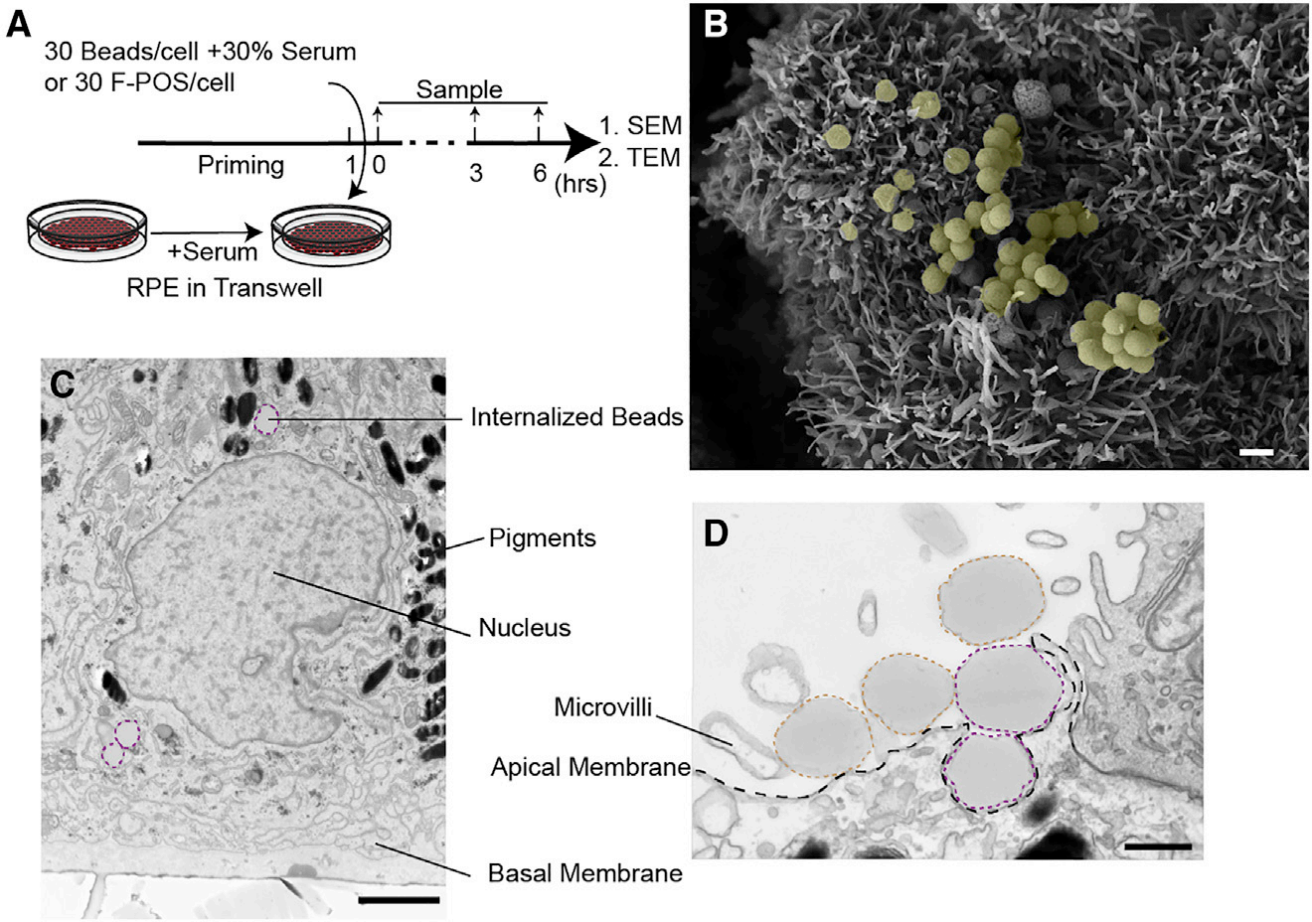
Ensheathment by Human RPE Is Specific for POS (A) A schematic of the experiment. HESC-RPE cells were primed with 30% serum for 1 h before addition of F-POS or latex beads. Control cells were either left untreated or treated with beads without serum. Cells were incubated for 3 h at 37°C. All samples were washed after 3 h. Some samples were fixed and others were kept for 3 h or more to allow internalization of beads. Samples were analyzed with SEM or TEM. (B) SEM image of RPE cells treated with beads (artificially colored yellow) for 3 h. Scale bar, 1 μm. (C) TEM image at 6 h. Beads (dashed purple line) were observed inside the cells and close to the nuclei. (D) TEM image at 3 h. Beads were observed either at the surface of the cell (dashed orange line), or getting internalized by the RPE (dashed purple line). In both SEM and TEM analyses, no sheets were observed around the beads.

To further validate the established hESC-RPE model, the specificity of membrane sheet formation with regard to F-POS was compared with that for latex beads, which are usually internalized by the RPE through non-specific endocytosis and independently of the presence of serum (Carr et al., 2009). We reasoned that if ensheathment is specific to POS, then none should be observed when beads are added in the presence of serum. As predicted, no sheets were observed at the point of interaction between RPE and beads (Figure 3B). Interestingly, despite the absence of sheet formation, these beads were internalized by the RPE, as seen by TEM analysis (Figures 3C and 3D), suggesting that the unspecific endocytosis pathway does not require ensheathment, and that ensheathment is specific for POS.

### MERTK Ligands Rather Than αVβ5 Integrin Ligands Stimulate POS Ensheathment by Human RPE

MFGE8 and GAS6 are known to stimulate phagocytosis in other RPE models through binding to their canonical receptors MERTK and αVβ5 integrin, respectively. To validate whether F-POS phagocytosis in hESC-RPE can be induced by serum or known phagocytosis ligands, such as MFGE8 and GAS6, different concentrations of both ligands were compared with serum (Figure S3A). GAS6 and serum stimulated POS internalization in a dose-dependent manner. MFGE8 stimulated both POS binding and internalization at higher concentrations. Serum showed higher POS internalization values than MFGE8 and GAS6 separately. However, combining MFGE8 and GAS6 upregulated internalization similarly to serum (Figures S3B–S3D). Taken together, these results show that POS phagocytosis can be induced by serum, and more specifically by GAS6 and MFGE8, confirming previously known roles of these ligands during phagocytosis in other RPE models.

**Figure 4:**
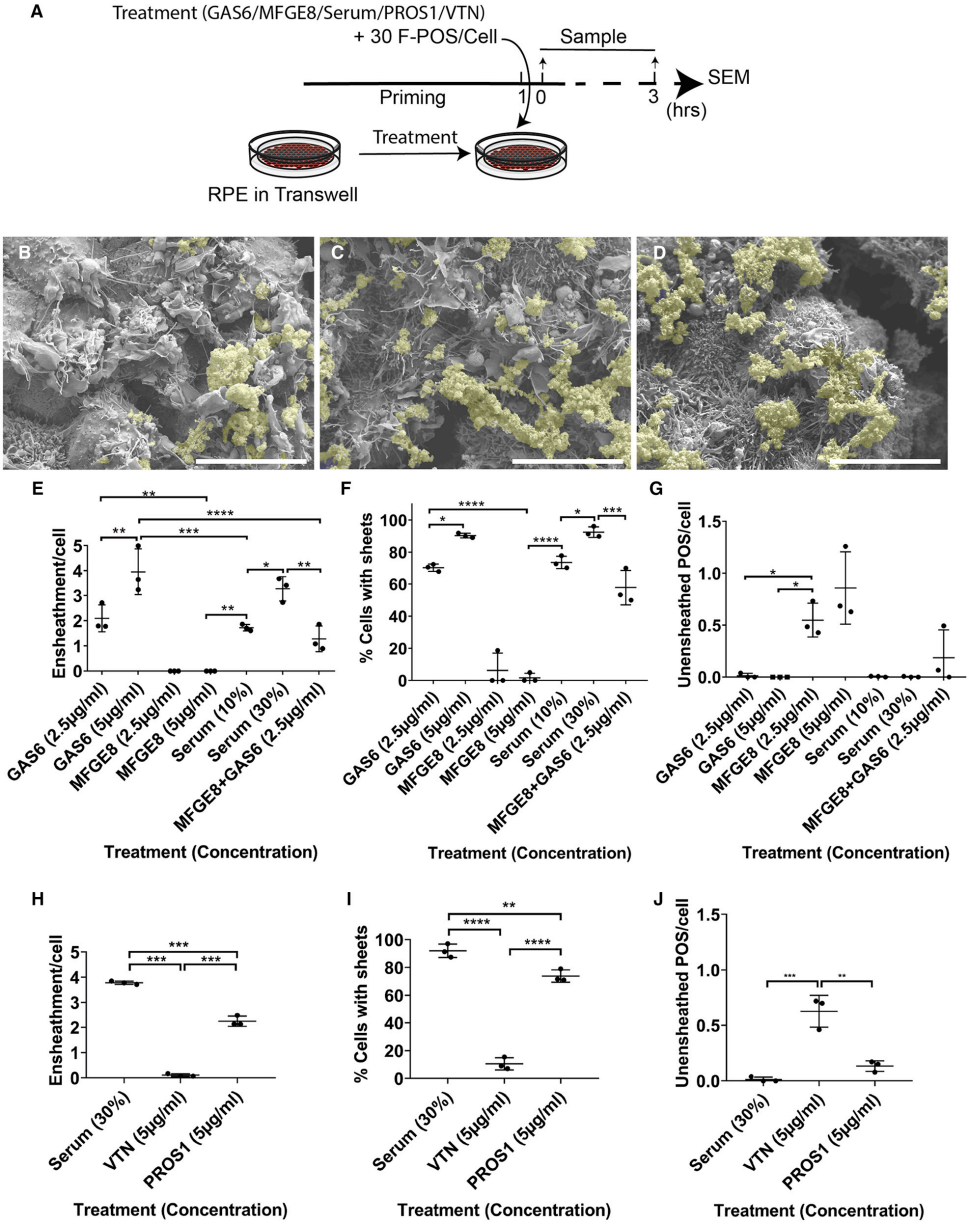
MERTK Ligands Rather Than αVβ5 Integrin Ligands Stimulate POS Ensheathment by Human RPE (A) A schematic of the experiment. (B–D) SEM image of cells treated with F-POS and 30% serum (B), 5 μg/mL GAS6 (C), or 5 μg/mL MFGE8 (D). Scale bar, 1 μm. (E–J) Ten images containing around ten cells from each condition were analyzed. Data are represented as means ± SD. N = 3 biological repeats. (E–J) Quantification of the number of sheets associated with POS per cell, the percentage of cells presenting sheets, and the number of unensheathed POS per cell in the different conditions. Significance was calculated using one-way ANOVA test. ns > 0.05, ^∗^p < 0.05, ^∗∗^ p< 0.01, ^∗∗∗^p < 0.001, ^∗∗∗∗^p < 0.0001. Data are represented as means ± SD. N = 3 biological repeats. Related to Figure S3.

To determine whether GAS6 and MFGE8 are also involved in triggering F-POS ensheathment similar to phagocytosis, increasing concentrations of GAS6, MFGE8, or serum were added to RPE (Figure 4A). Similar to serum, GAS6 increased POS ensheathment (Figures 4B, 4C, 4E, and 4F). GAS6 is known to bind to and activate MERTK, which suggests that ensheathment, which is stimulated by GAS6 addition, might be mediated through MERTK, and that other ligands that activate MERTK might have the same effect. For this purpose we tested whether PROS1, which is another known ligand for MERTK, might similarly stimulate ensheathment. As expected, PROS1 stimulated POS ensheathment significantly (Figures 4H–4J). In contrast, addition of MFGE8 did not induce POS ensheathment and led to accumulation of unensheathed POS at the apical surface of the RPE (Figures 4D–4G). Furthermore, there was no additive effect on ensheathment when both ligands were added in comparison with GAS6 alone (Figures 4E and 4F). Because MFGE8 is known to bind and activate αVβ5 integrin receptor, we evaluated whether VTN, another known αVβ5 integrin receptor ligand, can stimulate ensheathment. Similar to MFGE8, VTN showed very low levels of POS ensheathment, and an increase in the amount of unensheathed POS compared with serum (Figures 4H–4J). Thus, these results suggest that MERTK ligands are sufficient to trigger POS ensheathment, contrary to αVβ5 Integrin receptor ligands.

### *MERTK* Mutations in Human RPE Lead to Defects in POS Phagocytosis

**Figure 5:**
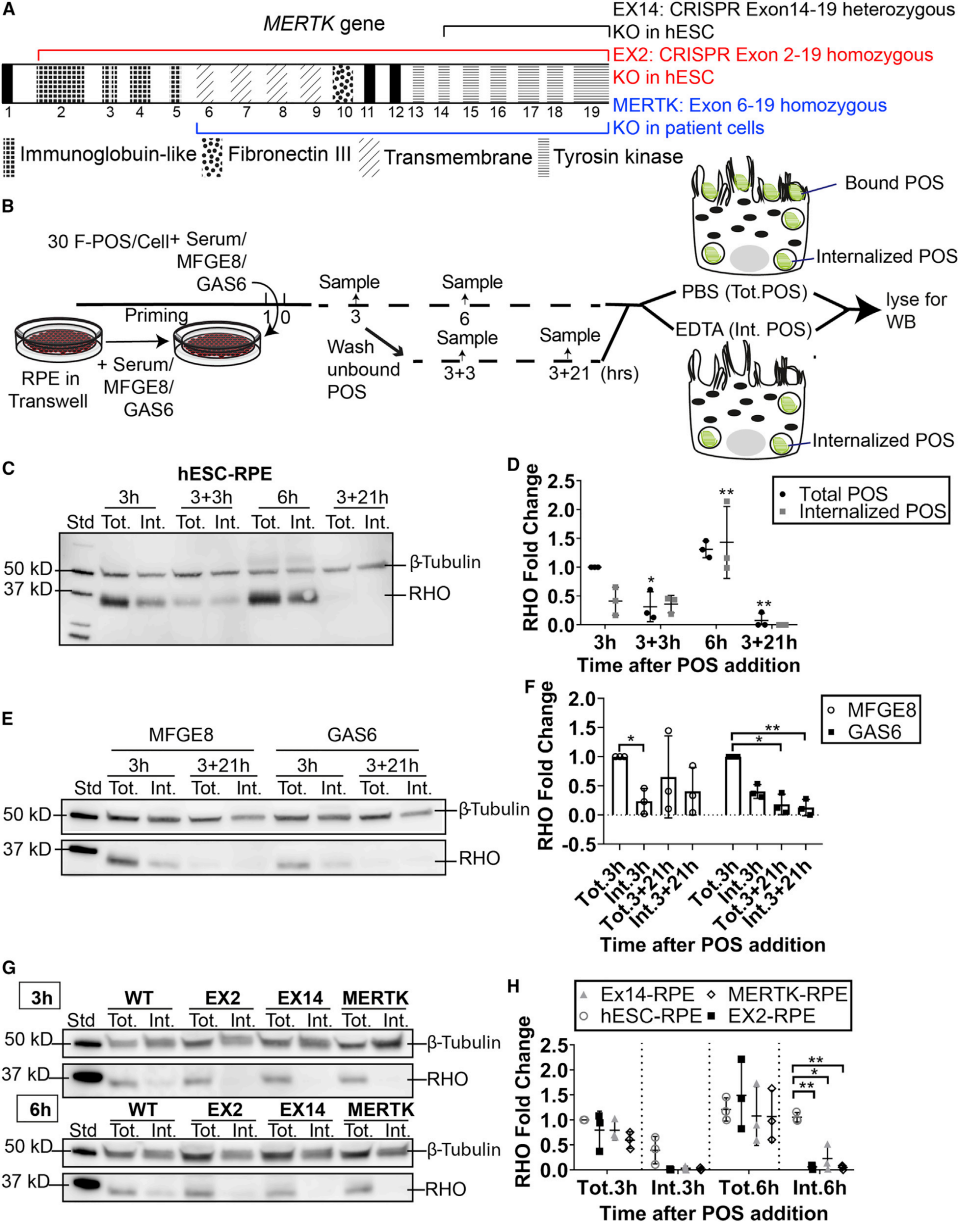
Mutations in the *MERTK* Gene Lead to Defects in POS Phagocytosis (A) An illustration of the *MERTK* gene showing the different exons coding for the different protein domains of the receptor. Exons are labeled 1 to 19. The mutations in the three stem cell lines used in this study are also represented in the illustration: the iPSC cell line derived from an RP38 patient has a homozygous deletion spanning exon 6 to 19 (blue). CRISPR/Cas9-mediated partial deletion of exon 14 to 19 in hESCs is shown in black. CRISPR/Cas9-mediated deletion of exon 2 to 19 in hESCs is shown in red. See also Figures S4–S7 for *MERTK* mutations details, patient clinical observations, stem cell characterization, and rescue in isogenic iPSC-RPE control. (B) A schematic of the three experiments in (C–H). All samples were treated with either PBS or EDTA before lysis to detect total versus internalized F-POS, respectively. Blots were probed for β-tubulin as a loading control, and RHO to indicate total POS (Tot.) and internalized POS (Int.). RHO signal was normalized to tubulin and then to the total POS signal in control condition to monitor the fold change in RHO signal. Data are represented as means ± SD. N = 3 biological repeats. Significance was calculated using two-way ANOVA test. ns > 0.05, ^∗^p < 0.05, ^∗∗^p < 0.01, ^∗∗∗^p < 0.001, ^∗∗∗∗^p < 0.0001. (C) HESC-RPE were primed for 1 h with 30% serum. Next, cells were challenged with F-POS and incubated for 3 or 6 h at 37°C. Samples were lysed 3 or 6 h after POS addition. Other samples were washed at 3 h and kept for 3 h (3 + 3 h) or 21 h (3 + 21 h) before lysis to monitor POS degradation. (D) Quantification of the immunoblot in (C). Total POS 3 h was used as control condition for normalization and statistical significance calculation. (E) HESC-RPE were primed for 1 h with either GAS6 or MFGE8. Next, cells were challenged with POS and incubated for 3 h and either lysed or washed and kept for a further 21 h (3 + 21 h) before lysis to monitor POS degradation. (F) Quantification of the immunoblot in (E). Total POS 3 h in each condition was used as control for normalization and statistical significance calculation. HESC-RPE bind, internalize, and degrade POS efficiently in the presence of GAS6 and serum. In contrast, in the presence of MFGE8 POS degradation is slowed down. See Figure S3 for fluorescence-based POS phagocytosis data. (G) Wild-type and *MERTK* mutant RPE were primed for 1 h with 30% serum. Next, cells were challenged with POS and incubated for 3 or 6 h at 37°C before processing. (H) Quantification of the immunoblot in (G). Total POS 3 h in hESC-RPE was used as control condition for normalization. For significance calculation all samples were compared with each other. *MERTK* mutant RPE show POS internalization defect.

The fact that GAS6 or PROS1 alone are sufficient to stimulate POS ensheathment suggested that their receptor, MERTK, is also involved in this process. Therefore, we hypothesized that the absence of *MERTK* might lead to defects in POS ensheathment. Two *MERTK* knockout hESC lines, namely EX2 (homozygous deletion from exon 2 onward) and EX14 (heterozygous deletion of exon 14 onward), were generated using CRISPR/Cas9 in hESCs. In addition, an iPSC line was derived from an RP38 patient harboring a genomic deletion in *MERTK* (Figures 5A and S4–S6)*. MERTK* mutations are known to cause an RPE phagocytosis defect in animal models, leading to accumulation of POS in the IPM, and retinal degeneration and vision loss in RP 38 patients (Duncan et al., 2002). To validate this phenotype within our human RPE model, we analyzed F-POS binding and internalization by means of immunoblot in healthy versus *MERTK* mutant RPE. At first, we determined the kinetics of POS binding, internalization, and degradation in wild-type hESC-RPE in the presence of 30% serum, under ensheathment conditions (Figures 5C and 5D). At 3 h after F-POS addition, most of the POS were bound and partially internalized. When cells were challenged with POS for 6 h, more POS were bound and internalized. In addition, when unbound POS were washed off at 3 h, all the bound POS were internalized and partially degraded after 6 h, and fully degraded after 24 h. Internalization of POS by wild-type hESC-RPE was observed in the presence and absence of ensheathment, when treated with GAS6 or MFGE8, respectively (Figures 5E and 5F). RHO signal significantly decreased after 24 h compared with 3 h in GAS6-treated cells indicating efficient POS degradation in the presence of ensheathment, while the drop in RHO signal seen in MFGE8-treated cells at the same time point was not significant and showed a lot of variation. Thus, under ensheathment conditions wild-type hESC-RPE were able to bind, internalize and degrade POS. In contrast, all *MERTK* mutant RPE cells had minimal if any internalization at 3 or 6 h after POS addition (Figures 5G and 5H). By means of CRISPR/Cas9-mediated genomic engineering, we integrated wild-type *MERTK* sequence in the genomic *MERTK* locus in the patient iPSC line (Figure S7A). Recovery of MERTK expression in patient MERTK-RPE (Figures S7B–S7D) resulted in correction of the phagocytosis defect (Figures S7E and S7F). This confirms that the phagocytosis defect seen in patient MERTK-RPE is due to the identified genomic deletion in *MERTK*.

### *MERTK* Mutations in Human RPE Abolish POS Ensheathment

**Figure 6:**
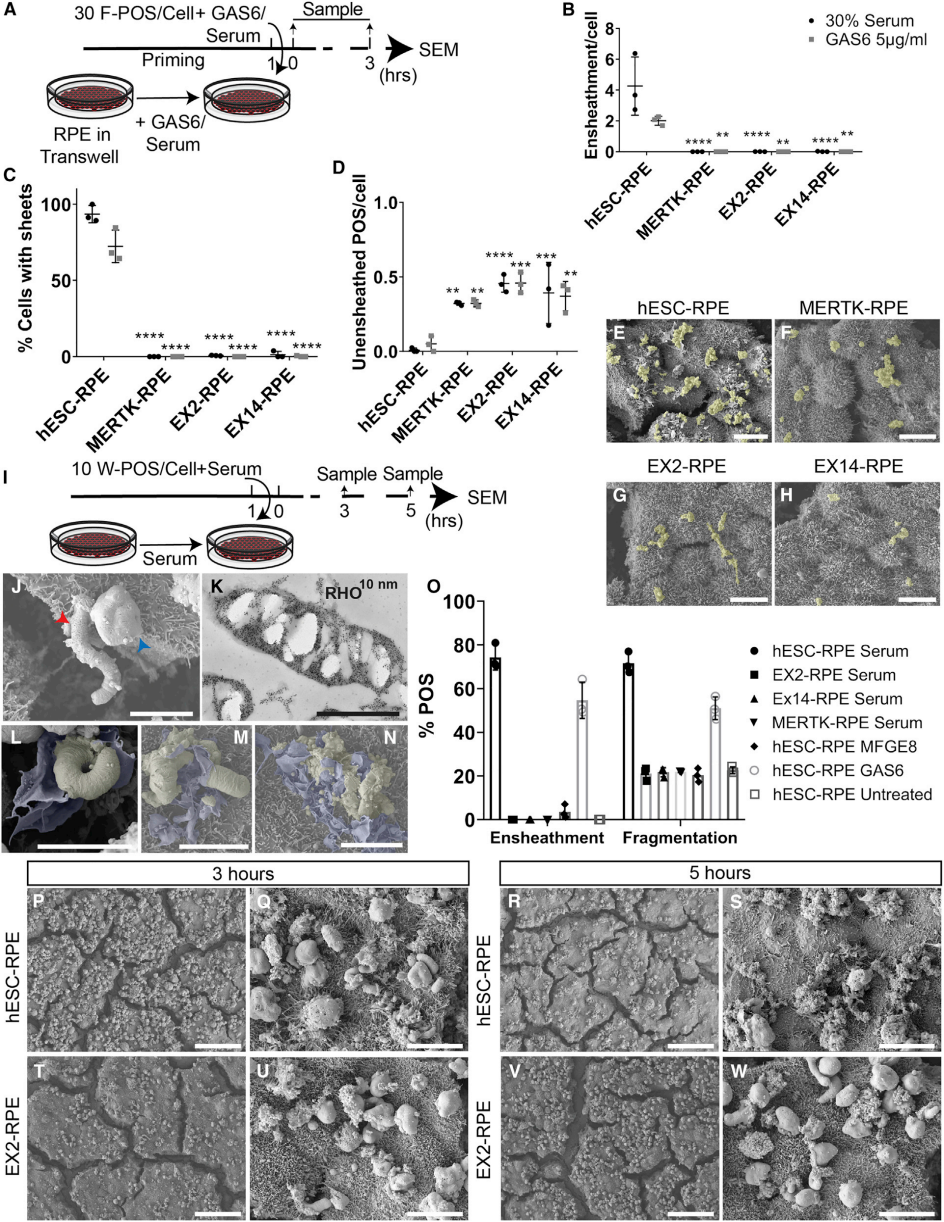
*MERTK* Mutations in Human RPE Abolish POS Ensheathment (A) A schematic of the experiment in (B–H). (B–D) Ten images containing around ten cells from each condition were analyzed. Significance was calculated using one-way ANOVA test. ns > 0.05, ^∗^p < 0.05, ^∗∗^p < 0.01, ^∗∗∗^p < 0.001, ^∗∗∗∗^p < 0.0001. Data are represented as means ± SD. N = 3 biological repeats. In Figure S7 ensheathment rescue in isogenic control is shown. (B) Quantification of the number of sheets associated with POS per cell. (C) Quantification of the percentage of cells presenting sheets. (D) Quantification of the number of unensheathed F-POS per cell. (E–H) SEM images of wild-type and *MERTK* mutant RPE treated with F-POS and 30% serum. Scale bar, 10 μm. (E) Wild-type hESC-RPE. (F) *MERTK* mutant MERTK-RPE. (G) *MERTK* mutant EX2-RPE. (H) *MERTK* mutant EX14-RPE. (I) A schematic of the experiment in (J–W). Wild-type and *MERTK* mutant RPE (grown on transwells) were primed 1 h with 30% serum. Alternatively, wild-type RPE cells were primed with 5 μg/mL GAS6 or MFGE8 for 1 h. Next, cells were challenged with W-POS and incubated for 3 and 5 h at 37°C. Samples were next processed for SEM. (J) Two types of W-POS particles were observed by SEM elongated (red arrow) and round-oval (blue arrow) outer segments. Scale bar, 5 μm. (K) Whole POS particles are RHO rich. Scale bar, 3 μm. (L–N) SEM images. Stages of POS binding, ensheathment, and ensheathment-mediated fragmentation. Scale bar, 5 μm. (L) Upon contact between hESC-RPE and W-POS in the presence of 30% serum, RPE extends its membrane sheets around the particle. (M) Next, RPE sheets invade POS and fragment them. (N) POS are completely fragmented before they are internalized. (O) Analysis of W-POS ensheathment in SEM images at 3 h and fragmentation by 5 h in the presence of 30% serum. Data are represented as means ± SD. N = 3 biological repeats. (P–W) Representative SEM images of the analysis shown in (O). Scale bars, 50 μm (P, R, T, and V). Zoomed-in SEM images of (Q), (S), (U), and (W). Scale bar, 10 μm. W-POS were ensheathed at 3 h (P and Q) and fragmented after 5 h by hESC-RPE treated with 30% serum (R and S), while EX2-RPE showed neither POS ensheathment at 3 h (T and U), nor ensheathment-mediated fragmentation at 5 h (V and W).

To determine the role of MERTK during POS ensheathment, ultrastructural SEM analysis of F-POS ensheathment was evaluated in wild-type versus *MERTK* mutant RPE in the presence of 30% serum or 5 μg/mL GAS6 (Figure 6A). In the absence of functional MERTK, F-POS bound to RPE microvilli and were not ensheathed by the RPE (Figures 6B–6H). Taken together, the human RPE model shows that MERTK is not only critical for POS phagocytosis, but also for membrane remodeling occurring at the RPE-photoreceptor interface leading to ensheathment.

### Ensheathment Is Required for POS Fragmentation before Internalization

Upon treatment of wild-type hESC-RPE with MFGE8, uptake of already F-POS can occur in the absence of ensheathment. Thus, we hypothesized that ensheathment might be required to fragment POS before internalization. For this reason, we modified our POS isolation method to obtain whole full-sized unfragmented POS (W-POS) from photoreceptor cells (Figure S1A). Of note, two types of POS could be distinguished by SEM: round-oval and elongated (Figure 6J), which were RHO rich as seen by TEM (Figure 6K). By means of SEM analysis, we observed that upon addition of W-POS to wild-type hESC-RPE, in the presence of 30% serum, ensheathing membranes established contact with W-POS (Figure 6L), invaded the outer membrane enclosing the discs (Figure 6M) and finally fragmented it (Figure 6N). Next, we explored the dynamics of W-POS ensheathment and its fragmentation in wild-type hESC-RPE treated with 5 μg/mL MFGE8, 5 μg/mL GAS6, or 30% serum; and in *MERTK* mutant RPE, treated with 30% serum. Analysis of SEM images (Figure 6O) showed that around 80% of W-POS, in hESC-RPE treated with serum, and 60% of W-POS, in hESC-RPE treated with GAS6, were ensheathed by 3 h (Figures 6P and 6Q), and fragmented by RPE ensheathing membranes by 5 h after addition (Figures 6R and 6S). However, around 20% of the POS in *MERTK* mutant RPE and MFGE8-treated wild-type RPE, appeared fragmented without associated ensheathment at both 3 and 5 h, and displayed neither ensheathment at 3 h (Figures 6T and 6U), nor ensheathment-associated fragmentation by 5 h (Figures 6V and 6W). These results show that ensheathment is associated with POS fragmentation in human RPE, and that POS ensheathment and ensheathment-mediated fragmentation is defective in the absence of a functional MERTK.

Recovery of MERTK expression in patient MERTK-RPE (Figures S7A–S7D) rescued the pathologic phenotype and resulted in the formation of proper ensheathing membranes and subsequently fragmentation of W-POS (Figures S7G–S7K). This confirms that the ensheathment defect seen in patient MERTK-RPE is due to the identified genomic deletion in *MERTK*.

**Figure 7:**
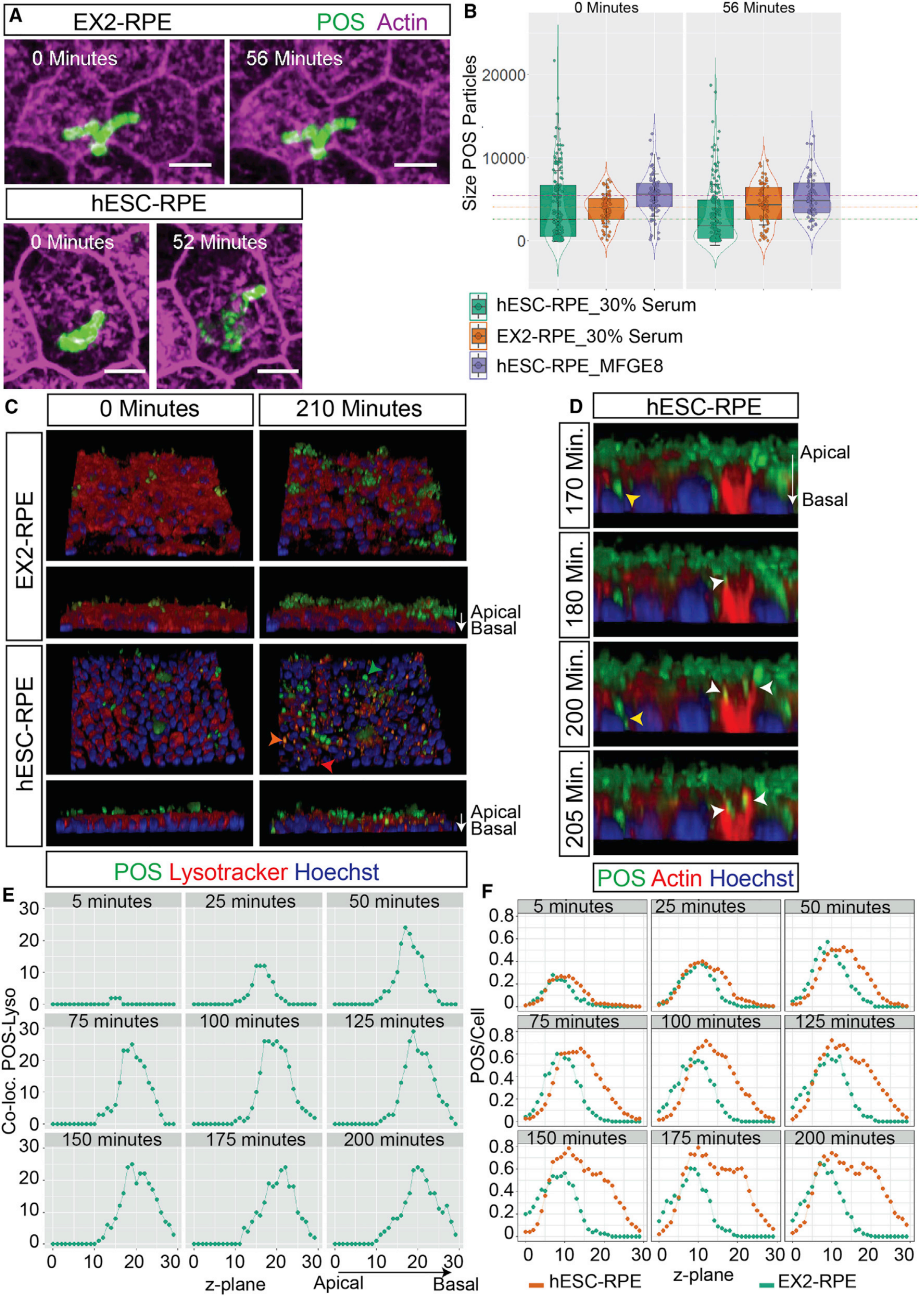
Quantitative Live Imaging Analysis Shows that *MERTK* Mutant Human RPE Fail to Fragment and Internalize POS (A) Snapshots from time-lapse live fluorescence imaging performed with LSM 880 upright microscope using the Airyscan feature and a 40× dipping objective. The complete time-lapse videos can be found in Videos S1a and S1b. HESC-RPE and EX2-RPE (grown on transwells) were primed with 30% serum or 5 μg/mL MFGE8 for 1 h, and were challenged with AF488-labeled W-POS for 2.5 h at 37°C with 5% CO_2_ before imaging. Membranes, containing the RPE monolayer, were cut out from the Transwell insert and placed with the apical side of the cells facing the dipping objective in a 35-mm cell culture dish with 2 mL medium containing SiR-actin dye and 30% serum. (A) Many of the whole POS particles that were still seen at the start of imaging (0 min) in hESC-RPE got fragmented within around 52 min. In contrast, whole POS particles in EX2-RPE did not get fragmented within the same or longer time frame (56 min). Scale bar, 10 μm. (B) Box-violin plot of the live imaging performed in (A) shows distribution of the POS particle size under different conditions at the start of the imaging (0 min) and at the end (56 min). (C and D) Snapshots from time-lapse live fluorescence imaging performed with an inverted Leica confocal microscope (SP5-mp) on hESC-RPE and EX2-RPE plated in 96-well plates, and treated with 30% serum and SiR-actin (D) or LysoTracker (C), to monitor POS internalization or co-localization with lysosomes, respectively. The complete time lapse can be found in Video S2 (EX2-RPE) and Video S3 (hESC-RPE). Following priming with 30% serum W-POS were added to the cells and imaging started 25 min thereafter without a washing step in between. (C) In hESC-RPE, W-POS (green arrow) were fragmented into smaller pieces and co-localized with lysosomes (orange arrow). In contrast, W-POS remained intact in EX2-RPE and did not co-localize with lysosomes. (D) Actin labeling showed many internalized POS fragments in hESC-RPE (yellow arrow). Some POS fragments were captured during the process of internalization, which takes around 30 min (white arrow). (E) Quantification of the number of POS that co-localize with lysosomes at different time points during the time lapse and in different z-planes, where (0) refers to the apical surface of the RPE and (30) refers to the basal. The number of co-localized POS increased with time and shifted toward the basal side of the RPE. (F) The number of POS/cell in hESC-RPE over the different z-planes and time points during the time lapse showed a shift in the peak of POS/cell count toward basal z planes with time, reflecting internalization. This shift was not detected in EX2-RPE.

To monitor the time needed for W-POS fragmentation, we treated wild-type hESC-RPE and *MERTK* mutant EX2-RPE with AF488-labeled W-POS and 30% serum or 5 μg/mL MFGE8 and performed live imaging of the apical surface of RPE cells with an upright confocal microscope. To monitor actin changes on the surface of the RPE, we labeled the cells with SiR-actin in culture. Three hours after W-POS addition, fragmentation was observed in live hESC-RPE, but not EX2-RPE (Figure 7A; Video S1a) or MFGE8-treated hESC-RPE (Video S1b). When zooming in on particles that were still unfragmented we could observe live in culture W-POS fragmentation within 52 min by hESC-RPE. Quantification of the size (area) of POS in the fluorescence time-lapse images showed that they had a reduced size in hESC-RPE compared with EX2-RPE treated with serum or hESC-RPE treated with MFGE8 already at the start of imaging, 3 h after POS addition (0 min). The size of POS diminished further after 56 min in hESC-RPE treated with serum, but not in the other conditions (Figure 7B). To monitor uptake and co-localization of POS particles with lysosomes, we plated the cells in 96-well plates and performed live imaging with an inverted confocal microscope from the basal side of the cells. Cells were either labeled with SiR-actin or LysoTracker, which labels lysosomes. Co-localization of POS with lysosomes was observed in hESC-RPE, but not in EX2-RPE treated with 30% serum (Figures 7C and 7E; Videos S2 and S3). Interaction of actin-rich filaments with POS and internalization of smaller fragmented particles was observed in hESC-RPE, and lasted around 30 min (Figure 7D; Video S2). As expected, no internalization was observed in EX2-RPE, instead W-POS accumulated at the apical side of the cells (Figure 7F; Video S3). These results show that, under ensheathment conditions, fragmentation of W-POS occurs within approximately 50 min after binding, followed by internalization and co-localization with lysosomes within approximately 30 min. POS fragmentation and internalization were not observed under conditions where ensheathment is not observed, which indicates that ensheathment is required for POS fragmentation before internalization. Taken together, *MERTK* mutation in RPE causes a deficiency in POS fragmentation and phagocytosis, implicating both processes and their interconnection as pathologies that can contribute to vision loss in patients with RP38.

Video S1a. Time Lapse of hESC-RPE and EX2-RPE Cultured in Transwells and Treated with Whole POS and 30% Serum, Related to Figures 7A and 7B

Video S1b. Time Lapse of hESC-RPE Cultured in Transwells and Treated with Whole POS and 5 μg/mL MFGE8, Related to Figures 7A and 7B

Video S2. Time Lapse of hESC-RPE Cultured in 96-Well Plates and Treated with Whole POS and 30% Serum, Related to Figures 7C–7F

Video S3. Time Lapse of EX2-RPE Cultured in 96-Well Plates and Treated with Whole POS and 30% Serum, Related to Figures 7C and 7F

## Discussion

In this study, we established a human RPE-based model to perform mechanistic studies of POS ensheathment and phagocytosis by healthy and diseased RPE. Using this model we observed that MERTK ligands, GAS6 and PROS1, stimulate POS phagocytosis and ensheathment, and that loss of MERTK function leads to loss of both cellular processes. These findings were demonstrated in two CRISPR/Cas9-mediated *MERTK* knockout hESC-RPE and in RP38 patient iPSC-derived RPE. Rescue of MERTK expression, by CRISPR/Cas9-mediated integration of *MERTK* under the *CMV* promoter in the *MERTK* genomic region, led to regain of ensheathment-mediated fragmentation and phagocytosis of POS. This shows that the loss of ensheathment phenotype observed in patient cells is due to the genomic deletion identified in the *MERTK* coding sequence, and that ensheathment is MERTK dependent.

Although serum is not physiologically present in the IPM, both GAS6 and PROS1, which are also present in serum (Hall et al., 2005, Jiang et al., 2017), are expressed and secreted by the RPE in the IPM *in vivo* and stimulate POS phagocytosis (Hall et al., 2005, Karl et al., 2008). Phagocytosis ligands are often supplemented exogenously to boost POS phagocytosis *in vitro* (Law et al., 2015). Our hESC-RPE are differentiated and cultured in serum-free conditions, which is known to enhance endogenous GAS6 expression (Karl et al., 2008). In addition, our RNA sequencing data shows that both PROS1 and GAS6 expression are upregulated in hESC-RPE compared with hESCs. However, the levels of endogenously expressed GAS6 and PROS1 might not be sufficient, or require additional extrinsic signals for their secretion, to significantly induce ensheathment.

POS ensheathment *in vivo* occurs at the site of phagocytosis (Matsumoto et al., 1987). However, its role during phagocytosis is not clear. Here we show that, similar to POS phagocytosis, ensheathing RPE membranes get actively engaged in binding to the POS through receptor-mediated mechanism, leading to a more specific particle recognition. In contrast, 0.72-μm latex beads, which are internalized through caveolae-mediated non-specific endocytosis (Rejman et al., 2004) and independently of MERTK (Carr et al., 2009), did not engage with RPE ensheathing membranes. Addition of MFGE8 to wild-type RPE, which leads to αVβ5 integrin activation (Nandrot et al., 2007), increased both F-POS binding and internalization, which is in line with previous studies (Law et al., 2015). Surprisingly, MFGE8 did not stimulate POS ensheathment in wild-type RPE. Instead, POS landed passively on microvilli, and got internalized in the absence of ensheathment through a yet unidentified mechanism. In contrast, addition of GAS6, which is known to bind and activate MERTK (Law et al., 2015), triggered both POS ensheathment and internalization. Thus, MERTK activation drives ensheathment and phagocytosis, while αVβ5 integrin drives phagocytosis in the presence of an intact MERTK receptor. Consistently, we observed that serum failed to stimulate ensheathment or phagocytosis in *MERTK* mutant RPE even in the presence of an intact αVβ5 integrin receptor, which shows that activation of the latter alone is insufficient to induce both cellular mechanisms and indicates that activation of MERTK and ensheathment might be involved in a process upstream to αVβ5 integrin activation and POS internalization, such as POS fragmentation from the photoreceptors. To test this hypothesis, we isolated W-POS, both round-oval and elongated, which probably originate from rods and cones, respectively. We observed that RPE cells do not readily engulf W-POS, but rather fragment them using ensheathing membranes before internalization. The physiological reason for that might be that RPE are not able to ingest very big particles, such as W-POS (Irschick et al., 2004). W-POS ensheathment and ensheathment-mediated fragmentation by hESC-RPE occurred in the presence of 30% serum or 5 μg/mL GAS6, but not MFGE8, and was absent in *MERTK* mutant RPE cells. The presence of a small percentage of fragmented W-POS in the absence of ensheathment might be due to POS breaking during sample processing before addition to the cells (Parinot et al., 2014). These results provide a mechanistic evidence that POS ensheathment might be necessary to “bite-off” POS fragments as eatable-sized portions from the photoreceptors, and that MERTK activation is required for ensheathment-mediated fragmentation of POS before internalization.

Here, we present two POS models to study the interaction between POS and RPE. We observed that F-POS are more homogeneous in size, and are ensheathed, internalized and degraded faster than W-POS because of the obvious advantage that they do not have to be fragmented first by the RPE. Thus, F-POS are useful, when setting up a fluorescence-based screening assay where time and homogeneous POS size matter. On the other hand, W-POS are more physiological and useful to study basic mechanisms and answer disease-related questions. Ensheathment of W-POS occurs independently from the presence of the entire photoreceptor cell, which overcomes the need to have sophisticated RPE-photoreceptor cells co-culture techniques and increases the throughput of such assays. Thus, having both models at hand might facilitate the identification of new mechanisms and therapeutic targets.

Loss of ensheathment contributes to failure in retinal reattachment in pathologies with retinal detachment (Wickham et al., 2012). More recently, loss of ensheathment has been also shown in a canine model of Best vitelliform macular dystrophy (BVMD), leading to retinal detachment and degeneration, and subsequent loss of vision (Guziewicz et al., 2017). Based on our findings that POS ensheathment is disturbed in *MERTK* mutant RPE, the pathology of RP38 might follow a similar path to that of BVMD. It is quite likely that vision loss in RP38 patients is due to lack of POS ensheathment in preparation for their internalization. Thus, improving ensheathment might be a potential therapeutic strategy for these patients and others suffering from retinal diseases, where phagocytosis might be impaired, such as BVMD (Guziewicz et al., 2017), and age-related macular degeneration (Inana et al., 2018).

Taken together, our findings reveal exciting aspects of the molecular machinery controlling essential photoreceptor-RPE interactions, and present hPSC-RPE as a powerful *in vitro* model to study POS ensheathment and renewal, which are essential for visual function. With our RPE models, a more detailed characterization of the molecular events leading to POS ensheathment and its pathology in retinal degenerative diseases can now be undertaken.

## Experimental Procedures

### Stem Cell Lines and RPE Generation

RPE derived from H9 hESC cell line (hESC-RPE) were used as wild-type control. Fibroblasts were isolated from skin biopsies from RP38 patients and were reprogrammed using the non-integrating Sendai virus. RPE differentiated from patient iPSC is referred to as “MERTK-RPE” throughout the manuscript. *MERTK* gene editing with CRISPR/Cas9 was done in H9 hESCs. Two modified hESC lines were obtained EX2 and EX14. Differentiated RPE is referred to as “EX2-RPE” and “EX14-RPE” throughout the article. Detailed description of the generation of *MERTK* knockout cell lines, and patient iPSC line, and the characterization of the pluripotent cell lines is found in Supplemental Experimental Procedures under the section “Generation and characterization of *MERTK* mutant hPSC lines.” An isogenic control for the patient iPSC line was generated as described in the “Generation of the isogenic control” section in Supplemental Experimental Procedures. RPE derived from isogenic iPSC are referred to as TSS-RPE in some parts of the manuscript.

All RPE cells were differentiated on Transwell filters as described previously (Zhu et al., 2013). RPE cells were passaged for expansion two times on Transwells before use. For some experiments and as indicated in the experimental schemes, RPE cells were passaged to 384- or 96-well plates and used within 13 days. Passaging details can be found in Supplemental Experimental Procedures.

### POS Isolation

POS were isolated from porcine eyes, as described in Molday et al. (1987) and Parinot et al. (2014) with some modifications (Figure S1A). More details on POS isolation and fluorescence labeling can be found in Supplemental Experimental Procedures.

### Phagocytosis Assays

For all experiments, cells were primed with the different treatments for 1 h before the addition of POS. F-POS particles were sonicated in 500 μL RPE medium, containing the different treatments (serum, MFGE8, GAS6, PROS1, VTN), for 5 s 10% power with Branson Digital Sonifier 450, before addition to the cells. POS were quantified using the Neubauer chamber combined with fluorescence microscopy imaging and cell profiler analysis. Around 30 F-POS/cell and 10 W-POS/cell were seeded on the cells. More details on phagocytosis assays variations can be found in Supplemental Experimental Procedures or experimental figure schemes and legends.

### SEM

RPE cells cultured on Transwell filters were fixed with modified Karnovsky's solution, containing 2% glutaraldehyde plus 2% paraformaldehyde in 0.1 M phosphate buffer (pH 7.4) until processing. Processing and imaging details can be found in Supplemental Experimental Procedures.

### Quantification and Statistical Analysis

#### Data and Statistical Analysis

Two-way ANOVA test was used to calculate the statistical significance of the immunoblot data. One-way ANOVA test was used to calculate statistical significance of fluorescence-based phagocytosis assay. SEM images were blinded during quantification. ANOVA test was used for SEM data analysis as indicated in the figure legends. The following formulas were used to analyze the SEM data:

$$Ensheathment\;per\;cell=\frac{Total\;number\;of\;ensheathing\;membranes}{Total\;number\;of\;cells}$$

$$Unensheathed\;F-POS\;per\;cell=\frac{Total\;number\;of\;unensheathed\;POS}{Total\;number\;of\;cells}$$

$$\%\;cells\;with\;sheets=\frac{Number\;of\;cells\;presenting\;sheets}{Total\;number\;of\;cells}$$

$$\%\;ensheathment\;of\;w-POS\;at\;3\;hrs=\frac{Number\;of\;ensheathed\;POS}{Total\;number\;of\;POS}$$

$$\%\;fragmentation\;of\;w-POS\;at\;5\;hrs=\frac{Number\;of\;fragmented\;POS}{Total\;number\;of\;POS}$$

Reagent sources and catalog numbers and further methods can be found in Supplemental Experimental Procedures in the “Key resources table.”

### Study Approval

Permission to work with hESCs was granted by the Robert Koch Institute, Berlin, Germany (license number AZ 3.04.02/0103-A01). The fibroblasts were isolated under full patient consent and approved by Columbia University under IRB protocol number AAAR0284. All procedures were in accordance with the Declaration of Helsinki.

## Authors Contributions

Conceptualization, S.A.; Investigation, S.A., S.S., T.K., and K.V.; Patient iPSC Derivation, S.H.T.; Support in iPSC Reprogramming and Genomic Engineering, S.K. and K.N.; Writing – Original Draft, S.A.; Writing – Review & Editing, all authors; Funding Acquisition, S.A., E.M.T., M.O.K., and M.A.

## Supplemental Information

Document S1. Supplemental Experimental Procedures and Figures S1–S7

Table S1. RNA Sequencing Data Presented in Figure S6D

Document S2. Article plus Supplemental Information
