# Supplementary material for: MERTK-Dependent Ensheathment of Photoreceptor Outer Segments by Human Pluripotent Stem Cell-Derived Retinal Pigment Epithelium
Source: Stem Cell Reports. 2020 Mar 10;14(3):374–89. doi: 10.1016/j.stemcr.2020.02.004 (PMC7066375; doi:10.1016/j.stemcr.2020.02.004)
Supplement: Document S1. Supplemental Experimental Procedures and Figures S1–S7 [file mmc1.pdf]

Stem Cell Reports, Volume *14*

Supplemental Information

MERTK-Dependent Ensheathment of Photoreceptor Outer Segments by Human Pluripotent Stem Cell-Derived Retinal Pigment Epithelium

Seba Almedawar, Katerina Vafia, Sven Schreiter, Katrin Neumann, Shahryar Khattak, Thomas Kurth, Marius Ader, Mike O. Karl, Stephen H. Tsang, and Elly M. Tanaka

Supplemental data items: Figures and Legends

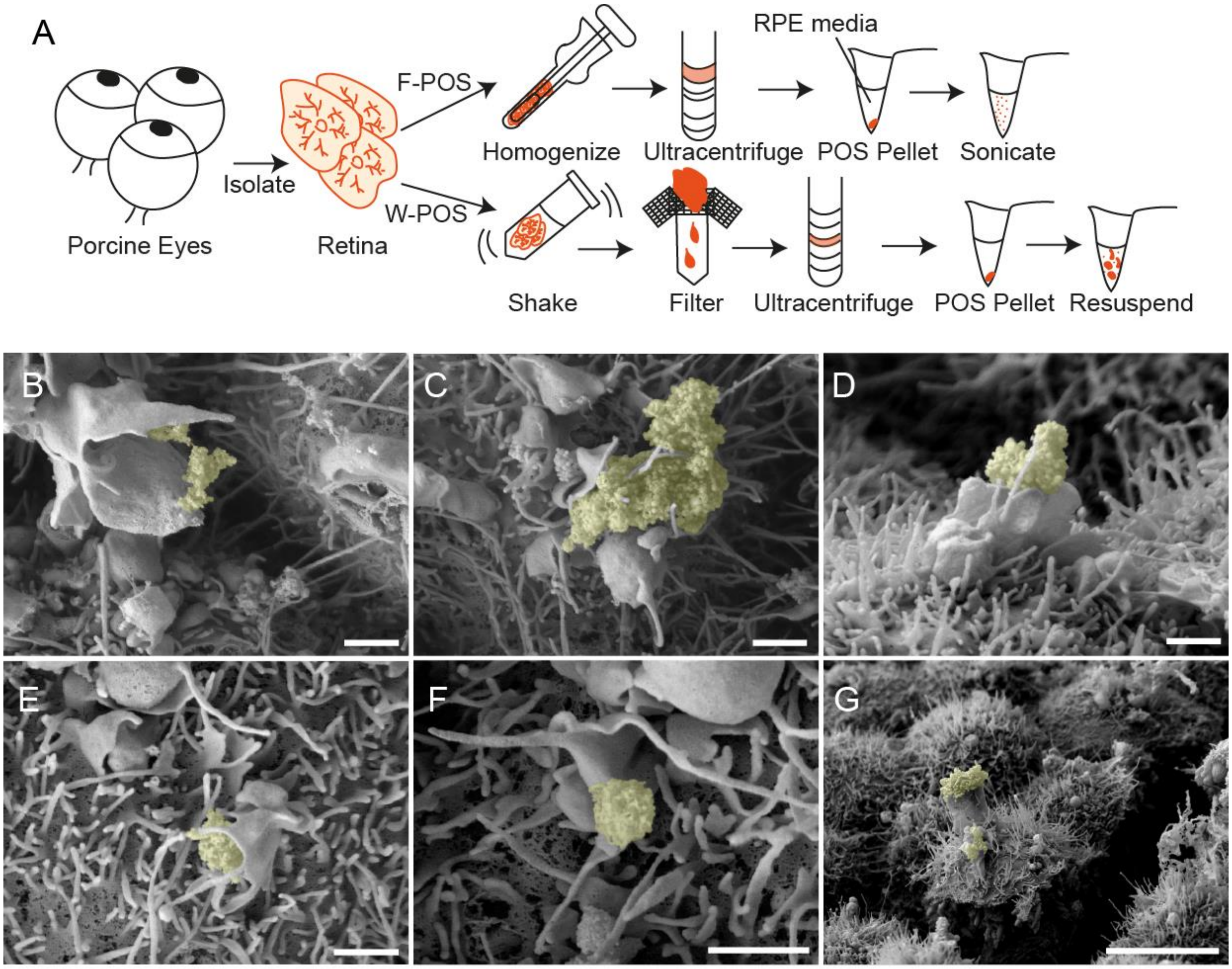

Figure S1. HESC-derived RPE recapitulate dynamic POS ensheathment in vitro. Related to Figure 2. (A) POS isolation scheme. Retinal tissue was isolated from porcine eyes and homogenized with a dounce glass tissue homogenizer. The homogenized retinal tissue was split in 6 tubes containing a sucrose gradient of 27%, 33%, 41%, 50% and 60%, and subjected to ultracentrifugation. The orange band was collected, centrifuged and the pellets were resuspended with POS storage solution and stored at -80ºC. For phagocytosis experiments, POS pellets were resuspended with RPE media and sonicated before addition to the cells. POS obtained using this method are around 0.5-1 µM in size and are referred to as fragmented POS (F-POS) throughout the manuscript. To obtain whole full-sized POS particles, isolated porcine retinas were shaken thoroughly in homogenization buffer, filtered through double layers of gauze, split in sucrose gradient tubes as before and subjected to ultracentrifugation. The faint orange band was collected from each gradient, and centrifuged. The pellets were resuspended with POS storage solution and stored at -80ºC. For phagocytosis experiments POS pellets were resuspended with RPE media and added to the cells without a sonication step. POS obtained using this method are 5-10 µM in size and are referred to as whole POS (W-POS) throughout the manuscript. (B-G) En face SEM images of hESC-RPE cells challenged with F-POS (yellow artificially-colored) shows the different forms of POS ensheathment seen after treatment with stimulants of POS phagocytosis such as serum. Scale bars: B-F: 1 µm, G: 10 µm.

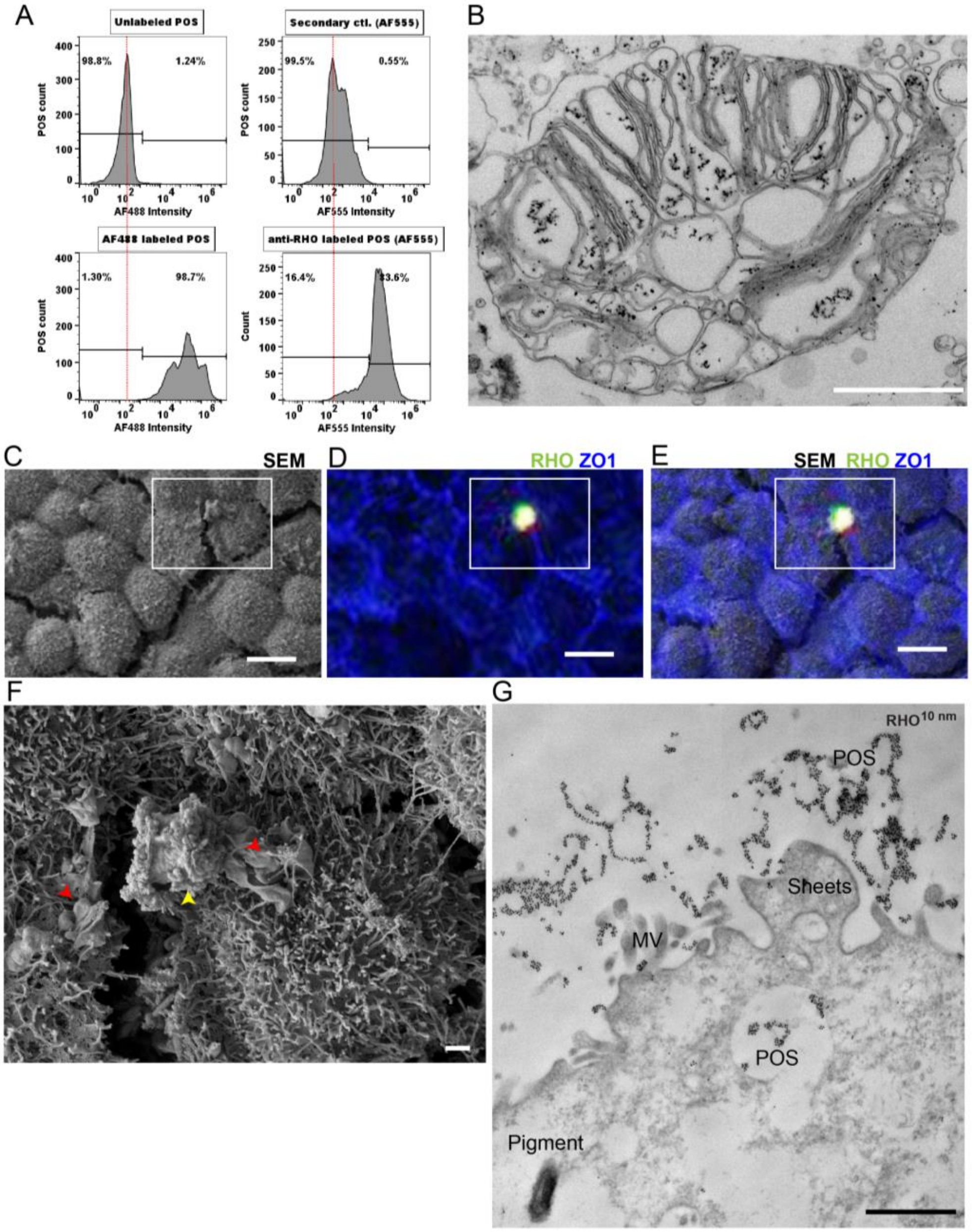

Figure S2. POS quality validation. Related to Figure 2. (A) FACS sorting of POS particles that were either unlabeled, labeled with Alexa Fluor 488 dye (AF488), labeled with Rhodopsin (RHO) antibody followed by secondary (AF555) or labeled with the secondary only (AF555) as a control. The gate of the AF488 labeled POS was set according to the unlabeled POS, while the one for RHO labeled POS was set according to the secondary control. FACS data showed that 98,7% of the POS were labeled with AF488, and 83,6% of the POS were RHO positive. (B) TEM analysis of isolated porcine POS. Membrane discs could still be observed. Scale bar 1 µm. (C) SEM of cells treated with F-POS and serum after immunolabeling with RHO. (D) Fluorescence image of the same area shown in C. Cells were immunolabeled with antibodies against ZO1 (blue) and POS (green) prior to critical point drying and sputter coating for SEM. (E) Overlay of SEM in (C) and fluorescence image in (D) shows that RHO positive POS seeded on cells co-localized with a POS particle surrounded by sheets as seen with SEM. Scale bar 10µm. (F) Higher magnification of C showed that sheets (red arrow) and not microvilli interact with RHO positive particles (yellow arrow). Scale bar 1 µm. (G) TEM analysis of F-POS seeded on cells. POS particles were labeled with anti-RHO and protein A 10 nm gold on ultrathin Lowicryl resin sections. RHO-positive POS were seen on the surface interacting with microvilli (MV) and membrane sheets or internalized within phagosomes. Scale bar 1 µm.

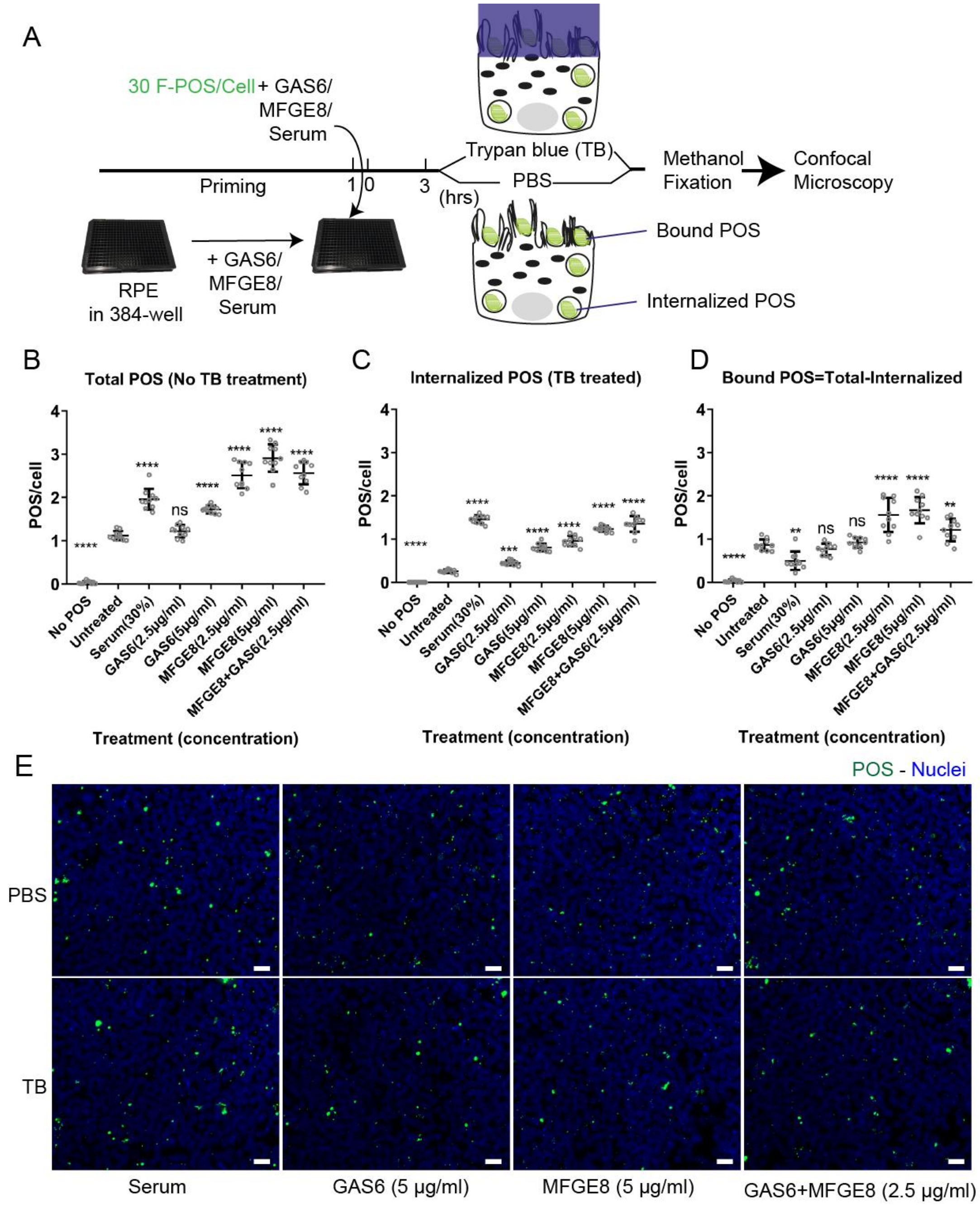

Figure S3. MFGE8 and GAS6 stimulate POS phagocytosis in human RPE. Related to Figure 5.
(A) A schematic of the experiment. HESC-RPE cells (grown on 384 well plates) were primed for 1 hour with either 30% serum, MFGE8, GAS6, or MFGE8 and GAS6 together at different concentrations. Next, cells were challenged with FITC labeled F-POS and incubated for 3 hours at 37ºC. At 3 hours, wells were either treated with 0.4% Trypan Blue (TB) to quench external POS signal or PBS. Samples were then washed and fixed with methanol. Finally, imaging was done using a confocal microscope. (B-D) The median between 6 fields and the mean of the medians of the different repeats was calculated. Data are represented as the mean ± SD. n=10 technical repeats. Significance was calculated using one way ANOVA test. All treatments were compared to untreated. ns >0.05, *<0.05, ** <0.01, ***<0.001, ****<0.0001. (B) Quantification of the number of POS/cell in wells that were not treated with TB to measure total POS (bound and internalized). (C) Quantification of the number of POS/cell in wells that were treated with TB to measure internalized POS. (D) Quantification of the number of bound POS/cell. Serum, GAS6 and MFGE8 increased total POS/cell count. MFGE8 increases POS binding and internalization, while serum and GAS6 increase mainly internalization. (E) Representative fluorescence images of the different conditions. Scale bar: 20 µm.

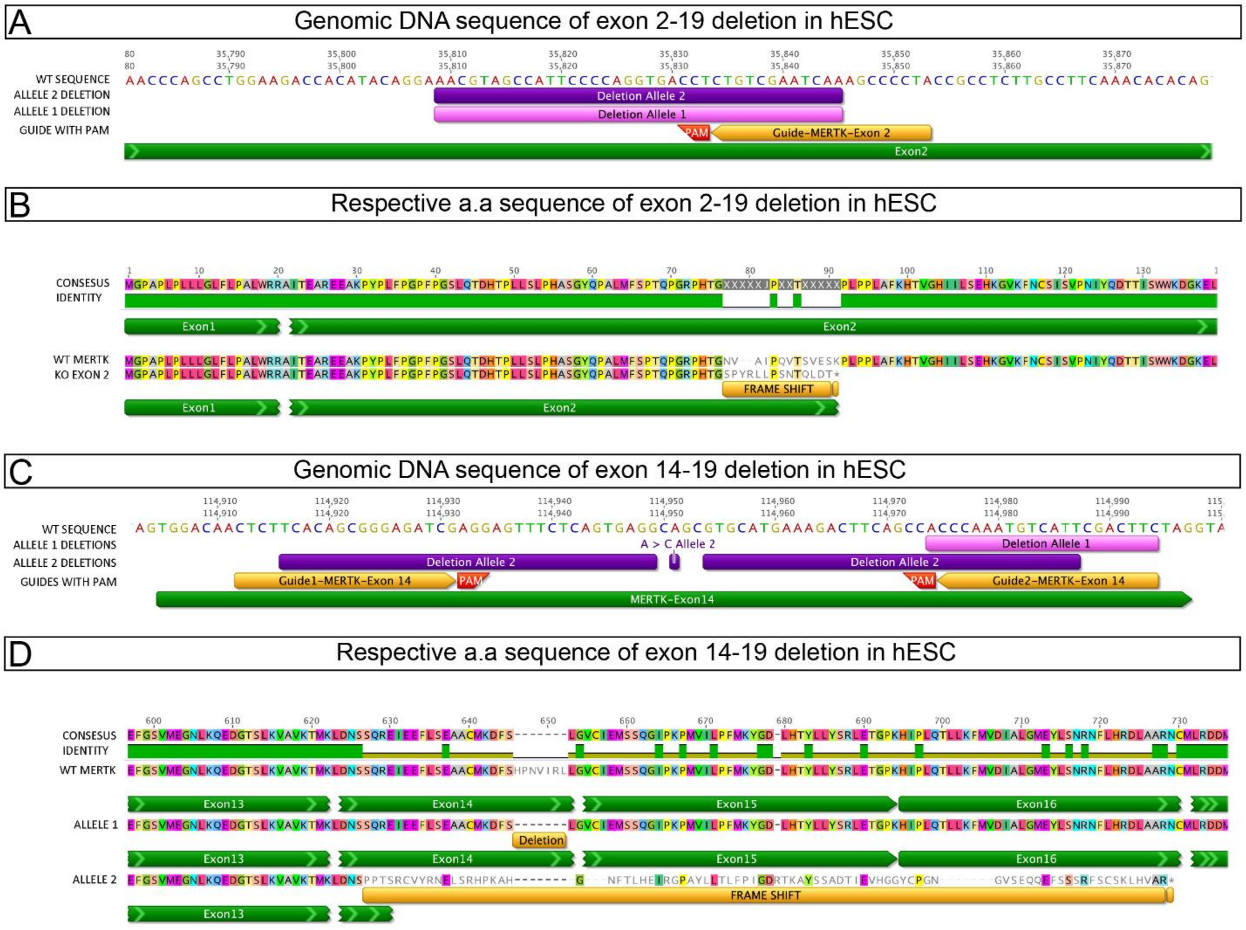

Figure S4. DNA and predicted amino acid sequence of *MERTK* in CRISPR/Cas9 genetically engineered hESC. Related to Figure 5. (A) *MERTK* exon 2 DNA sequence illustrating the deleted region in hESC based on allele specific sequencing. Top row shows the wild-type *MERTK* DNA sequence of exon 2 as shown by green arrow (bottom). The deletions location in the first and second MERTK alleles are marked in purple and pink. Yellow lines indicate the guides, while red boxes represent the respective PAMs for each guide. (B) Predicted amino acid sequence following genomic deletion in exon 2. Yellow rectangles represent the frameshift resulting from the nucleotide deletions in both alleles, while * depicts a premature translational STOP. (C) *MERTK* exon 14 DNA sequence illustrating the deleted region in hESC based on allele specific sequencing. Top row shows the wild-type *MERTK* DNA sequence of exon 14 as shown by green arrow (bottom). The deletions location in the first and second MERTK alleles are marked in pink and purple. Yellow lines indicate the guides, while red boxes represent the respective PAMs for each guide. (D) Amino Acid Sequence alignment of both MERTK alleles in EX14. Predicted amino acid sequence following genomic deletion in exon 14. Yellow rectangles represent the predicted deletion in allele 1 of exon 14 and the frameshift resulting from the nucleotide deletions in allele 2. * depicts a premature translational STOP.

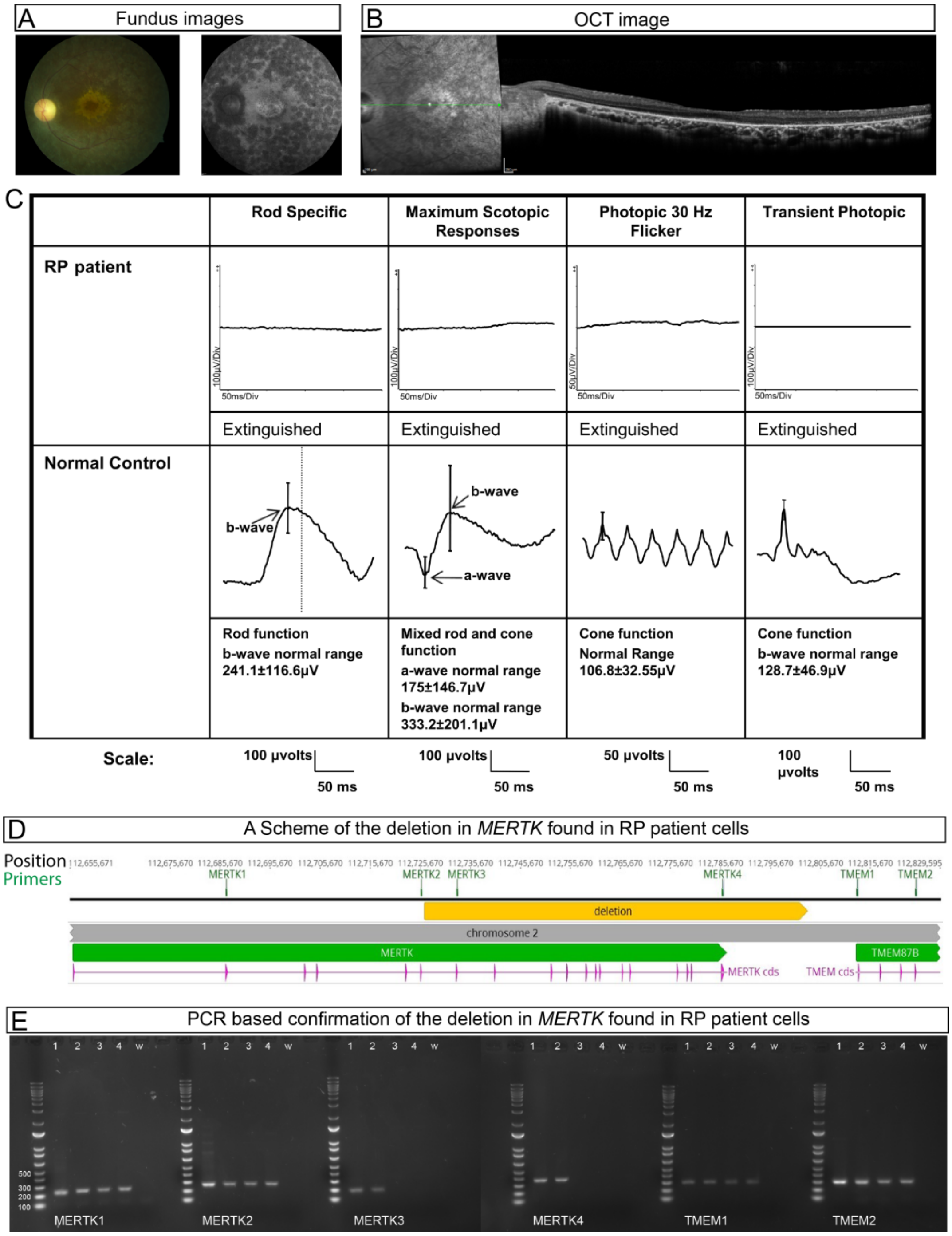

Figure S5. RP38 patient characterization. Related to Figure 5. (A) Fundus images of RP38 patient showed the migration of pigmented cells to the periphery and macular degeneration. (B) OCT image of RP38 patient showed the macular degeneration and thinning of the retina. (C) Full-field ERG was performed using Burian-Allen contact electrodes and with ganzfeld stimulation. All recordings showed very low amplitudes in the patient compared to the normal control. (D) Illustration of the deletion in *MERTK* found in the cells obtained from RP38 patient and the annealing location of the primer pairs used to confirm the mutation: Mertk1-4, TMEM 1-2. (E) Amplification result of the different primer pairs on genomic DNA from (1) a reference genome, (2) hESC, (3) patient iPSC clone 1, (4) patient iPSC clone 2. Primer pairs Mertk 3 and 4 showed no bands in the patient cell lines and a band in the wild-type DNA confirming the deletion.

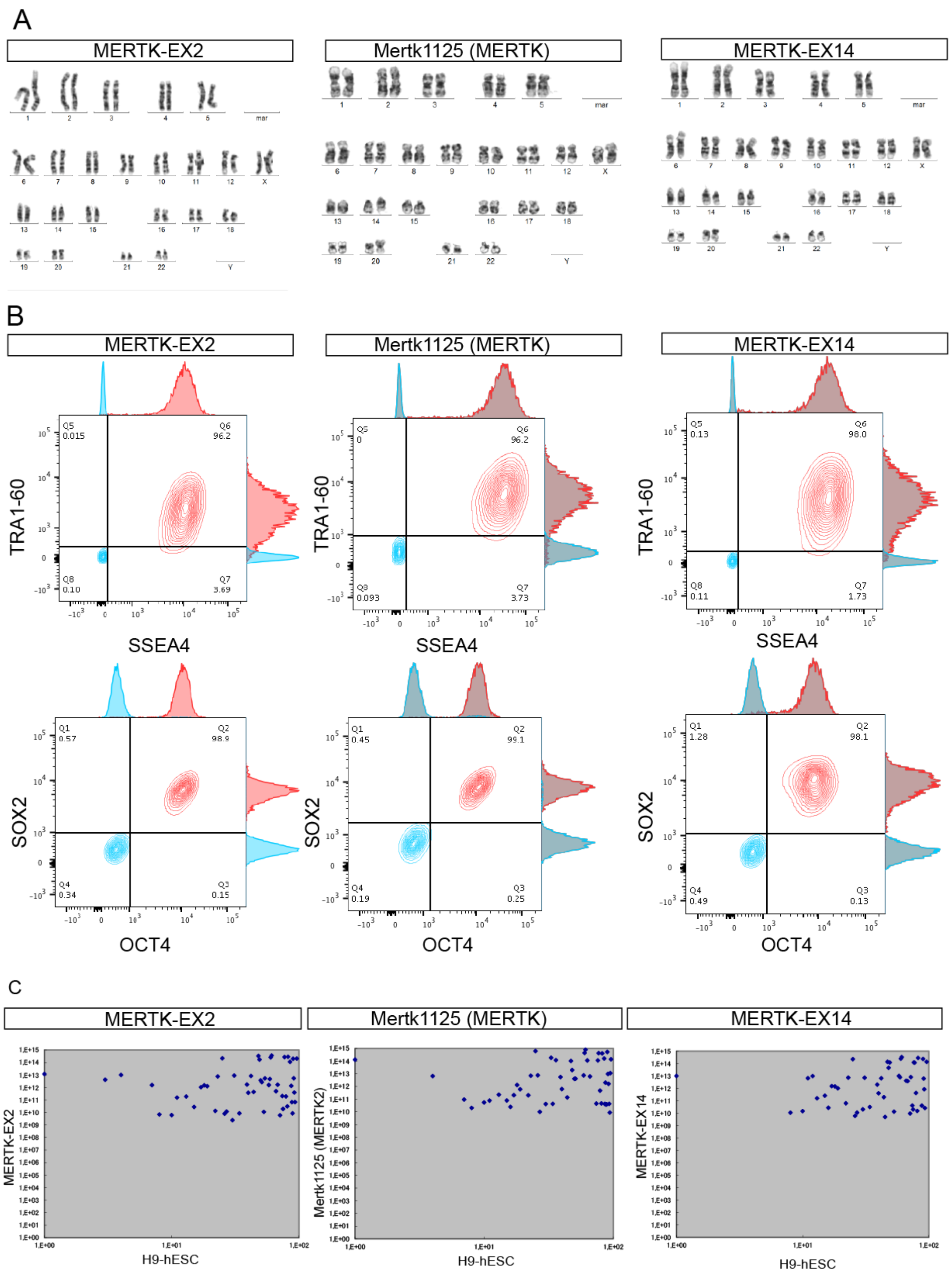

Figure S6. Characterization of iPSC and *MERTK* knock out cell lines. Related to Figure 5. (A) MERTK (clone Mertk1125); MERTK-EX2 and MERTK-EX14 had normal Karyotype. (B) FACS analysis showed that all stem cell lines expressed pluripotency markers OCT4, SOX2, TRA, and SSEA4. (C) Q-PCR analysis of pluripotency markers compared to H9 hESC using PrimerArray® Embryonic Stem Cells (Human) kit.

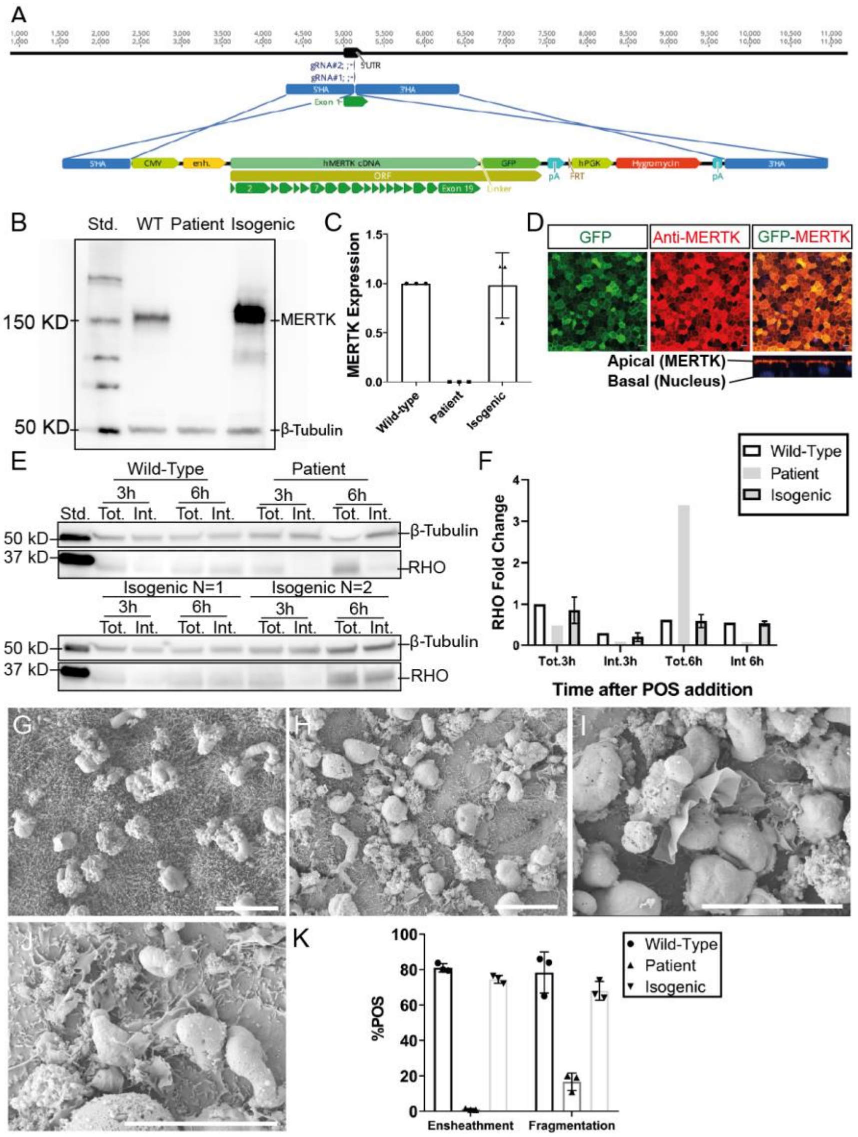

Figure S7. Rescue of MERTK expression in patient RPE restored POS phagocytosis and ensheathment. Related to Figure 5 and 6. (A) Genomic modification scheme in patient iPSC to recover MERTK expression. Isogenic control was created by integrating human MERTK cDNA under the CMV promoter in the MERTK locus using CRISPR/Cas9 system (see supplementary methods **Generation of the isogenic control**). 3' and 5'HA: 3' and 5' homology arms. gRNA: guide RNA. ORF: open reading frame. pA: polyadenylation signal. FRT: Flippase recognition target. hPGK: human Phosphoglycerate kinase promoter. (B) Immuno-blot analysis of MERTK expression in wild-type (WT) RPE (hESC-RPE), patient RPE (MERTK-RPE), and isogenic control RPE (TSS-RPE). MERTK is not expressed in patient RPE, while it is over expressed in the isogenic control. (C) Quantification of MERTK expression in A. Data are represented as the mean ± SD. N=3 Biological repeats. (D) Confocal microscopy fluorescence images of isogenic RPE showing MERTK-GFP (green) expression and Immuno-labeled anti MERTK (red). Overlay of MERTK-GFP and anti MERTK images is thus orange. Orthogonal view shows that MERTK-GFP is correctly localized to the apical membrane of the RPE. Nuclei are shown in blue. (E) Immuno-blot analysis of F-POS phagocytosis in wild-type RPE, patient RPE, and isogenic control RPE. Patient RPE fail to internalize POS after six hours, while isogenic RPE have internalization levels similar to wild-type RPE. (F) Quantification of immuno-blot in (D). Data of the isogenic control are represented as the mean ± SD. N=2 biological repeats. (G-J) SEM images. Scale bar: 10 µm. (G) Patient RPE shows no ensheathment or ensheathment mediated fragmentation of W-POS. (H) Isogenic RPE shows rescue of W-POS ensheathment and fragmentation defect in patient RPE. (I) W-POS ensheathment in isogenic RPE. (J) Ensheathment mediated fragmentation of W-POS in isogenic RPE. (K) Quantification of SEM images. N=3 biological replicates.

Supplemental data items: Excel table Legend

Tables S1. RNA sequencing data presented in **Figure S6D**. RPKM values are shown. Log-FC (fold change) shows the fold change in gene expression in RPE compared to hESC.

Supplemental data items: Videos Legends

Video S1a. Related to Figure 7a-b. Time-lapse of hESC-RPE and EX2-RPE cultured in transwells and treated with whole POS and 30% serum. Time-lapse was performed with an upright confocal microscope, LSM880, using the Airy scan function. ACTIN was labeled with SIR-Actin (magenta). POS were labeled with AF488 (green). Images were taken every 4 minutes. Of note is how the majority of POS were fragmented at the start of imaging (0 minutes), which was 3 hours after POS addition, while POS in EX2-RPE remained largely intact. When zooming in on POS, which were still not fragmented at the start of imaging (0 minutes), we observed fragmentation within 52 minutes in hESC-RPE, but not EX2-RPE. Scale bar: 10 µm.

Video S1b. Related to Figure 7a-b. Time-lapse of hESC-RPE cultured in transwells and treated with whole POS and 5µg/ml MFGE8. Time-lapse was performed with an upright confocal microscope LSM880 using the Airy scan function. ACTIN was labeled with SIR-Actin (magenta). POS were labeled with AF488 (green). Images were taken every 4 minutes. Of note is how most of the POS were not fragmented at the start of imaging (0 minutes), which was 3 hours after POS addition, nor at the end of imaging (92 minutes). Scale bar: 10 µm.

Video S2. Related to Figure 7 C-F. Time-lapse of hESC-RPE cultured in 96 well plates and treated with whole POS and 30% serum. Time-lapse was performed with an inverted confocal microscope, Leica Sp5MP. In one well, ACTIN was labeled with SIR-Actin (red), and in another well cells were labeled with Lysotracker (red) to monitor POS co-localization with lysosomes. POS were labeled with AF488 (green). Images were taken every 5 minutes. The same time-lapse is shown from different views to appreciate POS binding, fragmentation, internalization and co-localization with lysosomes.

Video S3. Related to Figure 7 C and F. Time-lapse of EX2-RPE cultured in 96 well plates and treated with whole POS and 30% serum. Time-lapse was performed with an inverted confocal microscope, Leica Sp5MP. **C**ells were labeled with Lysotracker (red) to monitor POS co-localization with lysosomes. POS were labeled with AF488 (green). Images were taken every 5 minutes. The same time-lapse is shown from different views. POS fragmentation, internalization and co-localization with lysosomes could not be observed.

Supplemental Experimental Procedures:

Key resources table		
**Reagent or Resource**	**SOURCE**	**IDENTIFIER**
**Antibodies**		
Alexa Fluor 488 anti-Oct3/4	Pharmingen	560253 BD
PE anti-Sox2	Pharmingen	560291 BD
V450-SSEA-4	Horizon	561156 BD
Alexa Fluor 647 anti-Tra-1-60	Pharmingen	560850 BD
Alexa Fluor® 488 Mouse IgG1 κ Isotype Control	Phosflow	557782 BD
PE Mouse IgG2a, κ Isotype Control	Pharmingen	555574 BD
V450 Mouse IgA, κ Isotype Control	Horizon	562142 BD
Alexa Fluor® 647 Mouse IgM, κ Isotype Control	Pharmingen	560806 BD
rhodopsin (Immunofluorescence)	Sigma	O4886
rhodopsin (Immuno-blot)	Abcam	ab5417
ZO1	Invitrogen	402200,
Bestrophin1	Abcam	ab2182
MITF	Abcam	ab122982
MERTK	Abcam	ab52968
EZRIN	Santa Cruz	sc-58758
β-TUBULIN	Thermo Fisher	PA5-16863
Alexa Fluor 488 rabbit	Invitrogen	A21206
Alexa Fluor 488 mouse	Invitrogen	A21202
Alexa Fluor 647 rabbit	Invitrogen	A31573
Alexa Fluor 647 mouse	Invitrogen	A31571
HRP-rabbit secondary antibody	Invitrogen	32260
HRP- mouse secondary antibody	Thermo Fisher	62-6520
rabbit anti mouse bridging antibody	Sigma	M7023_2ML
protein A gold	CMC Utrecht	https://www.cellbiology-utrecht.nl/products.html
**Plasmids**		
MERTK plasmid	Sino Biologicals Inc	HG10298-ACG
**Biological Samples**		
RP38 patient fibroblasts	This study	N/A
**Chemicals, Peptides, and Recombinant Proteins**		
soybean trypsin inhibitor	Sigma	T6522
trypsin-EDTA	Gibco	T3924
ACTIVIN A	R&D	338-AC-050
Antibiotic-Antimycotic	Gibco	15240-062
Cas9 NLS, *S. pyogenes* protein	New England Biolabs	M0646
TrypLE	Thermo Fisher	12563011
protease inhibitors	Roche	04693132001
Fluorescein	Invitrogen	F2182
Alexa Fluor 488	Invitrogen	A20000
Alexa Fluor 555	Invitrogen	A20009
Alexa Fluor 647 Phalloidin	Invitrogen	A22287

GAS6	R&D systems	885-GSB
MFGE8	R&D systems	2767-MF
PROS1	R&D systems	948-PS-100
VTN	R&D systems	2308-VN-050
Serum (FBS)	gibco	26140-079
trypan blue	Sigma	T8154
Paraformaldehyde	Science Services	E15714-S
Glutaraldehyde	Science Services	E16220
Hoechst 33342	Thermo Fisher	H1399
RIPA	Thermo Fisher	89900
BSA	SERVA	11926.03
10% Mini-PROTEAN® TGX™ Precast Protein Gels	BioRad	4561033
Precision Plus Protein™ WesternC™	BioRad	1610376
SuperSignal™ West	Thermo Fisher	34075
Precision Protein™ StrepTactin-HRP Conjugate	BioRad	1610380
Lowicryl K4M resin	Science Services	14330
osmium tetroxide	Science Services	19190
Protonex beads	ATT Bioquest	21209
mTESR1	StemCell Technologies	85870
SIR-Actin Kit	Cytoskeleton, Inc	CY-SC001
LysoTracker™ Deep Red	Invitrogen	L12492
Dispase	StemCell Technologies	07923
Rock inhibitor Y-27632	StemCell Technologies	78003
Hygromycin B	ThermoFisher Scientific	10687010
**Critical Commercial Assays**		
CytoTune TM-iPS 2.0 Sendai Reprogramming Kit	Thermo Fisher	A16518
human ES cell Primer Array	Takara Clontech	PH016
EnGen sgRNA Synthesis kit, S. pyogenes	New England Biolabs	E3322
P3 Primary Cell 4D-Nucleofector kit	LONZA	
QIAamp DNA Mini kit	Qiagen	51304
NEB PCR Cloning kit	New England Biolabs	E1202
RNeasy Mini kit	Qiagen	74106
NEBNext Poly(A) mRNA Magnetic Isolation Module	NEB	E7490L
NEBNext Ultra Directional RNA Library Prep Kit for Illumina	NEB	E7420L
XP bead purification	Beckman Coulter	A63882
Qubit dsDNA HS Assay Kit	Invitrogen	Q32854
NEBuilder® HiFi DNA Assembly Cloning Kit	NEB	E5520S
EnGen® sgRNA Synthesis Kit	NEB	E3322S
EnGen® Spy Cas9 NLS, NEB, M0646M		
Primary Cell 4D-Nucleofector Kit L (V4XP-3024, Lonza)		
**Deposited Data**		
RNA seq Raw and analyzed data	This paper www.ncbi.nlm.nih.gov/geo/	GSE127352
**Experimental Models: Cell Lines**		
H9 human embryonic stem cell line	WiCell	WA-09
MERTK knockout in H9 (MERTK-EX2)	This study	N/A
MERTK knockout in H9 (MERTK-EX14)	This study	N/A

RP38 patient iPSC (MERTK)	This study	N/A
TSS isogenic iPSC control	This study	N/A
**Software and Algorithms**		
CRISPR Design Tool	Hsu et al., 2013	crispr.mit.edu
CCTop	Stemmer et al., 2015	https://crispr.cos.uni-heidelberg.de/
Geneious software	Kearse *et al.*, 2012	https://www.geneious.com/
GraphPad Prism	La Jolla California USA,	www.graphpad.com
Knime	Berthold et al., 2009	
Cell Profiler	Lamprecht et al., 2007	
GSNAP (v2014-12-17)		http://research-pub.gene.com/gmap.
featureCounts (v1.4.6)	Liao et al., 2014	http://subread.sourceforge.net/
R		https://www.r-project.org/
DESeq2 R package (v1.6.3)	Love et al., 2014	https://bioconductor.org/packages/release/bioc/html/DESeq2.html
**Other**		
384 well plates	Greiner	781091
Transwells	Corning	3470
Nalgene centrifuge tubes	Thermo Fisher	3137-0050

Passaging of RPE cells

For passaging, RPE on transwells were incubated with trypsin-EDTA (TE) for ten minutes at 37ºC and 5% CO2. After incubation, cells were vigorously pipetted to obtain single cells, and transferred to a tube containing RPE medium plus soybean trypsin inhibitor. Next, RPE medium containing TE was removed by centrifugation at 180 g for two minutes. Finally, cells were resuspended with RPE medium containing 10-20 ng/ml ACTIVIN A and 1x Antibiotic-Antimycotic, and 45,000 cells per one 384 well, 200,000 cells per one 96 well plate or 300,000 cells per transwell were plated.

Immunofluorescence labeling of RPE cells

Cells were fixed with 4% paraformaldehyde (PFA) in 0.1 M phosphate buffer pH 7.4 (PFA-PB). After several washes with PBS, they were treated with a quenching solution (1xPBS, 100 mM Glycine, 0.3% Triton X-100) for twenty minutes and then were incubated with a blocking solution (1xPBS, 1% BSA, 0.3% Triton X-100) for one hour. The primary and secondary antibodies were diluted in the blocking solution and incubated overnight at 4°C. Cell nuclei were counterstained by addition of Hoechst 33342 (1mg/ml) to the secondary antibody solution. The primary antibodies and their working dilutions were: EZRIN (1:100), ZO1 (1:200), BEST1 (1:500), MITF (1:500), MERTK (1:1000). The secondary antibodies Alexa Fluor 488/647 rabbit and mouse were used at 1:500 dilution. Phalloidin 647 was added to the cells after the secondary antibody (1:1000).

Confocal imaging

RPE cells grown in transwells were cut out from the well, transferred on slides and imaged using SP5-MP confocal microscope. Alternatively, CV7000S confocal microscope was used for imaging RPE cells in 384 well plates.

POS isolation

POS were isolated from porcine eyes, as described in (Molday, Hicks and Molday, 1987) with some modifications **(Figure S1 A)**. Briefly, retinal tissue was isolated from 50 porcine eyes and homogenized with a Dounce glass tissue homogenizer in a buffer containing protease inhibitors, 0.2 mM Tris-HCl, 0.1 mM Glucose, 130 mM $NaCl_2$, 0.1 mM Taurine, and 0.02 mM $MgCl_2$. The homogenized retinal tissue was split in 6 tubes containing a sucrose

gradient of 27%, 33%, 41%, 50% and 60%, and centrifuged in the Beckmann Coulter ultracentrifuge B409 at 28000 rpm for one hour at 4ºC. The orange band was collected in Nalgene centrifuge tubes, and centrifuged at 13000 g for ten minutes. Finally, the pellets were resuspended with POS storage solution containing protease inhibitors, 10mM phosphate buffer pH 7.2, 100 mM $NaCl_2$ and 2.5% sucrose and stored at -80ºC until they were used. POS obtained using this method are around 0.5-1 µM in size and are referred to as fragmented POS (F-POS) throughout the manuscript. To obtain whole length POS particles, isolated porcine retinas were shaken thoroughly in homogenization buffer containing 20% sucrose, 20mM Tris Acetate, 2mM MgCl2, 10 mM glucose, and 5 mM Taurine. Then retinal tissue was filtered 3 times through double layers of gauze as originally described (Molday, Hicks and Molday, 1987). The filtered retinal tissue was split in 6 tubes containing a sucrose gradient of 27%, 33%, 41%, 50% and 60%, and centrifuged in the Beckmann Coulter ultracentrifuge B409 at 28000 rpm for one hour at 4ºC. The faint orange band was collected from each gradient, and diluted 5x with wash buffer containing, 10% sucrose, 20mM sodium phosphate buffer and 5 mM Taurine, and centrifuged at 4800 rpm for ten minutes. Finally, the pellets were resuspended with POS storage solution containing DMEM with 2.5% sucrose and stored at -80ºC until they were used as described in (Parinot *et al.*, 2014). POS obtained using this method are 5-10 µM in size and are referred to as whole POS (W-POS) throughout the manuscript.

POS labeling

For labeling, Fluorescein (FITC) or Alexa Fluor 488 or 555 (AF488, AF555) were added to the POS after thawing for one hour at 25°C with shaking (500 rpm). Next, F-POS were centrifuged at 9000 rcf at 4°C for 10 minutes, while W-POS were centrifuged at 3600 rcf for 5 minutes. Pellets were washed twice with the washing buffer, containing; 10% sucrose, 20 mM phosphate buffer pH7.2 and 5 mM Taurine.

POS phagocytosis assay variations

To distinguish total and internalized F-POS by means of fluorescence imaging, 0.4% trypan blue was added to half of the wells for 10 minutes and then washed six times with PBS to remove leftover dye before methanol fixation for 5 minutes, as described previously (Mao and Finnemann, 2013). Six images from each replicate were acquired covering the different areas in the well. Acquired images were imported into the Cell Profiler software for image analysis. POS and nuclei were segmented and values of the number, size and intensity were obtained. The data obtained from image analysis was imported into KNIME for statistical analysis. GraphPad Prism was used for statistical significance calculations and final graph presentation. The median count of the POS between the 6 images was divided by the median count of cells per well to obtain the POS/cell count.

To distinguish total and internalized F-POS by means of immuno-blot, half of the samples were treated with 2 mM EDTA for ten minutes at 37 ºC to remove bound POS from the cells before lysis and the other half was lysed without treatment as described previously (Mao and Finnemann, 2013).

Immuno-blots

Around one million cells were lysed with RIPA buffer for 30 minutes at 4°C. Next, the protein lysates were separated from the pellet by centrifugation and around 50 µg of protein was loaded into Mini-PROTEAN® TGX™ Precast Protein Gels. 10 µl of Precision Plus Protein™ WesternC™ was loaded as a standard. Proteins were transferred into PVDF membrane using TE70 Semi-dry transfer unit. Blocking was done for one hour at room temperature in 5% milk powder dissolved in TBST. To look at MERTK expression in the different wild-type and *MERTK* mutant RPE, primary antibody incubation was done with MERTK (1:1000) antibody recognizing the N-terminal of the protein and β-TUBULIN (1:1000) antibody overnight at 4°C in 5% milk-TBST. HRP-rabbit secondary antibody (1:1000) was added for one hour at room temperature in 5% milk-TBST. To analyze POS phagocytosis by means of Immuno-blot membranes were incubated with anti-RHO antibody (1:1000), which recognizes the C-terminal of the protein, followed by HRP-mouse secondary antibody (1:1000). Precision Protein™ StrepTactin-HRP conjugate was also added with the secondary to view the standard in chemiluminescence mode. The blot membrane was incubated with SuperSignal™ West according to Manufacturer´s instructions and imaged with LAS4000.

Live imaging of POS phagocytosis

Two confocal systems were used for POS phagocytosis live imaging. The first system was LSM 880 upright using the Airyscan detector. Dipping objective with 40X magnification was used. Time lapse images were processed with Airyscan Processing method in the ZEN-black software. HESC-RPE and EX2-RPE (grown on transwells) were primed with 30% serum or 5µg/ml MFGE8 for 1 hour, and then they were challenged with AF488 labeled W-POS and left for two-and-a half hours at 37°C with 5% CO2 before imaging. Membranes, containing the RPE monolayer, were cut out from the transwell insert and placed with the apical side of the cells facing the dipping objective in a 35

mm cell culture dish with 2 ml media containing SiR-Actin dye and 30% serum. Images were taken every four minutes. Alternatively, live fluorescence imaging was performed with an inverted Leica confocal microscope (SP5-mp) on hESC-RPE and EX2-RPE plated in 96 well plates, and treated with 30% serum and SiR-Actin or Lysotracker, to monitor W-POS internalization or co-localization with lysosomes respectively. Following priming with 30% serum POS were added to the cells and imaging started without a washing step in between. In both systems, sample preparation and setting up imaging parameters usually took around 30 minutes. Image analysis was done with the Cell Profiler software (Lamprecht, Sabatini and Carpenter, 2007) and data analysis and figure preparation was done with KNIME (Berthold *et al.*, 2009).

Transmission Electron Microscopy (TEM)

POS pellets were either fixed in modified Karnovsky's solution and embedded in Epon as previously described (Zhu *et al.*, 2013), or fixed in 4% PFA-PB for immuno-EM.

For immuno-EM, the samples were fixed in 4% PFA in 0.1 M phosphate buffer pH 7.4. After several washes in PBS, samples were dehydrated and infiltrated in Lowicryl K4M resin using the progressive lowering of temperature (PLT) method (Carlemalm, Garavito and Villiger, 1982). After polymerisation, the blocks were raised up to room temperature, and 70 nm sections were cut on a Leica UC6 ultramicrotome. Sections were mounted onto copper mesh grids for immunolabeling. On-section labeling of ultrathin sections was performed as previously described (Fabig *et al.*, 2012). In brief, sections were blocked with 1% BSA in PBS, incubated for one hour with primary antibodies mouse anti-RHO (1:200) in BSA/PBS. Next, they were washed with PBS, and incubated with bridging antibody (rabbit anti mouse, 1:100 in BSA/PBS) for thirty minutes. Sections were then washed with PBS, incubated with protein A 10 nm gold (1:100) for one hour in BSA/PBS, washed with PBS, post-fixed in 1% glutaraldehyde/PBS, washed with water, stained with 4% uranyl acetate in water, washed with water and dried for TEM inspection. Ultrastructure and gold labeling was imaged with a FEI Morgagni 268D or a Jeol JEM1400 Plus both at 80 kV acceleration voltage.

SEM sample processing:

SEM samples were fixed in modified Karnovsky (2% glutaraldehyde, 2% formaldehyde in 50 mM HEPES), washed with PBS, and post-fixed in 1% osmium tetroxide in PBS for two hours on ice. Next, they were washed with water and dehydrated in a graded series of ethanol/water starting from 30% and up to 100% ethanol on molecular sieve. Then, they were critical-point dried using the Leica CPD 300 (Leica Microsystems, Vienna, Austria), cut out from the transwell, mounted on 12 mm aluminium stubs, and sputter-coated with gold using the Baltec SCD 050 (Leica Microsystems, Vienna, Austria). Finally, filters were analyzed with a Jeol JSM 7500F cold field emission SEM (Jeol, Eching, Germany) at 5 kV acceleration voltage using the lower secondary electron detector. To correlate fluorescence images of rhodopsin positive POS and SEM images, POS were labeled with AF555 and added to hESC-RPE. After three hours the cells were fixed with 4% PFA, and labeled with anti-ZO1 antibody and anti-RHO antibodies followed by anti-mouse AF488. Then, they were post fixed with 4% PFA for twenty minutes, washed in PBS, dehydrated and critical-point dried. Samples were mounted on stubs and imaged first with an upright confocal fluorescence microscope (SP5-I), and then they were sputter-coated with gold and imaged with the SEM.

Generation and characterization of *MERTK* mutant hPSC lines

Reprogramming of patient fibroblasts and genomic engineering in iPSC was done in collaboration with the Stem Cell Engineering Facility in the CRTD. Two *MERTK* knockout hESC lines; namely EX2 (homozygous deletion from Exon 2 onwards), and EX14 (heterozygous deletion of Exon 14 onwards), were generated using CRISPR/Cas9 in hESCs. Nucleotides homozygous deletion in exon 2 was confirmed by allele specific sequencing **(Figure S4 A)**. The deletion resulted in a frameshift mutation and premature stop in both allele as shown in the predicted amino acid sequence **(Figure S4 B)**. In exon 14, two deletions were confirmed by allele specific sequencing **(Figure S4 C)** resulting in two amino acid sequence predictions for each allele. In the first allele (Allele 1) amino acids 646 – 652 (HPNVIRL) were deleted. In the second allele (Allele 2), nucleotides deletion resulted in a frameshift mutation and a premature stop **(Figure S4 D)**.

Guides were designed and finalized by using the Geneious software or the online guide prediction tools; including CRISPR Design Tool, and CCTop. In vitro transcribed guides were generated using the EnGen sgRNA Synthesis kit. We chose guides that were common and showed the least off-target, in the virtual tools. The sequences of the guides used to generate Exon 14 and Exon 2 deletion along with respective PAMs can be found in the table below.

Oligonucleotide sequence	SOURCE
Exon14 Guide 1: CTCTTCACAGCGGGAGATCG AGG	This Study
Exon14 Guide 2: GAAGTCGAATGACATTTGGG TGG	This Study
Exon2 Guide: TAGGGGCTTTGATTCGACAGAGG	This Study

S. pyogenes Cas9 NLS (5.5 µg) was mixed with 1 µg of each in vitro transcribed guide RNA, and incubated for twenty minutes at room temperature before they were electroprated into H9 hESC single cell suspension, using Lonza's 4D-Nucleofector and the P3 Primary Cell 4D-Nucleofector kit.

Colonies were picked and screened by Sanger sequencing (Yusa, 2013) of the *MERTK* Exon 2 or Exon 14 regions. Colonies showing indels at the guide sites were individually expanded. Genomic DNA was extracted from the indel positive clones with the QIAamp DNA Mini kit', and was used as a template to amplify the region flanking the Exon 14 of *MERTK* gene using F-primer1 and R-primer2.

Oligonucleotide sequence	SOURCE
F-primer1: 5' AGAAAGTACAGAGAATGCCCATAGGTCC 3'	This Study
R-primer2: 5' ACAGCATCCACTCCATACTTAATCTCC 3'	This Study

The resulting fragment (~ 1.2 kb) was cloned using the NEB PCR Cloning kit and sequenced to determine allele specific sequences. Similarly, F-primer3 and R-primer4 were used for amplification of genomic fragments (~0.5 kb) for cloning and sequencing Exon 2 deletion.

Oligonucleotide sequence	SOURCE
F-primer3: 5' TTCTTGGCCTAAGAAGTTGGGAACCTACTTGG 3'	This Study
R-primer4: 5' GAACACATACCTGAAGGAAGCGATTATTGCTG 3'	This Study

RPE was differentiated from MERTK-EX14 and MERTK-EX2 knockout hESC cell lines as previously described (Zhu *et al.*, 2013; Zhu, Schreiter and Tanaka, 2014), and is referred to as EX14-RPE and EX2-RPE in this study.

To validate the phenotype observed in *MERTK* knockout cell lines, an iPSC cell line was derived from a patient diagnosed with RP38, harboring a homozygous genomic deletion in *MERTK*. The patient (female) was diagnosed at 12 years of age with RP38. She had night blindness, and on dilated fundus exam, she had a bull´s eye pattern of atrophy on both maculae **(Figure S5 A-B)**. Peripheral fundus showed extensive mottling and intra-retinal pigment migration. The optic nerve was symmetric with a good rim but with temporal pallor. Optical coherence tomography (OCT) images showed thinning of the outer nuclear layer, and almost no RPE layer was left in the macula region. The patient was prescribed vitamin A supplements. However, peripheral vision deteriorated over time and the patient complained of photopsia (constant light flashes). Electroretinogram (ERG) responses were extinguished **(Figure S5 C)**. Genetic analysis of patient derived fibroblasts, using the Affymetrix Cytoscan HD array, showed that the patient had a homozygous loss in the DNA region 2q13 corresponding with the *MERTK* gene with the coordinates 112,726,509-112,803,074 based on the GRCh37 assembly. The 77 bp deletion of hMERTK (exons 7-19) is illustrated in **Figure S5 D**. The deletion was confirmed in both fibroblasts and fibroblast-derived iPSC by means of PCR **(Figure S5 E).** The primers used for PCR based confirmation are listed in the table below:

Primer	Position in Chr 2	Sequence	Amplicon Size (bp)
MERTK-1-fwd	112,686,816<br>112,686,835	CCTGCCTTGATGTTTTCACC	228
MERTK-1-rev	112,687,043<br>112,687,024	TTCCTTCCCATCTTTCCACC	
MERTK-2-fwd	112,725,762<br>112,725,781	ACAGCATTCTGATCTCCTGG	256
MERTK-2-rev	112,726,017<br>112,725,998	ACACAGTATCATGGGCAGAG	
MERTK-3-fwd	112,732,993<br>112,733,012	ATGAAATAGGCTGGTCTGCA	176

MERTK-3-rev	112,733,168<br>112,733,149	CTCCTCCAGAGACAAAGTCC	
MERTK-4-fwd	112,785,993<br>112,786,012	GGCTGCAGCTAGAAAAACTC	355
MERTK-4-rev	112,786,347<br>112,786,328	CAGGAGACCCCATTCCTAAC	
TMEM-1-fwd	112,812,901<br>112,812,920	CACATCCTGGCTCCTATCTC	275
TMEM-1-rev	112,813,175<br>112,813,156	CGACCATCTTGACCAGGAA G	
TMEM-2-fwd	112,824,616<br>112,824,635	GTGATTCACAGGTGTTTCCC	294
TMEM-2-rev	112,824,909<br>112,824,890	CCCAGCCACGTATCTTACTT	

Patient iPSC and *MERTK* hESC knockout cell lines showed a normal karyotype **(Figure S6 A)**. They also expressed high levels of pluripotency markers as determined by flow cytometry **(Figure S6 B)**. The following antibodies were used: Alexa Fluor 488 anti-Oct3/4, PE anti-Sox2, V450-SSEA-4, and Alexa Fluor 647 anti Tra-1-60. Isotype antibodies (resources table) and unstained cells were used for gating. Antibodies dilutions that were recommended by the manufacturer were used. The antibodies with the recommended dilutions did not label fibroblasts, which are negative for these markers. Pluripotency of the three hPSC lines was also confirmed by qRT-PCR according to the International Stem Cell Initiative International (ISCI) using the human ES cell Primer Array **(Figure S6 C)**.

Generation of the isogenic control:

MERTK expression in patient RPE was rescued by inserting hMERTK as cDNA near the **t**ranscription **s**tart **s**ite (TSS) into exon 1 of one of the endogenous alleles using CRISPR/Cas9-assisted gene targeting. The TSS targeting vector was generated from a custom synthesized plasmid backbone from ThermoFisher Scientific containing the ColE1 origin of replication and Ampicillin resistance. The insert from this plasmid was removed by HindIII and SfiI based restriction digestion and replaced with 4 PCR fragments using the NEBuilder® HiFi DNA Assembly Cloning Kit. The regions flanking the targeting site at the 5' end (851 bp) and the 3' end (1283 bp) were amplified from RP38 patient iPSC clone hMERTK1125#4 genomic DNA to serve as 5' and 3' homology arms **(Figure S7 A)**. The insert consisting of the CMV promoter followed by an ORF containing hMERTK cDNA with GFP-tag and polyadenylation signal was PCR-amplified from a MERTK plasmid, followed by a custom synthesized FRT-flanked PGK-hygro selection cassette from ThermoFisher Scientific.

SgRNAs targeting the region close to the TSS was *in vitro*-transcribed using EnGen® sgRNA Synthesis Kit. The sgRNA target sites for TSS were excluded from the targeting vector to protect it from CRISPR/Cas9-induced double-stranded breaks.

Patient iPSC clone MERTK1125#4 ($8*10^5$ cells) were electroporated with 10 µg linearized targeting vectors, 2 µg sgRNA and 19 µg Cas9-NLS protein (EnGen® Spy Cas9 NLS) with the Lonza 4D X-unit, pulse CB-150 and the Primary Cell 4D-Nucleofector Kit L and seeded at clonal density on Matrigel-coated dishes in mTeSR1 medium supplemented for 4 days with 10 µM Rock inhibitor Y-27632. Cells were selected with 40 µg/ml Hygromycin B starting on day three after nucleofection and up to six days. Resistant colonies were manually picked, clonally expanded and screened for proper targeting by colony PCR amplifying the 5' and 3' junction of the targeted alleles from outside of the homology arms into the insert. The presence of an intact second allele was also verified. Heterozygous clones were selected and the complete insert was analysed by Sanger sequencing. Clone hMERTK1125-4-TSS #10 showed an intact 46,XX[cp20] chromosome set like the parental line and was used in this manuscript.

**Oligonucleotide sequence**	**SOURCE**
hMERTK-TSS Guide 1: 5'-ACTGCCCGGGCCGCCCGGAC-3'	This Study
hMERTK-TSS Guide 2: 5'-CCCGGGCAGTGAGTGCCGAG-3'	This Study

Next-generation sequencing (RNA-seq)

hESC-RPE was differentiated on transwells as described previously (Zhu *et al.*, 2013; Zhu, Schreiter and Tanaka, 2014) and total RNA was purified from three consecutive RPE and hESC preps, using the Qiagen RNeasy Mini kit. Using the NEBNext Poly(A) mRNA Magnetic Isolation Module, polyadenylated mRNA was enriched from 1 µg total RNA with an integrity number of ≥ 9, according to the manufacturer's instructions. The mRNA was eluted in 15 µl 2x first strand cDNA synthesis buffer (NEBnext), in order to chemically fragment the samples, followed by reverse transcription, second strand synthesis, end repair, A tailing and adapter ligation according to the manual of NEBNext Ultra Directional RNA Library Prep Kit for Illumina. For ligation, hybridized custom adaptors were used (Adaptor-Oligo 1 and 2). Afterwards, excess of non-ligated adapters were depleted by XP beads purification, adding beads in a ratio of 1:1. Finally, samples were indexed during a PCR enrichment step with 15 cycles of amplification using primers carrying a specific index sequence indicated with 'NNNNNN' (Index Primer1, 2 and 3). After two more rounds of XP beads purifications (1:1), libraries were quantified using the Qubit dsDNA HS Assay Kit. For Illumina flowcell production, samples were equimolarly pooled and distributed over all lanes on the Illumina HiSeq 2500 to sequence 75bp single reads.

**Oligonucleotide sequence**	**SOURCE**
Adaptor-Oligo 1: 5'-ACA CTC TTT CCC TAC ACG ACG CTC TTC CGA TCT 3'	This Study
Adaptor-Oligo 2: 5'-P-GAT CGG AAG AGC ACA CGT CTG AAC TCC AGT CAC-3'	This Study
Index Primer1: AAT GAT ACG GCG ACC ACC GAG ATC TAC ACT CTT TCC CTA CAC GAC GCT CTT CCG ATC T	This Study
Index Primer2: GTG ACT GGA GTT CAG ACG TGT GCT CTT CCG ATC T	This Study
Index Primer3: CAA GCA GAA GAC GGC ATA CGA GAT NNNNNN GTG ACT GGA GTT)	This Study

Next-generation sequencing (RNA-seq) bioinformatics

Resulting reads were mapped with GSNAP (v2014-12-17) to the human genome (hg38) using splice junction information from Ensembl (v81) as support. Uniquely mapped reads were then converted into counts per gene using featureCounts (v1.4.6) and gene annotations from Ensembl (v81). Normalization of the raw counts based on the library size and testing for differential expression between the different cell types/treatments was performed with the DESeq2 R package (v1.6.3). Genes with adjusted p-value (Benjamini-Hochberg) less than 0.05 were considered differentially expressed. RPKM data were generated in R. The heat map presented in supplementary figure S6 was generated using GraphPad Prism. LOG2 of the mean of the RPKM values of the three biological repeats for the genes of interest, was plotted in the heat map shown in **Figure 1**. Individual RPKM values are shown in excel **Table S1.** The raw and analyzed data are deposited in GEO (www.ncbi.nlm.nih.gov/geo/) with the accession number: GSE127352.

Supplemental References:

Berthold, M. R. *et al.* (2009) 'KNIME - the Konstanz information miner', *ACM SIGKDD Explorations Newsletter*, 11(1), p. 26. doi: 10.1145/1656274.1656280.

Carlemalm, E., Garavito, R. M. and Villiger, W. (1982) 'Resin development for electron microscopy and an analysis of embedding at low temperature', *Journal of Microscopy*, 126(2), pp. 123–143. doi: 10.1111/j.1365-2818.1982.tb00362.x.

Fabig, G. *et al.* (2012) 'Labeling of Ultrathin Resin Sections for Correlative Light and Electron Microscopy', *Methods in Cell Biology*, 111, pp. 75–93. doi: 10.1016/B978-0-12-416026-2.00005-4.

Hsu, P. D. *et al.* (2013) 'DNA targeting specificity of RNA-guided Cas9 nucleases', *Nature Biotechnology*. Nature Publishing Group, 31(9), pp. 827–832. doi: 10.1038/nbt.2647.

Lamprecht, M. R., Sabatini, D. M. and Carpenter, A. E. (2007) 'CellProfiler: Free, versatile software for automated biological image analysis', *BioTechniques*, 42(1), pp. 71–75. doi: 10.2144/000112257.

Liao, Y., Smyth, G. K. and Shi, W. (2014) 'featureCounts: an efficient general purpose program for assigning sequence reads to genomic features.', *Bioinformatics (Oxford, England)*, 30(7), pp. 923–30. doi: 10.1093/bioinformatics/btt656.

Love, M. I., Huber, W. and Anders, S. (2014) 'Moderated estimation of fold change and dispersion for RNA-seq data with DESeq2', *Genome Biology*. BioMed Central, 15(12), p. 550. doi: 10.1186/s13059-014-0550-8.

Mao, Y. and Finnemann, S. C. (2013) 'Analysis of Photoreceptor Outer Segment Phagocytosis by RPE Cells in Culture', *Methods in Molecular Biology*, 935, pp. 285–295. doi: 10.1007/978-1-62703-080-9.

Molday, R., Hicks, D. and Molday, L. (1987) ‘Peripherin . A Rim-Specific Membrane Protein of Rod Outer Segment Discs’, *Investigative ophthalmology & visual science*, 28, pp. 50–61.
Parinot, C. *et al.* (2014) ‘Large-scale purification of porcine or bovine photoreceptor outer segments for phagocytosis assays on retinal pigment epithelial cells’, *Journal of Visualized Experiments*, (94), pp. 1–8. doi: 10.3791/52100.
Stemmer, M. *et al.* (2015) ‘CCTop: An Intuitive, Flexible and Reliable CRISPR/Cas9 Target Prediction Tool’, *PLOS ONE*. Edited by S. Maas. Public Library of Science, 10(4), p. e0124633. doi: 10.1371/journal.pone.0124633.
Yusa, K. (2013) ‘Seamless genome editing in human pluripotent stem cells using custom endonuclease-based gene targeting and the piggyBac transposon’, *Nat Protoc*, 8(10), pp. 2061–2078. doi: 10.1038/nprot.2013.126.
Zhu, Y. *et al.* (2013) ‘Three-Dimensional Neuroepithelial Culture from Human Embryonic Stem Cells and Its Use for Quantitative Conversion to Retinal Pigment Epithelium’, *PLoS ONE*, 8(1), p. e54552. doi: 10.1371/journal.pone.0054552.
Zhu, Y., Schreiter, S. and Tanaka, E. M. (2014) ‘Accelerated Three-Dimensional Neuroepithelium Formation from Human Embryonic Stem Cells and Its Use for Quantitative Differentiation to Human Retinal Pigment Epithelium’, *Methods in Molecular Biology (Clifton, N.J.)*, 1307, pp. 345–355. doi: 10.1007/7651_2013_56.
