# Supplementary material for: MERTK-Dependent Ensheathment of Photoreceptor Outer Segments by Human Pluripotent Stem Cell-Derived Retinal Pigment Epithelium
Source: Stem Cell Reports. 2020 Mar 10;14(3):374–89. doi: 10.1016/j.stemcr.2020.02.004 (PMC7066375; doi:10.1016/j.stemcr.2020.02.004)
Supplement: Document S2. Article plus Supplemental Information [file mmc7.pdf]

Stem Cell Reports
Article
OPEN ACCESS

MERTK-Dependent Ensheathment of Photoreceptor Outer Segments by Human Pluripotent Stem Cell-Derived Retinal Pigment Epithelium

Seba Almedawar,[1,6,*] Katerina Vafia,[1] Sven Schreiter,[1] Katrin Neumann,[2] Shahryar Khattak,[2] Thomas Kurth,[2] Marius Ader,[1] Mike O. Karl,[1,3] Stephen H. Tsang,[4] and Elly M. Tanaka[5]
[1]Technische Universität Dresden, Center for Molecular and Cellular Bioengineering (CMCB), Center for Regenerative Therapies Dresden (CRTD), Fetscherstraße 105, 01307 Dresden, Germany
[2]Technische Universität Dresden, Center for Molecular and Cellular Bioengineering (CMCB), Technology Platform, Fetscherstraße 105, 01307 Dresden, Germany
[3]German Center for Neurodegenerative Diseases (DZNE) Dresden, Tatzberg 41, 01307 Dresden, Germany
[4]CUMC/Edward S. Harkness Eye Institute, 635 West 165th Street, New York, NY 10032, USA
[5]Research Institute of Molecular Pathology (IMP), Vienna Biocenter (VBC), Campus Vienna Biocenter 1, 1030 Vienna, Austria
[6]Senior author
*Correspondence: seba.almedawar@tu-dresden.de
https://doi.org/10.1016/j.stemcr.2020.02.004

SUMMARY

Maintenance of a healthy photoreceptor-retinal pigment epithelium (RPE) interface is essential for vision. At the center of this interface, apical membrane protrusions stemming from the RPE ensheath photoreceptor outer segments (POS), and are possibly involved in the recycling of POS through phagocytosis. The molecules that regulate POS ensheathment and its relationship to phagocytosis remain to be deciphered. By means of ultrastructural analysis, we revealed that Mer receptor tyrosine kinase (MERTK) ligands, GAS6 and PROS1, rather than αVβ5 integrin receptor ligands, triggered POS ensheathment by human embryonic stem cell (hESC)-derived RPE. Furthermore, we found that ensheathment is required for POS fragmentation before internalization. Consistently, POS ensheathment, fragmentation, and internalization were abolished in *MERTK* mutant RPE, and rescue of MERTK expression in retinitis pigmentosa (RP38) patient RPE counteracted these defects. Our results suggest that loss of ensheathment due to MERTK dysfunction might contribute to vision impairment in RP38 patients.

INTRODUCTION

The retinal pigment epithelium (RPE) is a monolayer of pigmented epithelial cells that are essential for the function and survival of the overlying retinal photoreceptors and ultimately for the preservation of vision. During early human development, the RPE connects with the undifferentiated photoreceptors via adherens and gap junctions (Fisher and Lindberg, 1975). As soon as the photoreceptors differentiate and form outer segments, these junctions disappear and are replaced by sheets that interdigitate with the newly formed photoreceptor outer segments (POS) in the interphotoreceptor matrix (IPM). POS, found at the tips of photoreceptors, are formed from stacked membrane discs rich in opsin (rhodopsin [RHO] in the case of rods) and enclosed within the photoreceptor plasma membrane. RPE apical membrane protrusions rich in pigment granules extend in the form of “sheets” and wrap around POS in a process termed “POS ensheathment” (Steinberg et al., 1977). RPE ensheathing membranes contribute to the adhesion of the retina to the RPE (Wickham et al., 2012), and are at the site of phagocytosis of POS, which are detached daily from the overlying photoreceptors for recycling with the onset of light (Besharse et al., 1977) and under circadian control (LaVail, 1976). POS renewal and clearance are required to avoid accumulation of oxidized lipids and light-damaged proteins in the IPM and for the recycling of molecules involved in the light cycle (Strauss, 2005). However, it remains unclear whether POS are actively shed by photoreceptors, removed by extrinsic processes, or a combination of both. Of note, *in vivo* histology studies showed that ensheathing RPE membranes also invade POS structure, possibly during or before POS separation, suggesting that RPE might be “biting-off” POS tips, which could be part of the phagocytosis process (Steinberg et al., 1977). The study of POS ensheathment, separation, and phagocytosis has so far been limited by the low availability of primary human eyes, and thus hampered our understanding of the molecules that regulate ensheathment and its relationship to phagocytosis.

POS phagocytosis has been extensively studied *in vitro* and *in vivo* and key receptors and ligands that initiate signaling pathways, leading to phagocytosis, have been identified. Separation of POS leads to the curling of membrane discs (Steinberg et al., 1977) and exposes phosphatidylserines (PS), which are “eat me” signals that bind to CD36 receptors on the apical surface of RPE leading to activation of phagocytosis (Finnemann and Silverstein, 2001). Milk fat globule-EGF8 (MFGE8), secreted by the RPE, binds to exposed PS on POS, and serves as an “opsonin” that bridges POS to αVβ5 integrin receptor (Nandrot et al., 2007), which is found on the apical surface of the RPE. Binding of MFGE8-opsonized POS particles to αVβ5 integrin initiates two downstream signaling pathways. On the

© 2020 The Authors.
This is an open access article under the CC BY license (http://creativecommons.org/licenses/by/4.0/).

one hand, it stimulates Mer receptor tyrosine kinase (MERTK) via αVβ5 integrin-associated focal adhesion kinase (Nandrot et al., 2004). On the other hand, it activates RAC1-GTPase (Mao and Finnemann, 2012), which leads to F-actin recruitment to the phagocytic cup. Vitronectin (VTN) is another ligand for the αVβ5 integrin, but it does not seem to stimulate POS binding and internalization (Nandrot et al., 2007). MERTK is essential for POS phagocytosis (Feng et al., 2002; Karl et al., 2008). Two other ligands secreted by the RPE, namely growth arrest-specific protein 6 (GAS6) and protein S (PROS1), bind to PS on the surface of POS and to MERTK on the surface of RPE, and lead to MERTK phosphorylation and activation (Hall et al., 2001; Law et al., 2015). Phosphorylation of MERTK generates docking sites for Src homology 2 (SH2) proteins, such as phosphoinositide 3-kinases (Shelby et al., 2013), which is required for F-actin recruitment to the phagocytic cup (Bulloj et al., 2013). Previous studies have shown that ensheathing membranes are actin rich, and that blocking actin polymerization prevents POS separation and ensheathment (Matsumoto et al., 1987). Although downstream signaling of both αVβ5 integrin and MERTK leads to actin recruitment, their differential role during POS ensheathment is not known.

Mutations in RPE surface receptors result in phagocytosis defects in animal models of retinal degeneration and in humans. *Mertk* knockout rats (Bok and Hall, 1971) and mice (Duncan et al., 2002) lack a functional MERTK, which causes accumulation of POS in the IPM and subsequent photoreceptor degeneration. Similarly, mutations in *MERTK* lead to retinitis pigmentosa type 38 (RP38) in humans causing early onset retinal degeneration and blindness (Parinot and Nandrot, 2016). At present, the exact role of MERTK during POS phagocytosis, and the molecular and cellular events leading to loss of phagocytosis in its absence have not been fully deciphered.

POS ensheathment has been observed in human RPE explants (Steinberg et al., 1977), but to our knowledge it has not been explicitly studied in cultured human RPE. Human pluripotent stem cell (hPSC)-derived RPE have been shown useful for studying basic disease mechanisms (Ramsden et al., 2017).

In this study, we used hPSC-RPE to reconstitute and analyze homogeneously fragmented POS (F-POS) and whole full-sized POS (W-POS) ensheathment *in vitro* using SEM-based ultrastructural analysis. We observed that RPE cells extend membrane sheets that capture and ensheath F-POS *in vitro* within 3 h in a serum-dependent manner. Surprisingly, MERTK ligands, GAS6 and PROS1, rather than αVβ5 integrin receptor ligands, MFGE8 and VTN, stimulate ensheathment. We further showed that ensheathment participates in fragmenting W-POS before internalization. Consequently, RPE derived from human embryonic stem cells (hESCs), in which *MERTK* was knocked out using CRISPR/Cas9, or from an RP38 patient iPSC cell line with homozygous *MERTK* deletion, failed to ensheath, fragment, and internalize W-POS, which shows that MERTK is required for ensheathment and ensheathment-mediated fragmentation of POS, and implicates the loss of which in RP38 disease pathology and vision loss in patients.

RESULTS

RPE Derived from Pluripotent Stem Cell Lines Display Mature RPE Characteristics

All RPE differentiated from wild-type hESCs, and *MERTK* mutant hESC and hiPSC lines, were highly pigmented (Figure 1A) and expressed mature RPE markers in a polarized fashion, including ZO1, bestrophin 1 (BEST1), MITF, and EZRIN (Figures 1B and 1C). RNA sequencing analysis of hESC-derived RPE (hESC-RPE) compared with hESCs also showed upregulation of mature RPE markers, including phagocytosis-related genes, and downregulation of pluripotency markers (Figure 1D). *MERTK* mutant RPE showed either no expression or very weak expression of MERTK as shown by immunostaining (Figure 1B) and immunoblot analysis (Figures 1E and 1F). Ultrastructural SEM analysis of hESC-RPE showed that cells have a defined hexagonal shape and long microvilli on their apical surface (Figures 2A–2D).

HESC-Derived RPE Recapitulate Dynamic POS Ensheathment *In Vitro*

To characterize POS ensheathment in the RPE we fragmented our POS preparation before addition to the cells to obtain homogeneously sized POS (F-POS) and expose the inner membrane containing eat me signals required for phagocytosis (Figure S1A). In addition, to prepare the RPE cells for the incoming POS, we primed RPE for 1 h with serum, as it is known to induce phagocytosis (Karl et al., 2008). One hour after treatment with F-POS and 30% serum, the membrane of the RPE started to grow around POS (Figures 2E and 2F) and formed sheets that wrapped around them by 3 h (Figures 2G and S1B–S1G). In contrast, RPE treated with POS alone showed no sheet formation even after 24 h (Figure 2H). Treatment with serum in the absence of POS did not result in sheet formation either (N = 3). Quantification of RPE membrane sheets with and without associated POS (Figure 2I) showed that the percentage of cells harboring ensheathing membranes increased with time to reach around 90% after 3 h in the presence of serum, while in its absence the percentage of cells presenting sheets was negligible (Figure 2J). Consistently, the absence of serum led to an increase in the amount of POS that were not ensheathed by the RPE (unensheathed POS) (Figure 2K). Notably, the number of

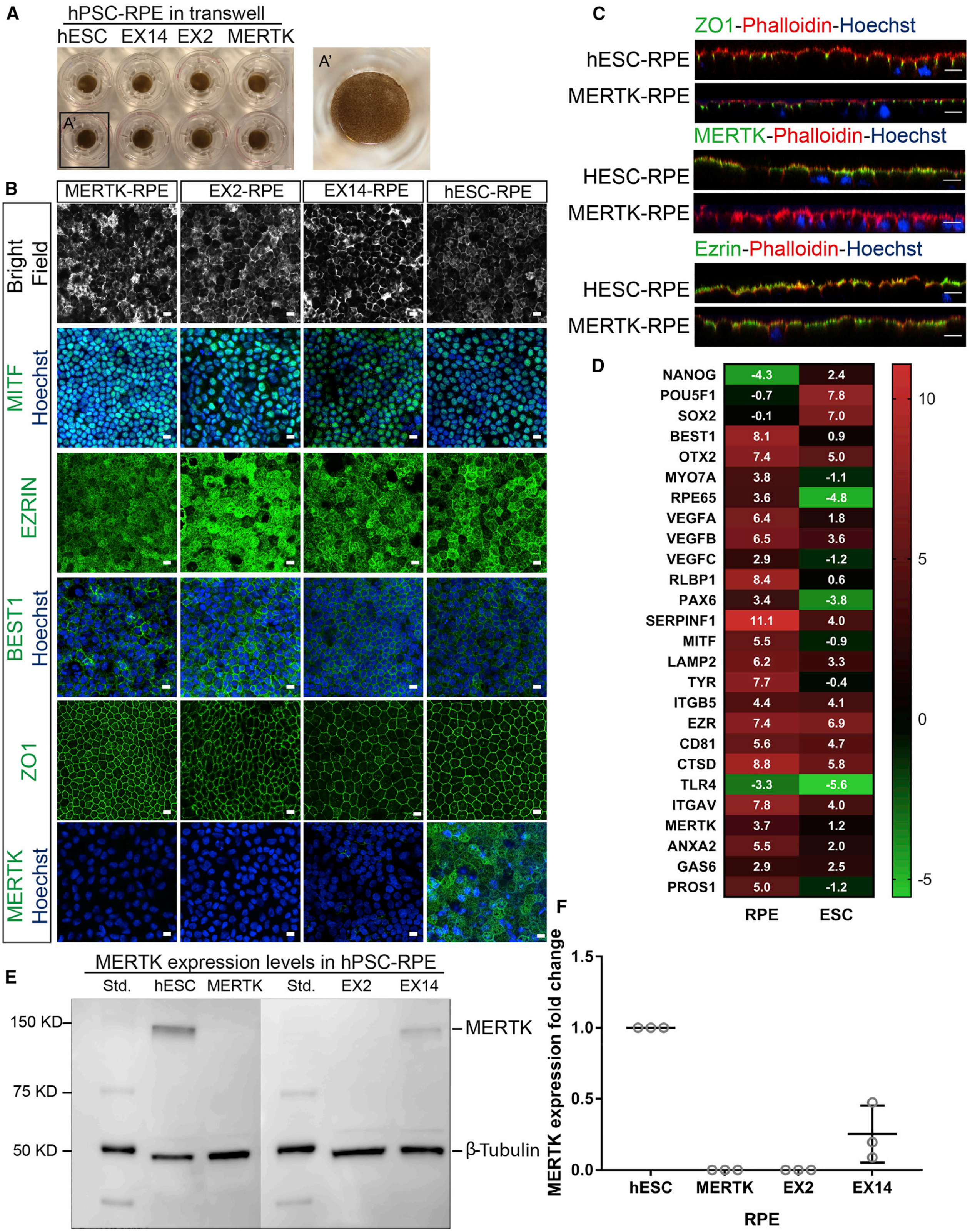

(legend on next page)

ensheathed POS in the presence of serum and the percentage of cells harboring ensheathing membranes following a 3-h pulse decreased gradually to reach minor levels after 6 h, indicating the dynamic nature of the formed sheets. Therefore, POS ensheathment by human RPE can be recapitulated *in vitro* in the presence of serum, which stimulates their formation.

In the mammalian retina, rods are more abundant than cones and are the first to degenerate in MERTK-related retinitis pigmentosa (RP38). The percentage of RHO-positive F-POS (rods outer segment marker) employed in all experiments was around 83% (Figure S2A) and membrane discs were visualized by transmission electron microscopy (TEM) (Figure S2B). RHO-positive F-POS were also seen interacting with membrane sheets by means of correlative fluorescence and SEM (Figures S2C–S2F), and by immunogold labeling in TEM (Figure S2G).

Ensheathment by Human RPE Is Specific for POS

To further validate the established hESC-RPE model, the specificity of membrane sheet formation with regard to F-POS was compared with that for latex beads, which are usually internalized by the RPE through non-specific endocytosis and independently of the presence of serum (Carr et al., 2009). We reasoned that if ensheathment is specific to POS, then none should be observed when beads are added in the presence of serum. As predicted, no sheets were observed at the point of interaction between RPE and beads (Figure 3B). Interestingly, despite the absence of sheet formation, these beads were internalized by the RPE, as seen by TEM analysis (Figures 3C and 3D), suggesting that the unspecific endocytosis pathway does not require ensheathment, and that ensheathment is specific for POS.

MERTK Ligands Rather Than $\alpha V\beta 5$ Integrin Ligands Stimulate POS Ensheathment by Human RPE

MFGE8 and GAS6 are known to stimulate phagocytosis in other RPE models through binding to their canonical receptors MERTK and $\alpha V\beta 5$ integrin, respectively. To validate whether F-POS phagocytosis in hESC-RPE can be induced by serum or known phagocytosis ligands, such as MFGE8 and GAS6, different concentrations of both ligands were compared with serum (Figure S3A). GAS6 and serum stimulated POS internalization in a dose-dependent manner. MFGE8 stimulated both POS binding and internalization at higher concentrations. Serum showed higher POS internalization values than MFGE8 and GAS6 separately. However, combining MFGE8 and GAS6 upregulated internalization similarly to serum (Figures S3B–S3D). Taken together, these results show that POS phagocytosis can be induced by serum, and more specifically by GAS6 and MFGE8, confirming previously known roles of these ligands during phagocytosis in other RPE models.

To determine whether GAS6 and MFGE8 are also involved in triggering F-POS ensheathment similar to phagocytosis, increasing concentrations of GAS6, MFGE8, or serum were added to RPE (Figure 4A). Similar to serum, GAS6 increased POS ensheathment (Figures 4B, 4C, 4E, and 4F). GAS6 is known to bind to and activate MERTK, which suggests that ensheathment, which is stimulated by GAS6 addition, might be mediated through MERTK, and that other ligands that activate MERTK might have the same effect. For this purpose we tested whether PROS1, which is another known ligand for MERTK, might similarly stimulate ensheathment. As expected, PROS1 stimulated POS ensheathment significantly (Figures 4H–4J). In contrast, addition of MFGE8 did not induce POS ensheathment and led to accumulation of unensheathed POS at the apical surface of the RPE (Figures 4D–4G). Furthermore, there was no additive effect on ensheathment when both ligands were added in comparison with GAS6 alone (Figures 4E and 4F). Because MFGE8 is known to bind and activate $\alpha V\beta 5$ integrin receptor, we evaluated whether VTN, another known $\alpha V\beta 5$ integrin receptor ligand, can stimulate ensheathment. Similar to MFGE8, VTN showed very low levels of POS ensheathment, and an increase in the amount of unensheathed POS compared with serum (Figures 4H–4J). Thus, these results suggest that

Figure 1. RPE Derived from Pluripotent Stem Cell Lines Display Mature RPE Characteristics

(A) RPE derived from wild-type and *MERTK* mutant hPSC grown in Transwells.

(B) HPSC-derived RPE are pigmented and express mature RPE markers, including MITF, EZRIN, bestrophin 1 (BEST1), and ZO1. MERTK is expressed in hESC-RPE, but not in EX2 and MERTK-RPE. It is weakly expressed in EX14-RPE. Scale bar, 10 μm.

(C) Cross-sections of immunofluorescence images of RPE markers in hESC-RPE and MERTK-RPE. Note the polarized expression of ZO1, EZRIN, and MERTK on the apical surface of the RPE. Scale bar, 10 μm.

(D) Heatmap of the mean of log2 values of reads per kilobase of transcript per million mapped reads of selected pluripotency genes and mature RPE markers. N = 3 biological repeats. Presented data are derived from RNA-seq analysis performed on hESCs and hESC-RPE. Please refer to Excel Table S1 for raw data. The accession number for the RNAseq data reported here is GEO: GSE127352.

(E) Immunoblot (western blot) analysis of MERTK using total protein extracts from wild-type and *MERTK* mutant RPE.

(F) Analysis of three MERTK immunoblots using ImageJ showed a decrease in MERTK expression in EX14-RPE, and no MERTK expression in EX2-RPE and MERTK-RPE. Data are represented as means ± SD. N = 3 biological repeats.

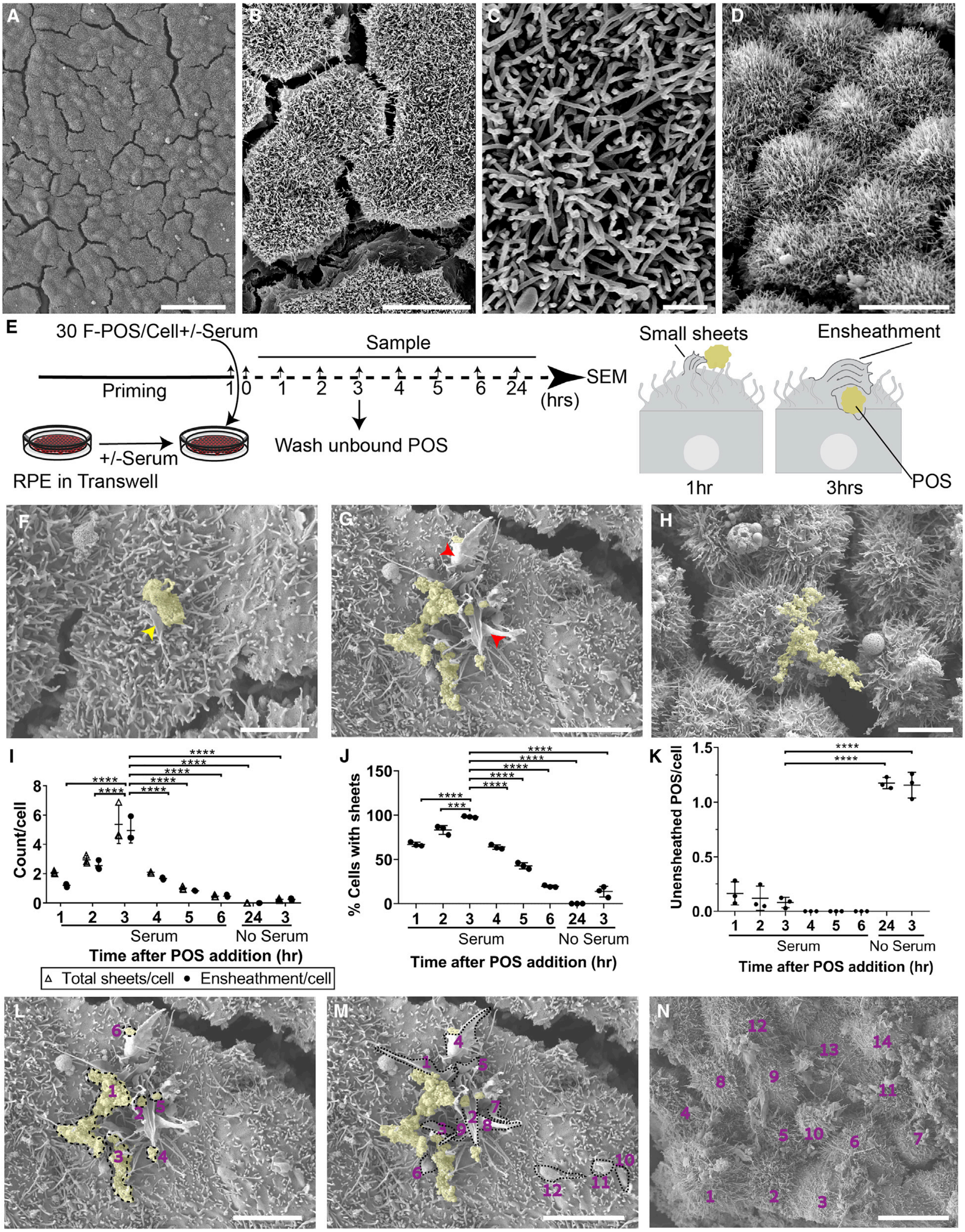

(legend on next page)

MERTK ligands are sufficient to trigger POS ensheathment, contrary to $\alpha V\beta 5$ Integrin receptor ligands.

MERTK Mutations in Human RPE Lead to Defects in POS Phagocytosis

The fact that GAS6 or PROS1 alone are sufficient to stimulate POS ensheathment suggested that their receptor, MERTK, is also involved in this process. Therefore, we hypothesized that the absence of *MERTK* might lead to defects in POS ensheathment. Two *MERTK* knockout hESC lines, namely EX2 (homozygous deletion from exon 2 onward) and EX14 (heterozygous deletion of exon 14 onward), were generated using CRISPR/Cas9 in hESCs. In addition, an iPSC line was derived from an RP38 patient harboring a genomic deletion in *MERTK* (Figures 5A and S4–S6). *MERTK* mutations are known to cause an RPE phagocytosis defect in animal models, leading to accumulation of POS in the IPM, and retinal degeneration and vision loss in RP 38 patients (Duncan et al., 2002). To validate this phenotype within our human RPE model, we analyzed F-POS binding and internalization by means of immunoblot in healthy versus *MERTK* mutant RPE. At first, we determined the kinetics of POS binding, internalization, and degradation in wild-type hESC-RPE in the presence of 30% serum, under ensheathment conditions (Figures 5C and 5D). At 3 h after F-POS addition, most of the POS were bound and partially internalized. When cells were challenged with POS for 6 h, more POS were bound and internalized. In addition, when unbound POS were washed off at 3 h, all the bound POS were internalized and partially degraded after 6 h, and fully degraded after 24 h. Internalization of POS by wild-type hESC-RPE was observed in the presence and absence of ensheathment, when treated with GAS6 or MFGE8, respectively (Figures 5E and 5F). RHO signal significantly decreased after 24 h compared with 3 h in GAS6-treated cells indicating efficient POS degradation in the presence of ensheathment, while the drop in RHO signal seen in MFGE8-treated cells at the same time point was not significant and showed a lot of variation. Thus, under ensheathment conditions wild-type hESC-RPE were able to bind, internalize and degrade POS. In contrast, all *MERTK* mutant RPE cells had minimal if any internalization at 3 or 6 h after POS addition (Figures 5G and 5H). By means of CRISPR/Cas9-mediated genomic engineering, we integrated wild-type *MERTK* sequence in the genomic *MERTK* locus in the patient iPSC line (Figure S7A). Recovery of MERTK expression in patient MERTK-RPE (Figures S7B–S7D) resulted in correction of the phagocytosis defect (Figures S7E and S7F). This confirms that the phagocytosis defect seen in patient MERTK-RPE is due to the identified genomic deletion in *MERTK*.

MERTK Mutations in Human RPE Abolish POS Ensheathment

To determine the role of MERTK during POS ensheathment, ultrastructural SEM analysis of F-POS ensheathment was evaluated in wild-type versus *MERTK* mutant RPE in the presence of 30% serum or 5 μg/mL GAS6 (Figure 6A). In the absence of functional MERTK, F-POS bound to RPE microvilli and were not ensheathed by the RPE (Figures 6B–6H). Taken together, the human RPE model shows that MERTK is not only critical for POS phagocytosis, but also for membrane remodeling occurring at the RPE-photoreceptor interface leading to ensheathment.

Figure 2. HESC-Derived RPE Recapitulate Dynamic POS Ensheathment *In Vitro*

(A–D) SEM of hESC-RPE without POS. Scale bars, 50 μm (A), 10 μm (B), 1 μm (C), and 10 μm (D).

(E) A schematic of the experiment. HESC-RPE cells were primed with 30% serum for 1 h before addition of F-POS or left untreated. After seeding F-POS, cells were incubated for 1, 2, or 3 h. After 3 h unbound POS were washed off with medium containing serum and cells were fixed at 4, 5, or 6 h. Samples without serum were incubated for 3 and 24 h before they were fixed and processed for SEM.

(F–H) SEM images of hESC-RPE with F-POS. F-POS are artificially colored yellow. Scale bar, 5 μm (F). Cells that were treated with POS and serum for 1 h show smaller and fewer sheets. Yellow arrow points to small sheets with POS. (G) Cells that were treated with POS and serum for 3 h show larger and numerous sheets. Red arrow points to the big sheets surrounding POS. See also Figures S1 and S2. (H) Cells that were treated with POS without serum for 3 h did not form sheets.

(I–K) Ten images containing around ten cells from each condition were analyzed. (I) The presence of serum and POS increases the number of total sheets per cell, and the number of sheets associated with POS. In the absence of serum very few sheets are observed. The number of sheets/cell is highest at 3 h after POS addition (pulse) and significantly decreases 3 h after POS pulse. Significance was calculated using two-way ANOVA comparing all samples to 3 h. (J) The percentage of cells that extend sheets in response to POS and serum increases significantly at 3 h and decreases again after 6 h. In contrast, cells that received POS without serum do not show ensheathment either at 3 h or at 24 h. (K) In the absence of serum most of the bound POS are unensheathed. Statistical significance for (J and K) was calculated using one-way ANOVA comparing all samples to 3 h. Data are represented as means ± SD. N = 3 biological repeats. ns > 0.05, $^*p < 0.05$, $^{**}p < 0.01$, $^{***}p < 0.001$, $^{****}p < 0.0001$.

(L–N) Examples for quantifying F-POS, sheets, and cells within a field of view. (L) Manual segmentation of F-POS. Six POS fragments were counted. Scale bar, 5 μm. (M) Manual segmentation of sheets. Twelve sheets were counted. Scale bar, 5 μm. (N) Manual quantification of the cells. Fourteen cells were counted. Scale bar, 10 μm.

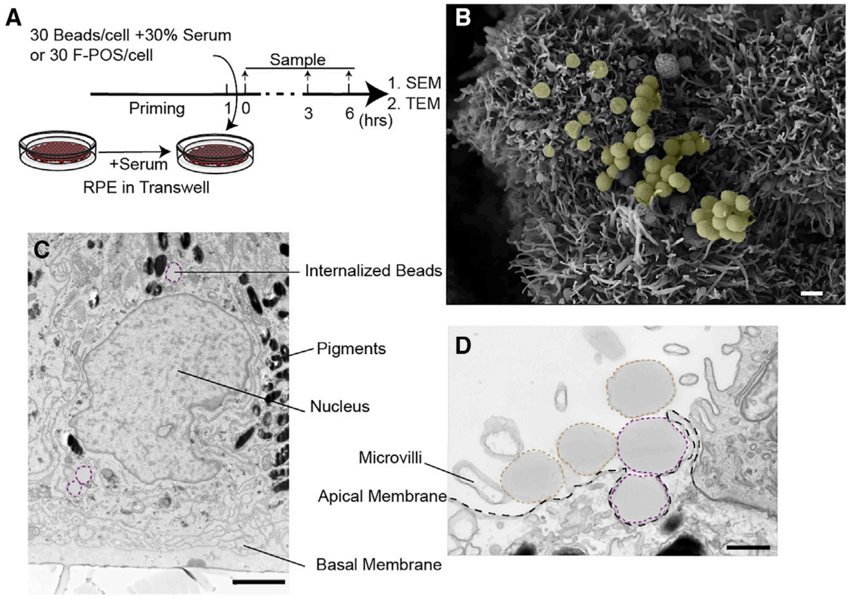

Figure 3. Ensheathment by Human RPE Is Specific for POS

(A) A schematic of the experiment. HESC-RPE cells were primed with 30% serum for 1 h before addition of F-POS or latex beads. Control cells were either left untreated or treated with beads without serum. Cells were incubated for 3 h at 37°C. All samples were washed after 3 h. Some samples were fixed and others were kept for 3 h or more to allow internalization of beads. Samples were analyzed with SEM or TEM.

(B) SEM image of RPE cells treated with beads (artificially colored yellow) for 3 h. Scale bar, 1 μm.

(C) TEM image at 6 h. Beads (dashed purple line) were observed inside the cells and close to the nuclei.

(D) TEM image at 3 h. Beads were observed either at the surface of the cell (dashed orange line), or getting internalized by the RPE (dashed purple line). In both SEM and TEM analyses, no sheets were observed around the beads.

Ensheathment Is Required for POS Fragmentation before Internalization

Upon treatment of wild-type hESC-RPE with MFGE8, uptake of already F-POS can occur in the absence of ensheathment. Thus, we hypothesized that ensheathment might be required to fragment POS before internalization. For this reason, we modified our POS isolation method to obtain whole full-sized unfragmented POS (W-POS) from photoreceptor cells (Figure S1A). Of note, two types of POS could be distinguished by SEM: round-oval and elongated (Figure 6J), which were RHO rich as seen by TEM (Figure 6K). By means of SEM analysis, we observed that upon addition of W-POS to wild-type hESC-RPE, in the presence of 30% serum, ensheathing membranes established contact with W-POS (Figure 6L), invaded the outer membrane enclosing the discs (Figure 6M) and finally fragmented it (Figure 6N). Next, we explored the dynamics of W-POS ensheathment and its fragmentation in wild-type hESC-RPE treated with 5 μg/mL MFGE8, 5 μg/mL GAS6, or 30% serum; and in *MERTK* mutant RPE, treated with 30% serum. Analysis of SEM images (Figure 6O) showed that around 80% of W-POS, in hESC-RPE treated with serum, and 60% of W-POS, in hESC-RPE treated with GAS6, were ensheathed by 3 h (Figures 6P and 6Q), and fragmented by RPE ensheathing membranes by 5 h after addition (Figures 6R and 6S). However, around 20% of the POS in *MERTK* mutant RPE and MFGE8-treated wild-type RPE, appeared fragmented without associated ensheathment at both 3 and 5 h, and displayed neither ensheathment at 3 h (Figures 6T and 6U), nor ensheathment-associated fragmentation by 5 h (Figures 6V and 6W). These results show that ensheathment is associated with POS fragmentation in human RPE, and that POS ensheathment and ensheathment-mediated fragmentation is defective in the absence of a functional MERTK.

Recovery of MERTK expression in patient MERTK-RPE (Figures S7A–S7D) rescued the pathologic phenotype and resulted in the formation of proper ensheathing membranes and subsequently fragmentation of W-POS (Figures S7G–S7K). This confirms that the ensheathment defect seen in patient MERTK-RPE is due to the identified genomic deletion in *MERTK*.

To monitor the time needed for W-POS fragmentation, we treated wild-type hESC-RPE and *MERTK* mutant EX2-RPE with AF488-labeled W-POS and 30% serum or 5 μg/mL MFGE8 and performed live imaging of the apical surface of RPE cells with an upright confocal microscope. To monitor actin changes on the surface of the RPE, we labeled the cells with SiR-actin in culture. Three hours after W-POS addition, fragmentation was observed in live hESC-RPE, but not EX2-RPE (Figure 7A; Video S1a) or MFGE8-treated hESC-RPE (Video S1b). When zooming in on particles that were still unfragmented we could observe live in culture W-POS fragmentation within 52 min by hESC-RPE. Quantification of the size (area) of POS in the fluorescence time-lapse images showed that they had a reduced size in hESC-RPE compared with EX2-RPE treated with serum or hESC-RPE treated with MFGE8 already at the start of imaging, 3 h after POS addition (0 min). The size of POS diminished further after 56 min in hESC-RPE treated with serum, but not in the other

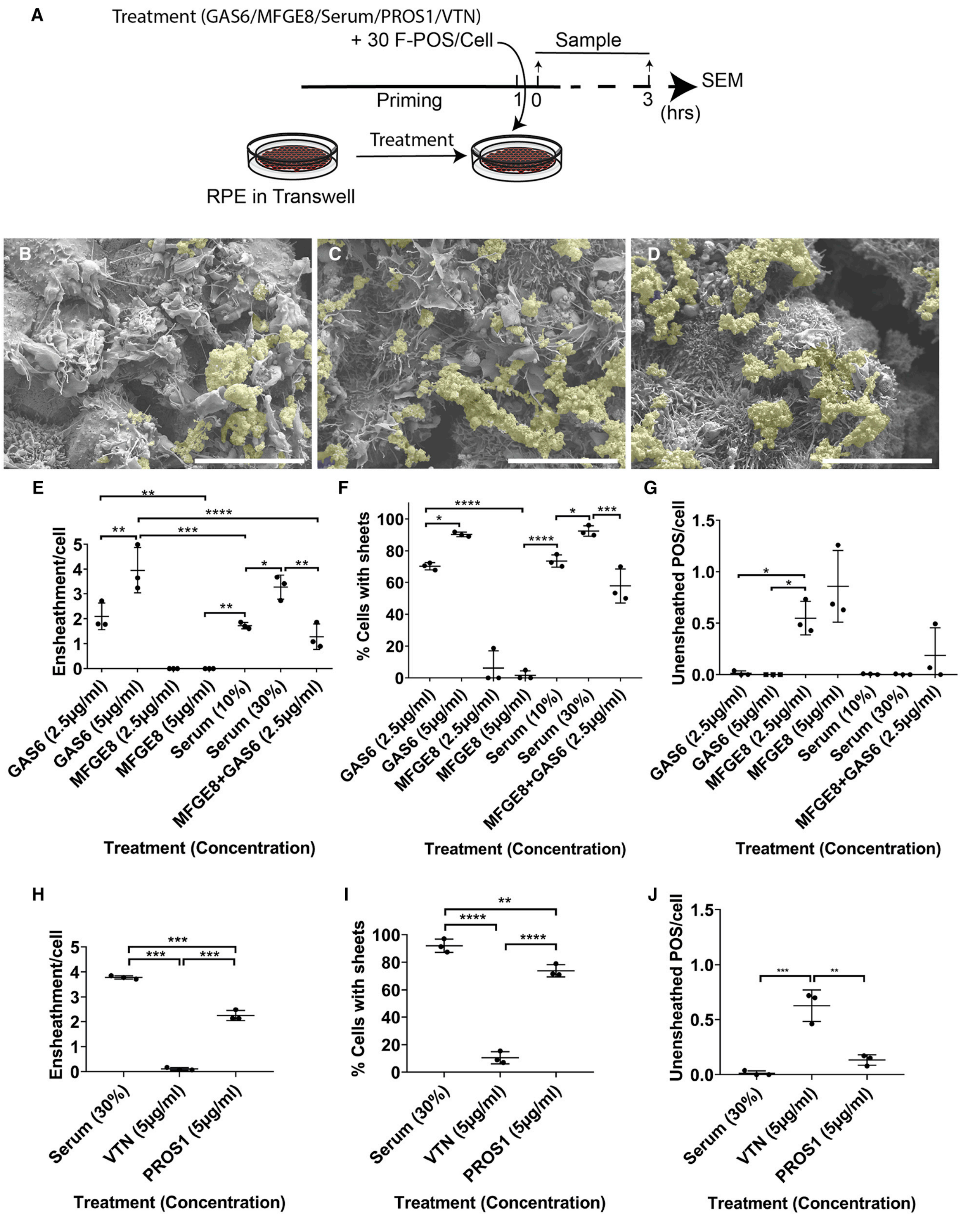

(legend on next page)

conditions (Figure 7B). To monitor uptake and co-localization of POS particles with lysosomes, we plated the cells in 96-well plates and performed live imaging with an inverted confocal microscope from the basal side of the cells. Cells were either labeled with SiR-actin or LysoTracker, which labels lysosomes. Co-localization of POS with lysosomes was observed in hESC-RPE, but not in EX2-RPE treated with 30% serum (Figures 7C and 7E; Videos S2 and S3). Interaction of actin-rich filaments with POS and internalization of smaller fragmented particles was observed in hESC-RPE, and lasted around 30 min (Figure 7D; Video S2). As expected, no internalization was observed in EX2-RPE, instead W-POS accumulated at the apical side of the cells (Figure 7F; Video S3). These results show that, under ensheathment conditions, fragmentation of W-POS occurs within approximately 50 min after binding, followed by internalization and co-localization with lysosomes within approximately 30 min. POS fragmentation and internalization were not observed under conditions where ensheathment is not observed, which indicates that ensheathment is required for POS fragmentation before internalization. Taken together, *MERTK* mutation in RPE causes a deficiency in POS fragmentation and phagocytosis, implicating both processes and their interconnection as pathologies that can contribute to vision loss in patients with RP38.

DISCUSSION

In this study, we established a human RPE-based model to perform mechanistic studies of POS ensheathment and phagocytosis by healthy and diseased RPE. Using this model we observed that MERTK ligands, GAS6 and PROS1, stimulate POS phagocytosis and ensheathment, and that loss of MERTK function leads to loss of both cellular processes. These findings were demonstrated in two CRISPR/Cas9-mediated *MERTK* knockout hESC-RPE and in RP38 patient iPSC-derived RPE. Rescue of MERTK expression, by CRISPR/Cas9-mediated integration of *MERTK* under the *CMV* promoter in the *MERTK* genomic region, led to regain of ensheathment-mediated fragmentation and phagocytosis of POS. This shows that the loss of ensheathment phenotype observed in patient cells is due to the genomic deletion identified in the *MERTK* coding sequence, and that ensheathment is MERTK dependent.

Although serum is not physiologically present in the IPM, both GAS6 and PROS1, which are also present in serum (Hall et al., 2005; Jiang et al., 2017), are expressed and secreted by the RPE in the IPM *in vivo* and stimulate POS phagocytosis (Hall et al., 2005; Karl et al., 2008). Phagocytosis ligands are often supplemented exogenously to boost POS phagocytosis *in vitro* (Law et al., 2015). Our hESC-RPE are differentiated and cultured in serum-free conditions, which is known to enhance endogenous GAS6 expression (Karl et al., 2008). In addition, our RNA sequencing data shows that both PROS1 and GAS6 expression are upregulated in hESC-RPE compared with hESCs. However, the levels of endogenously expressed GAS6 and PROS1 might not be sufficient, or require additional extrinsic signals for their secretion, to significantly induce ensheathment.

POS ensheathment *in vivo* occurs at the site of phagocytosis (Matsumoto et al., 1987). However, its role during phagocytosis is not clear. Here we show that, similar to POS phagocytosis, ensheathing RPE membranes get actively engaged in binding to the POS through receptor-mediated mechanism, leading to a more specific particle recognition. In contrast, 0.72-μm latex beads, which are internalized through caveolae-mediated non-specific endocytosis (Rejman et al., 2004) and independently of MERTK (Carr et al., 2009), did not engage with RPE ensheathing membranes. Addition of MFGE8 to wild-type RPE, which leads to αVβ5 integrin activation (Nandrot et al., 2007), increased both F-POS binding and internalization, which is in line with previous studies (Law et al., 2015). Surprisingly, MFGE8 did not stimulate POS ensheathment in wild-type RPE. Instead, POS landed passively on microvilli, and got internalized in the absence of ensheathment through a yet unidentified mechanism. In contrast, addition of GAS6, which is known to bind and activate MERTK (Law et al., 2015), triggered both POS ensheathment and internalization. Thus, MERTK activation drives ensheathment and phagocytosis, while αVβ5 integrin drives phagocytosis in the presence of an intact MERTK receptor. Consistently, we observed that serum failed to stimulate ensheathment or phagocytosis in *MERTK* mutant RPE even in the presence of an intact αVβ5 integrin receptor, which shows that activation of the latter alone is insufficient to induce both cellular mechanisms and indicates that activation of MERTK and ensheathment might be involved in a process upstream to

Figure 4. MERTK Ligands Rather Than αVβ5 Integrin Ligands Stimulate POS Ensheathment by Human RPE
(A) A schematic of the experiment.
(B–D) SEM image of cells treated with F-POS and 30% serum (B), 5 μg/mL GAS6 (C), or 5 μg/mL MFGE8 (D). Scale bar, 1 μm.
(E–J) Ten images containing around ten cells from each condition were analyzed. Data are represented as means ± SD. N = 3 biological repeats. (E–J) Quantification of the number of sheets associated with POS per cell, the percentage of cells presenting sheets, and the number of unensheathed POS per cell in the different conditions. Significance was calculated using one-way ANOVA test. ns > 0.05, *p < 0.05, ** p< 0.01, ***p < 0.001, ****p < 0.0001. Data are represented as means ± SD. N = 3 biological repeats. Related to Figure S3.

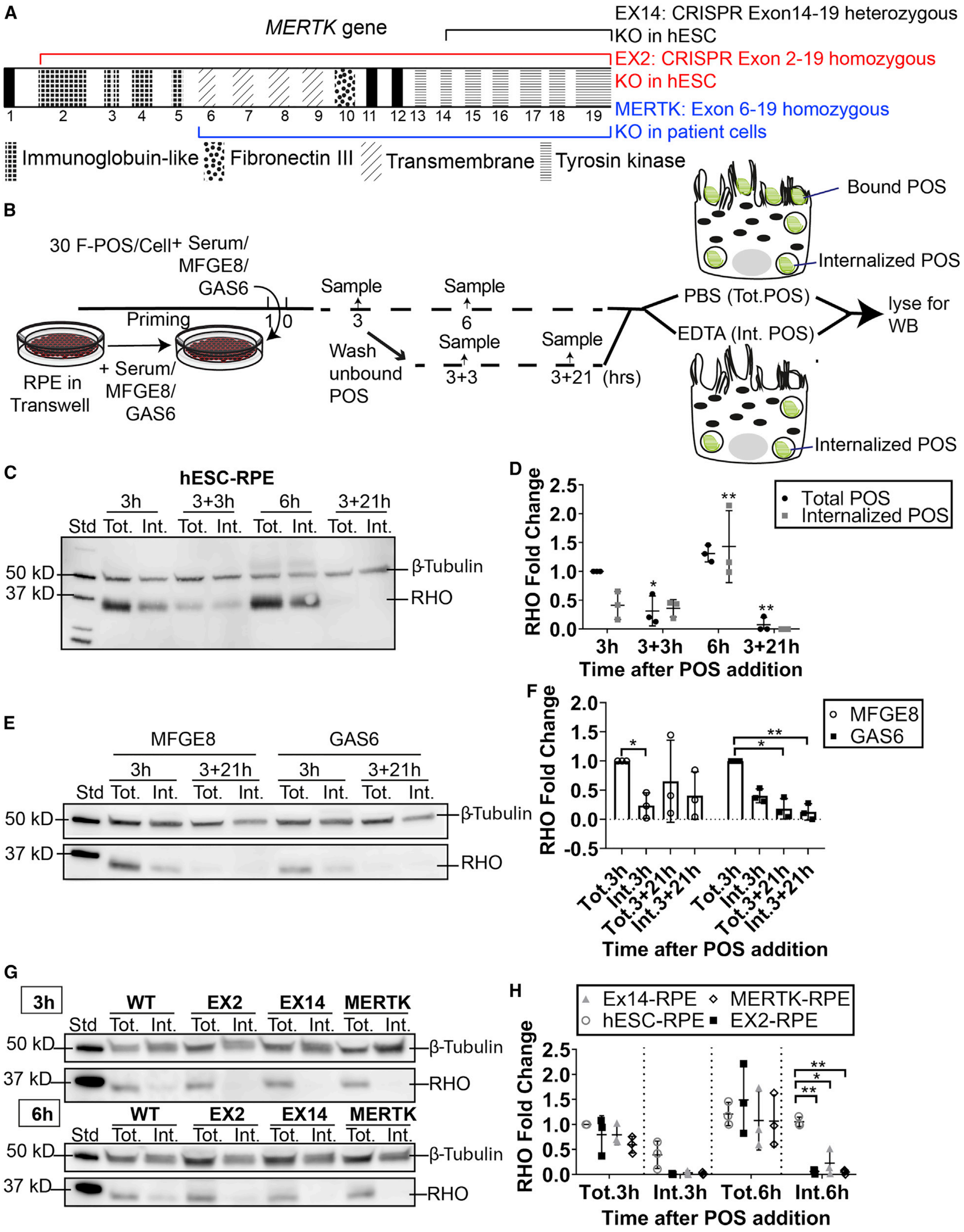

(legend on next page)

$\alpha V\beta 5$ integrin activation and POS internalization, such as POS fragmentation from the photoreceptors. To test this hypothesis, we isolated W-POS, both round-oval and elongated, which probably originate from rods and cones, respectively. We observed that RPE cells do not readily engulf W-POS, but rather fragment them using ensheathing membranes before internalization. The physiological reason for that might be that RPE are not able to ingest very big particles, such as W-POS (Irschick et al., 2004). W-POS ensheathment and ensheathment-mediated fragmentation by hESC-RPE occurred in the presence of 30% serum or 5 μg/mL GAS6, but not MFGE8, and was absent in *MERTK* mutant RPE cells. The presence of a small percentage of fragmented W-POS in the absence of ensheathment might be due to POS breaking during sample processing before addition to the cells (Parinot et al., 2014). These results provide a mechanistic evidence that POS ensheathment might be necessary to "bite-off" POS fragments as eatable-sized portions from the photoreceptors, and that MERTK activation is required for ensheathment-mediated fragmentation of POS before internalization.

Here, we present two POS models to study the interaction between POS and RPE. We observed that F-POS are more homogeneous in size, and are ensheathed, internalized and degraded faster than W-POS because of the obvious advantage that they do not have to be fragmented first by the RPE. Thus, F-POS are useful, when setting up a fluorescence-based screening assay where time and homogeneous POS size matter. On the other hand, W-POS are more physiological and useful to study basic mechanisms and answer disease-related questions. Ensheathment of W-POS occurs independently from the presence of the entire photoreceptor cell, which overcomes the need to have sophisticated RPE-photoreceptor cells co-culture techniques and increases the throughput of such assays. Thus, having both models at hand might facilitate the identification of new mechanisms and therapeutic targets.

Loss of ensheathment contributes to failure in retinal reattachment in pathologies with retinal detachment (Wickham et al., 2012). More recently, loss of ensheathment has been also shown in a canine model of Best vitelliform macular dystrophy (BVMD), leading to retinal detachment and degeneration, and subsequent loss of vision (Guziewicz et al., 2017). Based on our findings that POS ensheathment is disturbed in *MERTK* mutant RPE, the pathology of RP38 might follow a similar path to that of BVMD. It is quite likely that vision loss in RP38 patients is due to lack of POS ensheathment in preparation for their internalization. Thus, improving ensheathment might be a potential therapeutic strategy for these patients and others suffering from retinal diseases, where phagocytosis might be impaired, such as BVMD (Guziewicz et al., 2017), and age-related macular degeneration (Inana et al., 2018).

Taken together, our findings reveal exciting aspects of the molecular machinery controlling essential photoreceptor-RPE interactions, and present hPSC-RPE as a powerful

Figure 5. Mutations in the *MERTK* Gene Lead to Defects in POS Phagocytosis

(A) An illustration of the *MERTK* gene showing the different exons coding for the different protein domains of the receptor. Exons are labeled 1 to 19. The mutations in the three stem cell lines used in this study are also represented in the illustration: the iPSC cell line derived from an RP38 patient has a homozygous deletion spanning exon 6 to 19 (blue). CRISPR/Cas9-mediated partial deletion of exon 14 to 19 in hESCs is shown in black. CRISPR/Cas9-mediated deletion of exon 2 to 19 in hESCs is shown in red. See also Figures S4–S7 for *MERTK* mutations details, patient clinical observations, stem cell characterization, and rescue in isogenic iPSC-RPE control.

(B) A schematic of the three experiments in (C–H). All samples were treated with either PBS or EDTA before lysis to detect total versus internalized F-POS, respectively. Blots were probed for β-tubulin as a loading control, and RHO to indicate total POS (Tot.) and internalized POS (Int.). RHO signal was normalized to tubulin and then to the total POS signal in control condition to monitor the fold change in RHO signal. Data are represented as means ± SD. N = 3 biological repeats. Significance was calculated using two-way ANOVA test. ns > 0.05, *p < 0.05, **p < 0.01, ***p < 0.001, ****p < 0.0001.

(C) HESC-RPE were primed for 1 h with 30% serum. Next, cells were challenged with F-POS and incubated for 3 or 6 h at 37°C. Samples were lysed 3 or 6 h after POS addition. Other samples were washed at 3 h and kept for 3 h (3 + 3 h) or 21 h (3 + 21 h) before lysis to monitor POS degradation.

(D) Quantification of the immunoblot in (C). Total POS 3 h was used as control condition for normalization and statistical significance calculation.

(E) HESC-RPE were primed for 1 h with either GAS6 or MFGE8. Next, cells were challenged with POS and incubated for 3 h and either lysed or washed and kept for a further 21 h (3 + 21 h) before lysis to monitor POS degradation.

(F) Quantification of the immunoblot in (E). Total POS 3 h in each condition was used as control for normalization and statistical significance calculation. HESC-RPE bind, internalize, and degrade POS efficiently in the presence of GAS6 and serum. In contrast, in the presence of MFGE8 POS degradation is slowed down. See Figure S3 for fluorescence-based POS phagocytosis data.

(G) Wild-type and *MERTK* mutant RPE were primed for 1 h with 30% serum. Next, cells were challenged with POS and incubated for 3 or 6 h at 37°C before processing.

(H) Quantification of the immunoblot in (G). Total POS 3 h in hESC-RPE was used as control condition for normalization. For significance calculation all samples were compared with each other. *MERTK* mutant RPE show POS internalization defect.

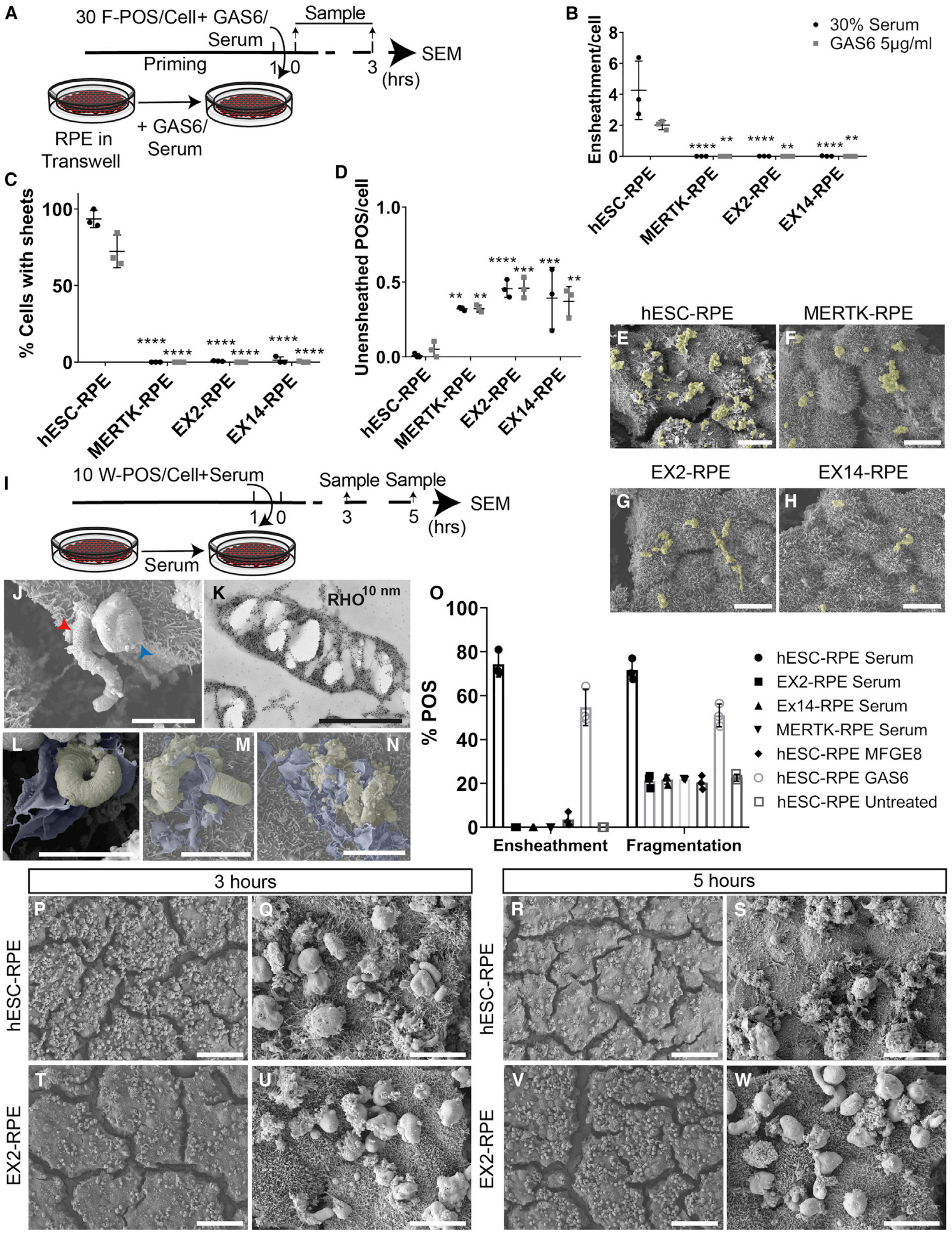

(legend on next page)

in vitro model to study POS ensheathment and renewal, which are essential for visual function. With our RPE models, a more detailed characterization of the molecular events leading to POS ensheathment and its pathology in retinal degenerative diseases can now be undertaken.

EXPERIMENTAL PROCEDURES

Stem Cell Lines and RPE Generation

RPE derived from H9 hESC cell line (hESC-RPE) were used as wild-type control. Fibroblasts were isolated from skin biopsies from RP38 patients and were reprogrammed using the non-integrating Sendai virus. RPE differentiated from patient iPSC is referred to as "MERTK-RPE" throughout the manuscript. *MERTK* gene editing with CRISPR/Cas9 was done in H9 hESCs. Two modified hESC lines were obtained EX2 and EX14. Differentiated RPE is referred to as "EX2-RPE" and "EX14-RPE" throughout the article. Detailed description of the generation of *MERTK* knockout cell lines, and patient iPSC line, and the characterization of the pluripotent cell lines is found in Supplemental Experimental Procedures under the section "Generation and characterization of *MERTK* mutant hPSC lines." An isogenic control for the patient iPSC line was generated as described in the "Generation of the isogenic control" section in Supplemental Experimental Procedures. RPE derived from isogenic iPSC are referred to as TSS-RPE in some parts of the manuscript.

All RPE cells were differentiated on Transwell filters as described previously (Zhu et al., 2013). RPE cells were passaged for expansion two times on Transwells before use. For some experiments and as indicated in the experimental schemes, RPE cells were passaged to 384- or 96-well plates and used within 13 days. Passaging details can be found in Supplemental Experimental Procedures.

POS Isolation

POS were isolated from porcine eyes, as described in Molday et al. (1987) and Parinot et al. (2014) with some modifications (Figure S1A). More details on POS isolation and fluorescence labeling can be found in Supplemental Experimental Procedures.

Phagocytosis Assays

For all experiments, cells were primed with the different treatments for 1 h before the addition of POS. F-POS particles were sonicated in 500 μL RPE medium, containing the different treatments (serum, MFGE8, GAS6, PROS1, VTN), for 5 s 10% power with Branson Digital Sonifier 450, before addition to the cells. POS were quantified using the Neubauer chamber combined with fluorescence microscopy imaging and cell profiler analysis. Around 30 F-POS/cell and 10 W-POS/cell were seeded on the cells. More details on phagocytosis assays variations can be found in Supplemental Experimental Procedures or experimental figure schemes and legends.

SEM

RPE cells cultured on Transwell filters were fixed with modified Karnovsky's solution, containing 2% glutaraldehyde plus 2% paraformaldehyde in 0.1 M phosphate buffer (pH 7.4) until processing. Processing and imaging details can be found in Supplemental Experimental Procedures.

Quantification and Statistical Analysis

Data and Statistical Analysis

Two-way ANOVA test was used to calculate the statistical significance of the immunoblot data. One-way ANOVA test was used to calculate statistical significance of fluorescence-based phagocytosis assay. SEM images were blinded during quantification. ANOVA test was used for SEM data analysis as indicated in the figure legends. The following formulas were used to analyze the SEM data:

$$\textit{Ensheathment per cell} = \frac{\textit{Total number of ensheathing membranes}}{\textit{Total number of cells}}$$

$$\textit{Unensheathed F}-\textit{POS per cell} = \frac{\textit{Total number of unensheathed POS}}{\textit{Total number of cells}}$$

Figure 6. *MERTK* Mutations in Human RPE Abolish POS Ensheathment

(A) A schematic of the experiment in (B–H).

(B–D) Ten images containing around ten cells from each condition were analyzed. Significance was calculated using one-way ANOVA test. ns > 0.05, *p < 0.05, **p < 0.01, ***p < 0.001, ****p < 0.0001. Data are represented as means ± SD. N = 3 biological repeats. In Figure S7 ensheathment rescue in isogenic control is shown. (B) Quantification of the number of sheets associated with POS per cell. (C) Quantification of the percentage of cells presenting sheets. (D) Quantification of the number of unensheathed F-POS per cell.

(E–H) SEM images of wild-type and *MERTK* mutant RPE treated with F-POS and 30% serum. Scale bar, 10 μm. (E) Wild-type hESC-RPE. (F) *MERTK* mutant MERTK-RPE. (G) *MERTK* mutant EX2-RPE. (H) *MERTK* mutant EX14-RPE.

(I) A schematic of the experiment in (J–W). Wild-type and *MERTK* mutant RPE (grown on transwells) were primed 1 h with 30% serum. Alternatively, wild-type RPE cells were primed with 5 μg/mL GAS6 or MFGE8 for 1 h. Next, cells were challenged with W-POS and incubated for 3 and 5 h at 37°C. Samples were next processed for SEM.

(J) Two types of W-POS particles were observed by SEM elongated (red arrow) and round-oval (blue arrow) outer segments. Scale bar, 5 μm.

(K) Whole POS particles are RHO rich. Scale bar, 3 μm.

(L–N) SEM images. Stages of POS binding, ensheathment, and ensheathment-mediated fragmentation. Scale bar, 5 μm. (L) Upon contact between hESC-RPE and W-POS in the presence of 30% serum, RPE extends its membrane sheets around the particle. (M) Next, RPE sheets invade POS and fragment them. (N) POS are completely fragmented before they are internalized.

(O) Analysis of W-POS ensheathment in SEM images at 3 h and fragmentation by 5 h in the presence of 30% serum. Data are represented as means ± SD. N = 3 biological repeats.

(P–W) Representative SEM images of the analysis shown in (O). Scale bars, 50 μm (P, R, T, and V). Zoomed-in SEM images of (Q), (S), (U), and (W). Scale bar, 10 μm. W-POS were ensheathed at 3 h (P and Q) and fragmented after 5 h by hESC-RPE treated with 30% serum (R and S), while EX2-RPE showed neither POS ensheathment at 3 h (T and U), nor ensheathment-mediated fragmentation at 5 h (V and W).

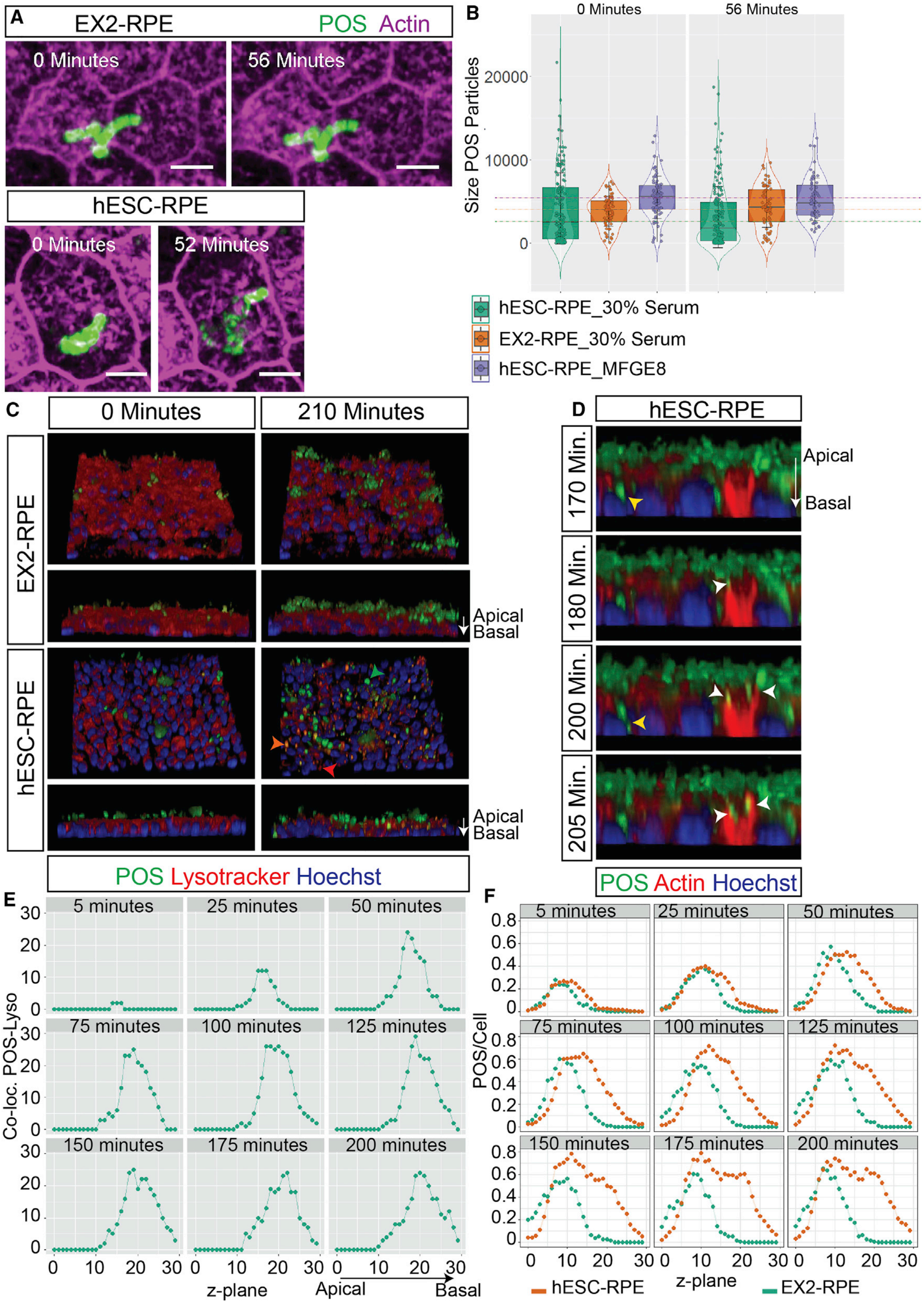

(legend on next page)

$$\% \ cells \ with \ sheets = \frac{Number \ of \ cells \ presenting \ sheets}{Total \ number \ of \ cells}$$

$$\% \ ensheathment \ of \ w-POS \ at \ 3 \ hrs = \frac{Number \ of \ ensheathed \ POS}{Total \ number \ of \ POS}$$

$$\% \ fragmentation \ of \ w-POS \ at \ 5 \ hrs = \frac{Number \ of \ fragmented \ POS}{Total \ number \ of \ POS}$$

Reagent sources and catalog numbers and further methods can be found in Supplemental Experimental Procedures in the "Key resources table."

Study Approval

Permission to work with hESCs was granted by the Robert Koch Institute, Berlin, Germany (license number AZ 3.04.02/0103-A01). The fibroblasts were isolated under full patient consent and approved by Columbia University under IRB protocol number AAAR0284. All procedures were in accordance with the Declaration of Helsinki.

SUPPLEMENTAL INFORMATION

Supplemental Information can be found online at https://doi.org/10.1016/j.stemcr.2020.02.004.

AUTHORS CONTRIBUTIONS

Conceptualization, S.A.; Investigation, S.A., S.S., T.K., and K.V.; Patient iPSC Derivation, S.H.T.; Support in iPSC Reprogramming and Genomic Engineering, S.K. and K.N.; Writing – Original Draft, S.A.; Writing – Review & Editing, all authors; Funding Acquisition, S.A., E.M.T., M.O.K., and M.A.

ACKNOWLEDGMENTS

We acknowledge the Federal Ministry of Education and Research (BMBF) for funding this project under the VIP+ program (project name and number: Cleansight, 03VP01220). We appreciate the support in the cell culture from the rest of the Cleansight team. We acknowledge Ruben Jauregui for clinical imaging. We appreciate the support from the core facilities at TUD-CRTD, which are funded by the European Fund for Regional Development (EFRE) and the State of Saxony, including sequencing facility and the light and electron microscopy facilities. We also appreciate the support from the LMF facility, the technology development studio (TDS), and the sequencing and genotyping facility in MPI-CBG.

Received: April 23, 2019
Revised: February 6, 2020
Accepted: February 10, 2020
Published: March 10, 2020

REFERENCES

Besharse, J.C., Hollyfield, J.G., and Rayborn, M.E. (1977). Photoreceptor outer segments: accelerated membrane renewal in rods after exposure to light. Science *196*, 536–538.

Bok, D., and Hall, M.O. (1971). The role of the pigment epithelium in the etiology of inherited retinal dystrophy in the rat. J. Cell Biol. *49*, 664–682.

Bulloj, A., Duan, W., and Finnemann, S.C. (2013). PI 3-kinase independent role for AKT in F-actin regulation during outer segment phagocytosis by RPE cells. Exp. Eye Res. *113*, 9–18.

Carr, A.-J., Vugler, A., Lawrence, J., Chen, L.L., Ahmado, A., Chen, F.K., Semo, M., Gias, C., da Cruz, L., Moore, H.D., et al. (2009).

Figure 7. Quantitative Live Imaging Analysis Shows that *MERTK* Mutant Human RPE Fail to Fragment and Internalize POS

(A) Snapshots from time-lapse live fluorescence imaging performed with LSM 880 upright microscope using the Airyscan feature and a 40× dipping objective. The complete time-lapse videos can be found in Videos S1a and S1b. HESC-RPE and EX2-RPE (grown on transwells) were primed with 30% serum or 5 μg/mL MFGE8 for 1 h, and were challenged with AF488-labeled W-POS for 2.5 h at 37°C with 5% CO_2 before imaging. Membranes, containing the RPE monolayer, were cut out from the Transwell insert and placed with the apical side of the cells facing the dipping objective in a 35-mm cell culture dish with 2 mL medium containing SiR-actin dye and 30% serum. (A) Many of the whole POS particles that were still seen at the start of imaging (0 min) in hESC-RPE got fragmented within around 52 min. In contrast, whole POS particles in EX2-RPE did not get fragmented within the same or longer time frame (56 min). Scale bar, 10 μm.

(B) Box-violin plot of the live imaging performed in (A) shows distribution of the POS particle size under different conditions at the start of the imaging (0 min) and at the end (56 min).

(C and D) Snapshots from time-lapse live fluorescence imaging performed with an inverted Leica confocal microscope (SP5-mp) on hESC-RPE and EX2-RPE plated in 96-well plates, and treated with 30% serum and SiR-actin (D) or LysoTracker (C), to monitor POS internalization or co-localization with lysosomes, respectively. The complete time lapse can be found in Video S2 (EX2-RPE) and Video S3 (hESC-RPE). Following priming with 30% serum W-POS were added to the cells and imaging started 25 min thereafter without a washing step in between. (C) In hESC-RPE, W-POS (green arrow) were fragmented into smaller pieces and co-localized with lysosomes (orange arrow). In contrast, W-POS remained intact in EX2-RPE and did not co-localize with lysosomes. (D) Actin labeling showed many internalized POS fragments in hESC-RPE (yellow arrow). Some POS fragments were captured during the process of internalization, which takes around 30 min (white arrow).

(E) Quantification of the number of POS that co-localize with lysosomes at different time points during the time lapse and in different z-planes, where (0) refers to the apical surface of the RPE and (30) refers to the basal. The number of co-localized POS increased with time and shifted toward the basal side of the RPE.

(F) The number of POS/cell in hESC-RPE over the different z-planes and time points during the time lapse showed a shift in the peak of POS/cell count toward basal z planes with time, reflecting internalization. This shift was not detected in EX2-RPE.

Molecular characterization and functional analysis of phagocytosis by human embryonic stem cell-derived RPE cells using a novel human retinal assay. Mol. Vis. *15*, 283–295.

Duncan, J.L., LaVail, M.M., Yasumura, D., Matthes, M.T., Yang, H., Trautmann, N., Chappelow, A.V., Feng, W., Earp, H.S., Matsushima, G.K., et al. (2002). An RCS-like retinal dystrophy phenotype in mer knockout mice. Invest. Ophthalmol. Vis. Sci. *44*, 826–838.

Feng, W., Yasumura, D., Matthes, M.T., LaVail, M.M., and Vollrath, D. (2002). Mertk triggers uptake of photoreceptor outer segments during phagocytosis by cultured retinal pigment epithelial cells. J. Biol. Chem. *277*, 17016–17022.

Finnemann, S.C., and Silverstein, R.L. (2001). Differential roles of CD36 and alphavbeta5 integrin in photoreceptor phagocytosis by the retinal pigment epithelium. J. Exp. Med. *194*, 1289–1298.

Fisher, S.K., and Lindberg, K.A. (1975). Intercellular junctions in the early human embryonic retina. J. Ultrastruct. Res. *51*, 69–78.

Guziewicz, K.E., Sinha, D., Gómez, N.M., Zorych, K., Dutrow, E.V., Dhingra, A., Mullins, R.F., Stone, E.M., Gamm, D.M., Boesze-Battaglia, K., et al. (2017). Bestrophinopathy: an RPE-photoreceptor interface disease. Prog. Retin. Eye Res. *58*, 70–88.

Hall, M.O., Prieto, A.L., Obin, M.S., Abrams, T.A., Burgess, B.L., Heeb, M.J., and Agnew, B.J. (2001). Outer segment phagocytosis by cultured retinal pigment epithelial cells requires Gas6. Exp. Eye Res. *73*, 509–520.

Hall, M.O., Obin, M.S., Heeb, M.J., Burgess, B.L., and Abrams, T.A. (2005). Both protein S and Gas6 stimulate outer segment phagocytosis by cultured rat retinal pigment epithelial cells. Exp. Eye Res. *81*, 581–591.

Inana, G., Murat, C., An, W., Yao, X., Harris, I.R., and Cao, J. (2018). RPE phagocytic function declines in age-related macular degeneration and is rescued by human umbilical tissue derived cells. J. Transl. Med. *16*, 63.

Irschick, E.U., Sgonc, R., Böck, G., Wolf, H., Fuchs, D., Nussbaumer, W., Göttinger, W., and Huemer, H.P. (2004). Retinal pigment epithelial phagocytosis and metabolism differ from those of macrophages. Ophthalmic Res. *36*, 200–210.

Jiang, L., Liu, C.Y., Yang, Q.F., Wang, P., and Zhang, W. (2017). Plasma level of growth arrest-specific 6 (GAS6) protein and genetic variations in the GAS6 gene in patients with acute coronary syndrome. Am. J. Clin. Pathol. *6*, 738–743.

Karl, M.O., Kroeger, W., Wimmers, S., Milenkovic, V.M., Valtink, M., Engelmann, K., and Strauss, O. (2008). Endogenous Gas6 and Ca^{2+}-channel activation modulate phagocytosis by retinal pigment epithelium. Cell. Signal. *20*, 1159–1168.

LaVail, M.M. (1976). Rod outer segment disc shedding in relation to cyclic lighting. Exp. Eye Res. *23*, 277–280.

Law, A.L., Parinot, C., Chatagnon, J., Gravez, B., Sahel, J.A., Bhattacharya, S.S., and Nandrot, E.F. (2015). Cleavage of mer tyrosine kinase (MerTK) from the cell surface contributes to the regulation of retinal phagocytosis. J. Biol. Chem. *290*, 4941–4952.

Mao, Y., and Finnemann, S.C. (2012). Essential diurnal Rac1 activation during retinal phagocytosis requires α v β 5 integrin but not tyrosine kinases focal adhesion kinase or Mer tyrosine kinase. Mol. Biol. Cell *23*, 1104–1114.

Matsumoto, B., Defoe, D.M., and Besharse, J.C. (1987). Membrane turnover in rod photoreceptors: ensheathment and phagocytosis of outer segment distal tips by pseudopodia of the retinal pigment epithelium. Proc. R. Soc. Lond. B Biol. Sci. *230*, 339–354.

Molday, R., Hicks, D., and Molday, L. (1987). Peripherin. A rim-specific membrane protein of rod outer segment discs. Invest. Ophthalmol. Vis. Sci. *28*, 50–61.

Nandrot, E.F., Kim, Y., Brodie, S.E., Huang, X., Sheppard, D., and Finnemann, S.C. (2004). Loss of synchronized retinal phagocytosis and age-related blindness in mice lacking alphavbeta5 integrin. J. Exp. Med. *200*, 1539–1545.

Nandrot, E.F., Anand, M., Almeida, D., Atabai, K., Sheppard, D., and Finnemann, S.C. (2007). Essential role for MFG-E8 as ligand for alphavbeta5 integrin in diurnal retinal phagocytosis. Proc. Natl. Acad. Sci. U S A *104*, 12005–12010.

Parinot, C., and Nandrot, E.F. (2016). A comprehensive review of mutations in the MERTK proto-oncogene. Adv. Exp. Med. Biol., 259–265. https://doi.org/10.1007/978-3-319-17121-0_35.

Parinot, C., Rieu, Q., Chatagnon, J., Finnemann, S.C., and Nandrot, E.F (2014). Large-scale purification of porcine or bovine photoreceptor outer segments for phagocytosis assays on retinal pigment epithelial cells. J. Vis. Exp., 1–8. https://doi.org/10.3791/52100.

Ramsden, C.M., Nommiste, B., R Lane, A., Carr, A.F., Powner, M.B., J K Smart, M., Chen, L.L., Muthiah, M.N., Webster, A.R., Moore, A.T., et al. (2017). Rescue of the MERTK phagocytic defect in a human iPSC disease model using translational read-through inducing drugs. Sci. Rep. *7*, 51.

Rejman, J., Oberle, V., Zuhorn, I.S., and Hoekstra, D. (2004). Size-dependent internalization of particles via the pathways of clathrin- and caveolae-mediated endocytosis. Biochem. J. *377*, 159–169.

Shelby, S.J., Colwill, K., Dhe-Paganon, S., Pawson, T., and Thompson, D.A. (2013). MERTK interactions with SH2-domain proteins in the retinal pigment epithelium. PLoS One *8*, 1–14.

Steinberg, R.H., Wood, I., and Hogan, M.J. (1977). Pigment epithelial ensheathment and phagocytosis of extrafoveal cones in human retina. Philos. Trans. R. Soc. Lond. B Biol. Sci. *277*, 459–471.

Strauss, O. (2005). The retinal pigment epithelium in visual function. Physiol. Rev. *85*, 845–881.

Wickham, L., Lewis, G.P., Charteris, D.G., and Fisher, S.K. (2012). Cellular effects of detachment and reattachment on the neural retina and the retinal pigment epithelium. In Retina, Fifth Edition (Elsevier), pp. 605–617. https://doi.org/10.1016/B978-1-4557-0737-9.00029-1.

Zhu, Y., Carido, M., Meinhardt, A., Kurth, T., Karl, M.O., Ader, M., and Tanaka, E.M. (2013). Three-dimensional neuroepithelial culture from human embryonic stem cells and its use for quantitative conversion to retinal pigment epithelium. PLoS One *8*, e54552.

Stem Cell Reports, Volume *14*

Supplemental Information

MERTK-Dependent Ensheathment of Photoreceptor Outer Segments by Human Pluripotent Stem Cell-Derived Retinal Pigment Epithelium

Seba Almedawar, Katerina Vafia, Sven Schreiter, Katrin Neumann, Shahryar Khattak, Thomas Kurth, Marius Ader, Mike O. Karl, Stephen H. Tsang, and Elly M. Tanaka

Supplemental data items: Figures and Legends

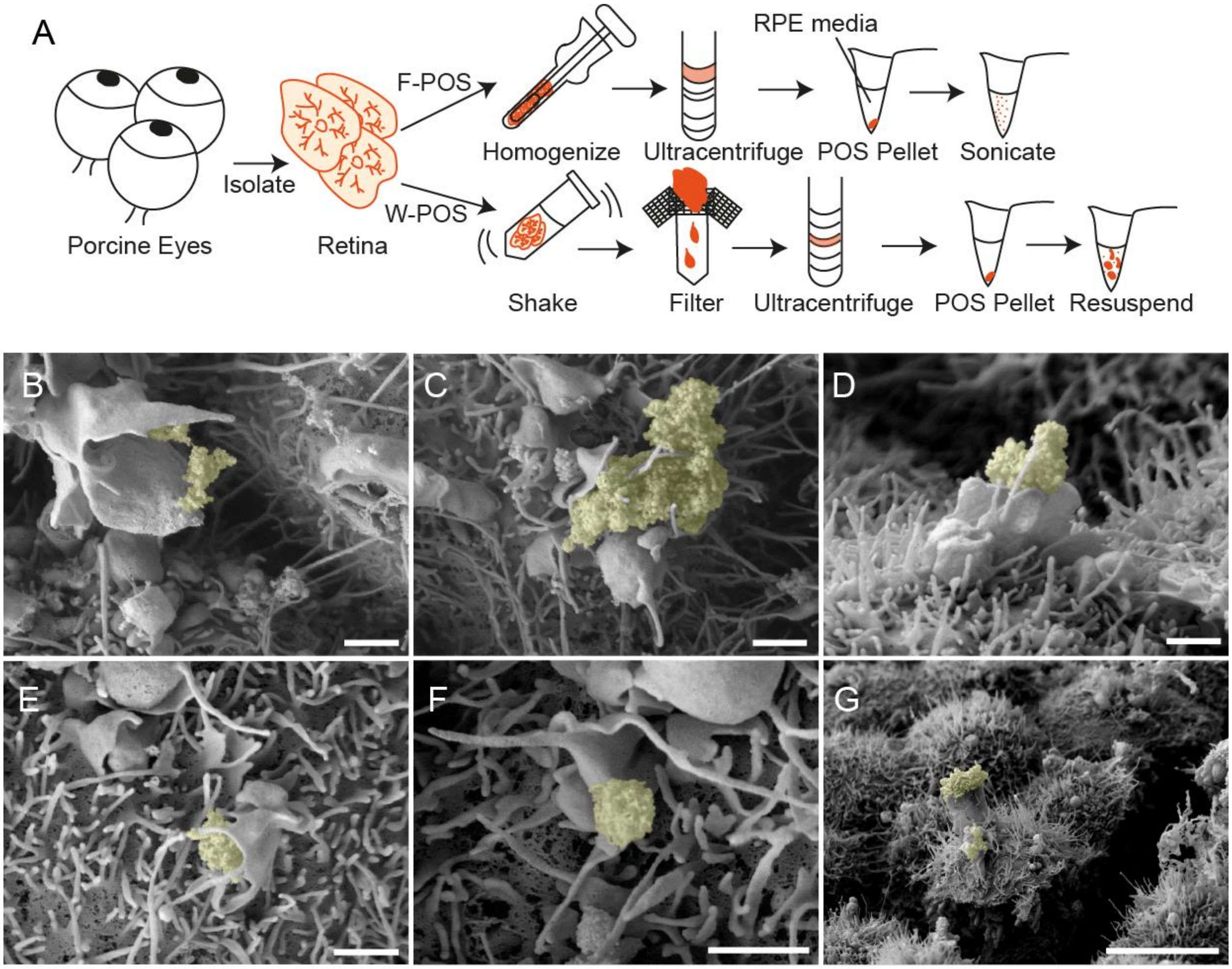

Figure S1. HESC-derived RPE recapitulate dynamic POS ensheathment in vitro. Related to Figure 2. (A) POS isolation scheme. Retinal tissue was isolated from porcine eyes and homogenized with a dounce glass tissue homogenizer. The homogenized retinal tissue was split in 6 tubes containing a sucrose gradient of 27%, 33%, 41%, 50% and 60%, and subjected to ultracentrifugation. The orange band was collected, centrifuged and the pellets were resuspended with POS storage solution and stored at -80ºC. For phagocytosis experiments, POS pellets were resuspended with RPE media and sonicated before addition to the cells. POS obtained using this method are around 0.5-1 µM in size and are referred to as fragmented POS (F-POS) throughout the manuscript. To obtain whole full-sized POS particles, isolated porcine retinas were shaken thoroughly in homogenization buffer, filtered through double layers of gauze, split in sucrose gradient tubes as before and subjected to ultracentrifugation. The faint orange band was collected from each gradient, and centrifuged. The pellets were resuspended with POS storage solution and stored at -80ºC. For phagocytosis experiments POS pellets were resuspended with RPE media and added to the cells without a sonication step. POS obtained using this method are 5-10 µM in size and are referred to as whole POS (W-POS) throughout the manuscript. (B-G) En face SEM images of hESC-RPE cells challenged with F-POS (yellow artificially-colored) shows the different forms of POS ensheathment seen after treatment with stimulants of POS phagocytosis such as serum. Scale bars: B-F: 1 µm, G: 10 µm.

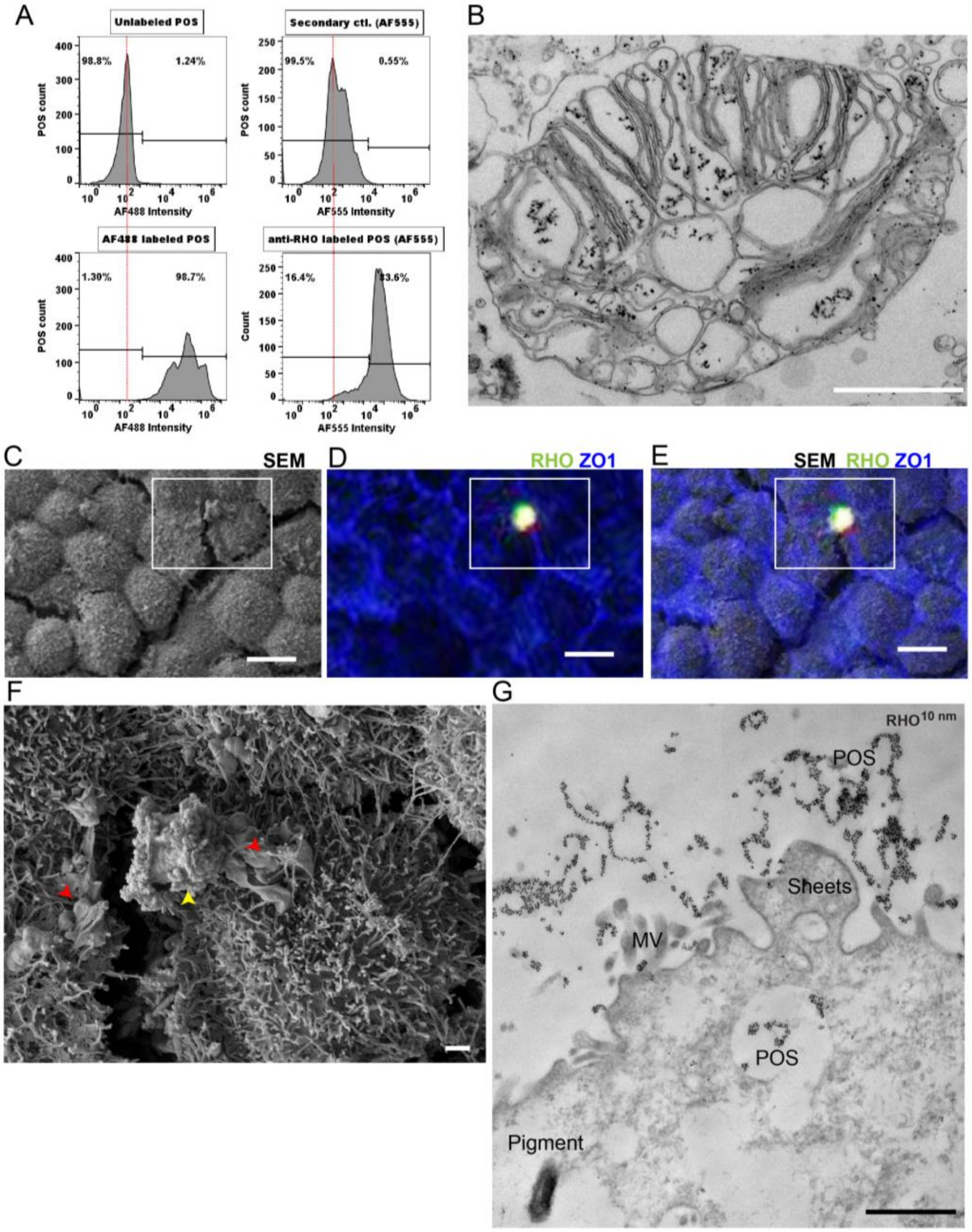

Figure S2. POS quality validation. Related to Figure 2. (A) FACS sorting of POS particles that were either unlabeled, labeled with Alexa Fluor 488 dye (AF488), labeled with Rhodopsin (RHO) antibody followed by secondary (AF555) or labeled with the secondary only (AF555) as a control. The gate of the AF488 labeled POS was set according to the unlabeled POS, while the one for RHO labeled POS was set according to the secondary control. FACS data showed that 98,7% of the POS were labeled with AF488, and 83,6% of the POS were RHO positive. (B) TEM analysis of isolated porcine POS. Membrane discs could still be observed. Scale bar 1 µm. (C) SEM of cells treated with F-POS and serum after immunolabeling with RHO. (D) Fluorescence image of the same area shown in C. Cells were immunolabeled with antibodies against ZO1 (blue) and POS (green) prior to critical point drying and sputter coating for SEM. (E) Overlay of SEM in (C) and fluorescence image in (D) shows that RHO positive POS seeded on cells co-localized with a POS particle surrounded by sheets as seen with SEM. Scale bar 10µm. (F) Higher magnification of C showed that sheets (red arrow) and not microvilli interact with RHO positive particles (yellow arrow). Scale bar 1 µm. (G) TEM analysis of F-POS seeded on cells. POS particles were labeled with anti-RHO and protein A 10 nm gold on ultrathin Lowicryl resin sections. RHO-positive POS were seen on the surface interacting with microvilli (MV) and membrane sheets or internalized within phagosomes. Scale bar 1 µm.

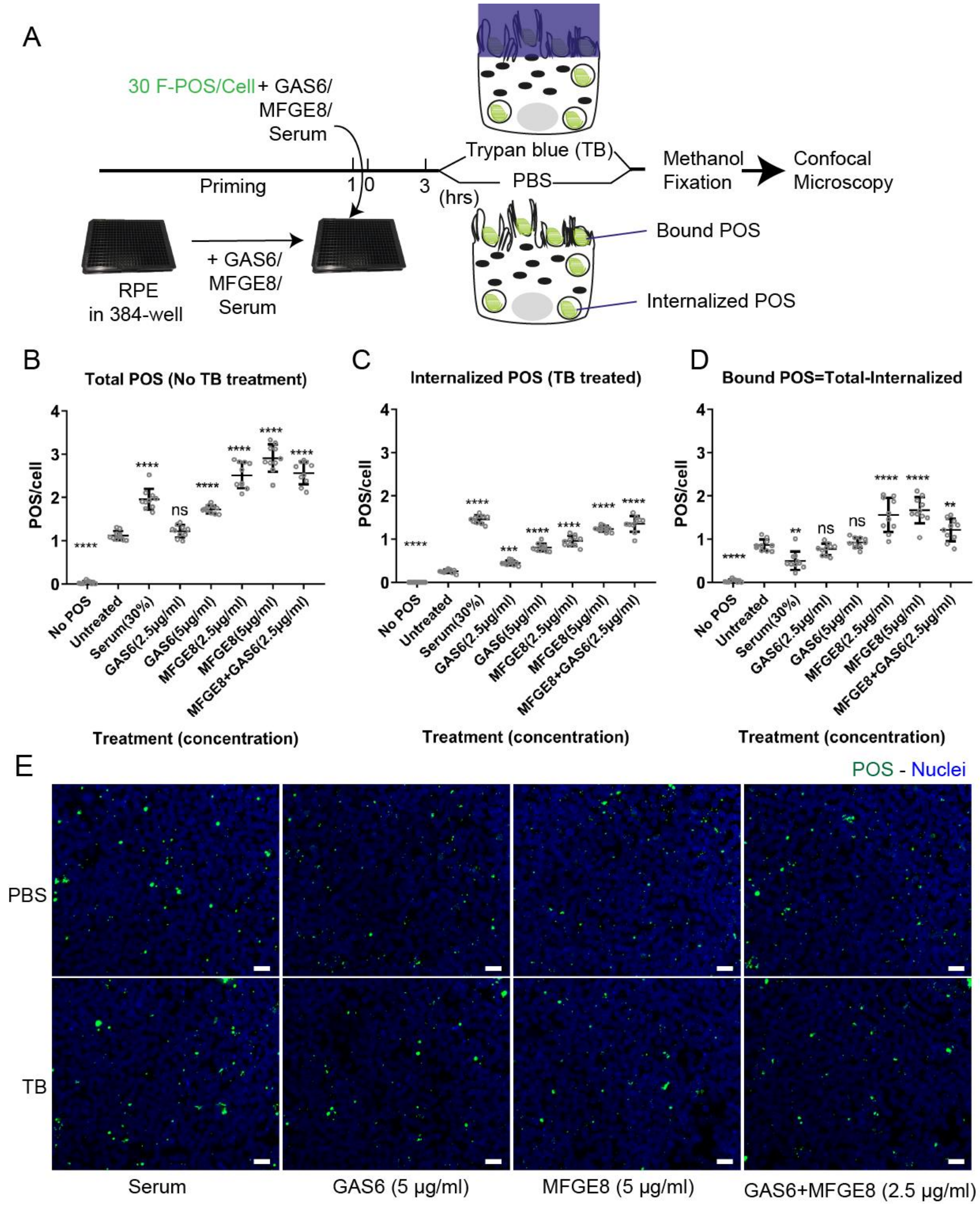

Figure S3. MFGE8 and GAS6 stimulate POS phagocytosis in human RPE. Related to Figure 5.
(A) A schematic of the experiment. HESC-RPE cells (grown on 384 well plates) were primed for 1 hour with either 30% serum, MFGE8, GAS6, or MFGE8 and GAS6 together at different concentrations. Next, cells were challenged with FITC labeled F-POS and incubated for 3 hours at 37ºC. At 3 hours, wells were either treated with 0.4% Trypan Blue (TB) to quench external POS signal or PBS. Samples were then washed and fixed with methanol. Finally, imaging was done using a confocal microscope. (B-D) The median between 6 fields and the mean of the medians of the different repeats was calculated. Data are represented as the mean ± SD. n=10 technical repeats. Significance was calculated using one way ANOVA test. All treatments were compared to untreated. ns >0.05, *<0.05, ** <0.01, ***<0.001, ****<0.0001. (B) Quantification of the number of POS/cell in wells that were not treated with TB to measure total POS (bound and internalized). (C) Quantification of the number of POS/cell in wells that were treated with TB to measure internalized POS. (D) Quantification of the number of bound POS/cell. Serum, GAS6 and MFGE8 increased total POS/cell count. MFGE8 increases POS binding and internalization, while serum and GAS6 increase mainly internalization. (E) Representative fluorescence images of the different conditions. Scale bar: 20 µm.

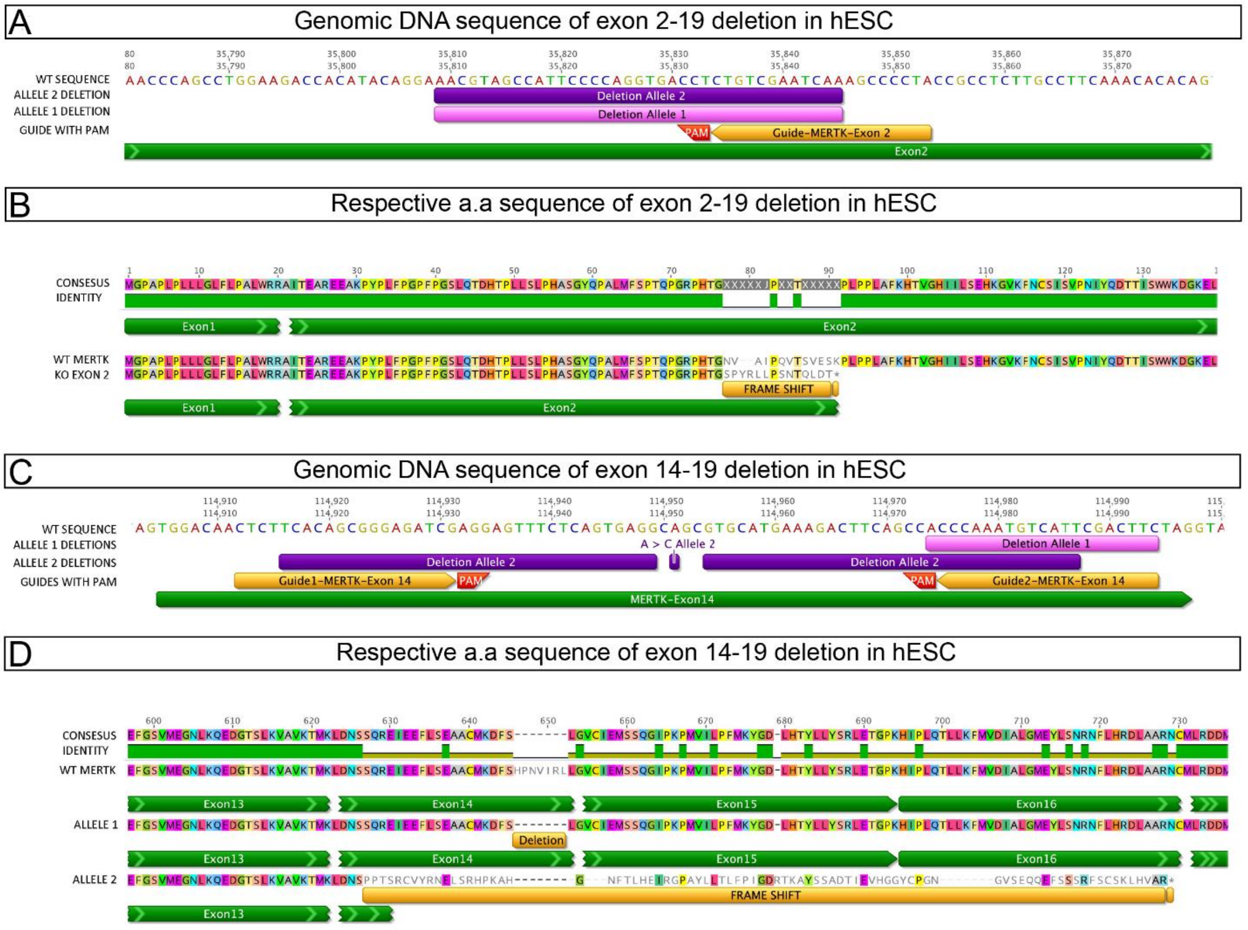

Figure S4. DNA and predicted amino acid sequence of *MERTK* in CRISPR/Cas9 genetically engineered hESC. Related to Figure 5. (A) *MERTK* exon 2 DNA sequence illustrating the deleted region in hESC based on allele specific sequencing. Top row shows the wild-type *MERTK* DNA sequence of exon 2 as shown by green arrow (bottom). The deletions location in the first and second MERTK alleles are marked in purple and pink. Yellow lines indicate the guides, while red boxes represent the respective PAMs for each guide. (B) Predicted amino acid sequence following genomic deletion in exon 2. Yellow rectangles represent the frameshift resulting from the nucleotide deletions in both alleles, while * depicts a premature translational STOP. (C) *MERTK* exon 14 DNA sequence illustrating the deleted region in hESC based on allele specific sequencing. Top row shows the wild-type *MERTK* DNA sequence of exon 14 as shown by green arrow (bottom). The deletions location in the first and second MERTK alleles are marked in pink and purple. Yellow lines indicate the guides, while red boxes represent the respective PAMs for each guide. (D) Amino Acid Sequence alignment of both MERTK alleles in EX14. Predicted amino acid sequence following genomic deletion in exon 14. Yellow rectangles represent the predicted deletion in allele 1 of exon 14 and the frameshift resulting from the nucleotide deletions in allele 2. * depicts a premature translational STOP.

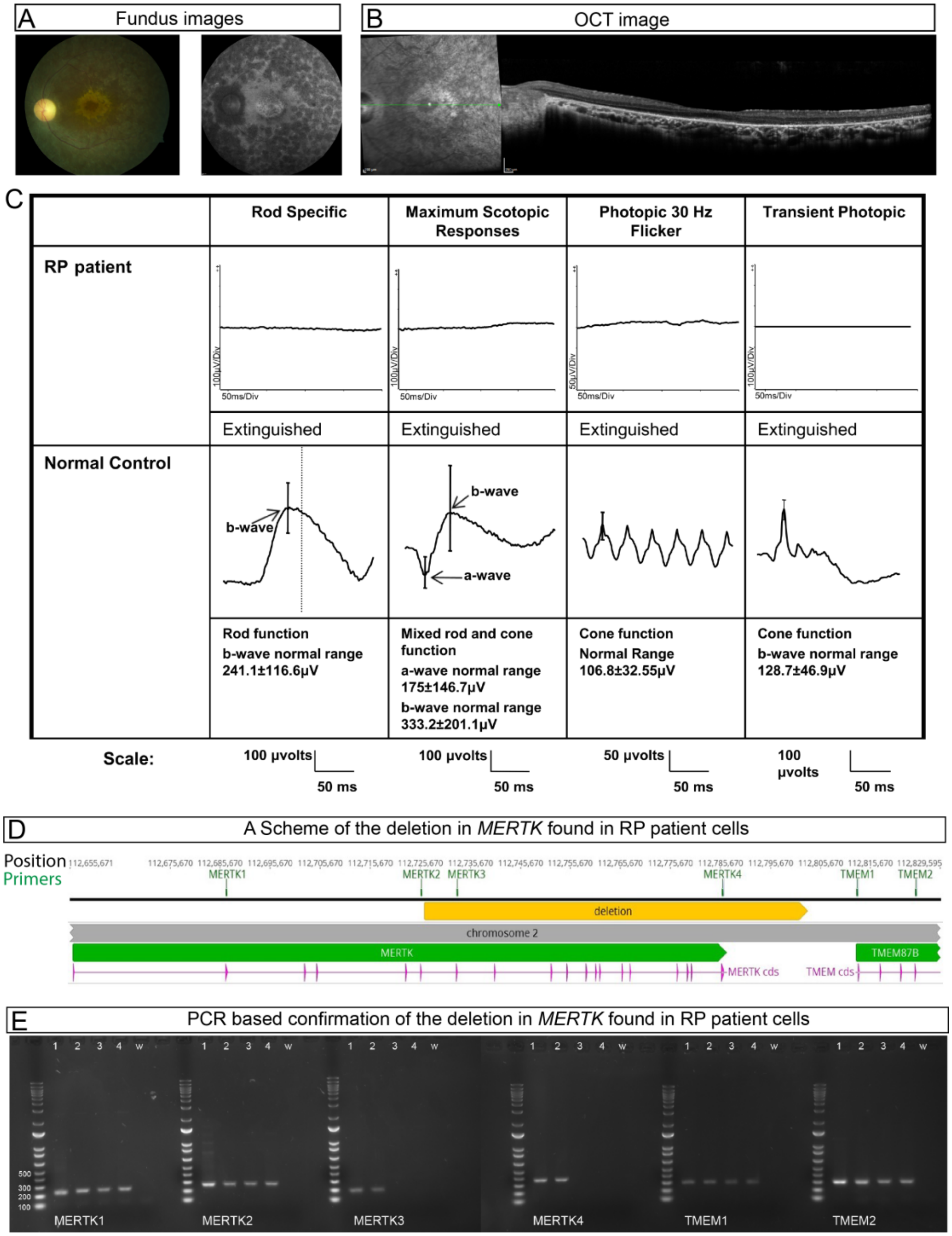

Figure S5. RP38 patient characterization. Related to Figure 5. (A) Fundus images of RP38 patient showed the migration of pigmented cells to the periphery and macular degeneration. (B) OCT image of RP38 patient showed the macular degeneration and thinning of the retina. (C) Full-field ERG was performed using Burian-Allen contact electrodes and with ganzfeld stimulation. All recordings showed very low amplitudes in the patient compared to the normal control. (D) Illustration of the deletion in *MERTK* found in the cells obtained from RP38 patient and the annealing location of the primer pairs used to confirm the mutation: Mertk1-4, TMEM 1-2. (E) Amplification result of the different primer pairs on genomic DNA from (1) a reference genome, (2) hESC, (3) patient iPSC clone 1, (4) patient iPSC clone 2. Primer pairs Mertk 3 and 4 showed no bands in the patient cell lines and a band in the wild-type DNA confirming the deletion.

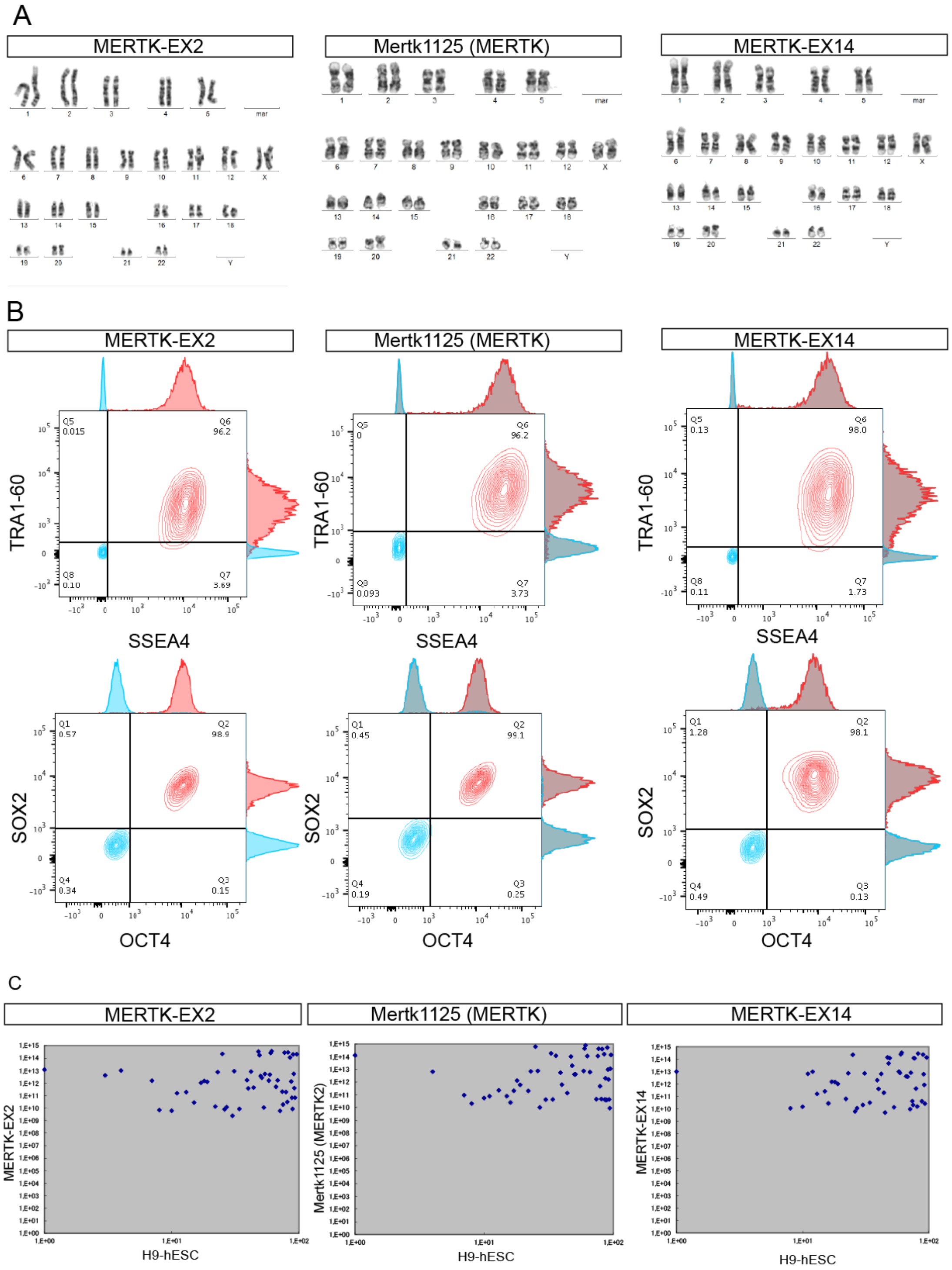

Figure S6. Characterization of iPSC and *MERTK* knock out cell lines. Related to Figure 5. (A) MERTK (clone Mertk1125); MERTK-EX2 and MERTK-EX14 had normal Karyotype. (B) FACS analysis showed that all stem cell lines expressed pluripotency markers OCT4, SOX2, TRA, and SSEA4. (C) Q-PCR analysis of pluripotency markers compared to H9 hESC using PrimerArray® Embryonic Stem Cells (Human) kit.

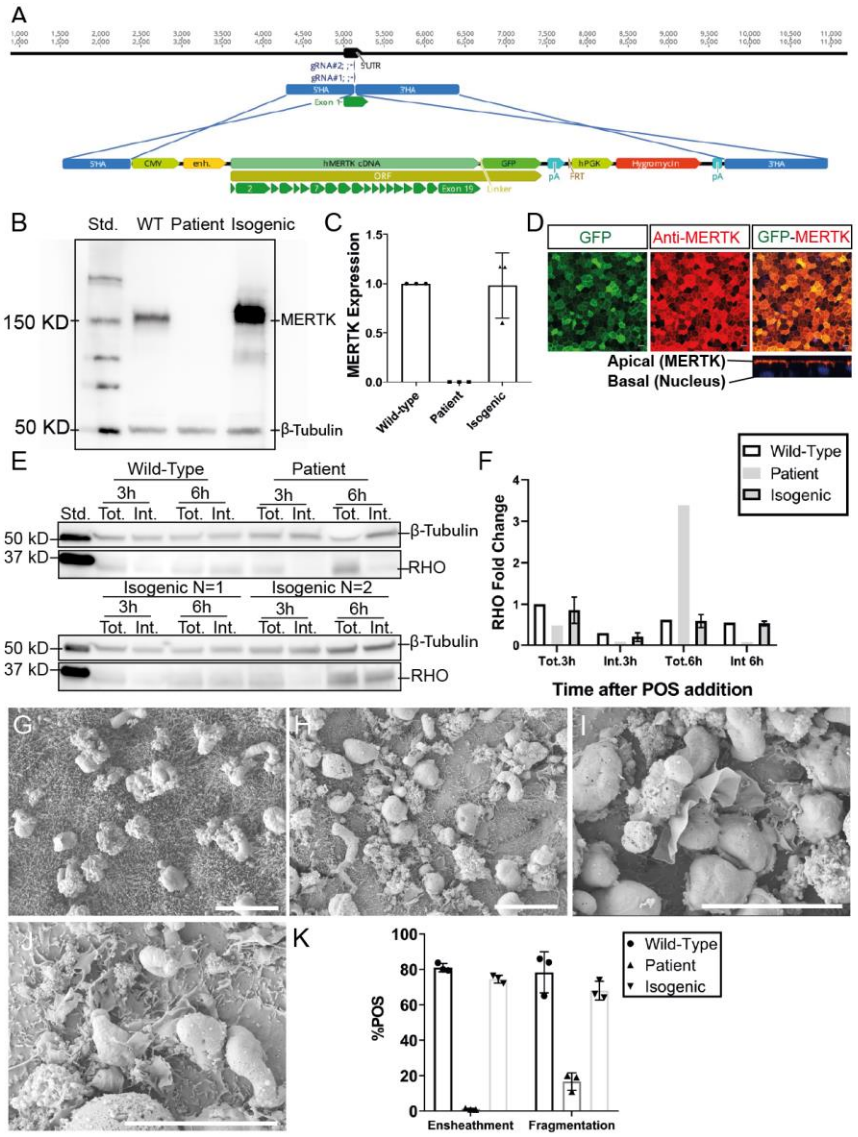

Figure S7. Rescue of MERTK expression in patient RPE restored POS phagocytosis and ensheathment. Related to Figure 5 and 6. (A) Genomic modification scheme in patient iPSC to recover MERTK expression. Isogenic control was created by integrating human MERTK cDNA under the CMV promoter in the MERTK locus using CRISPR/Cas9 system (see supplementary methods **Generation of the isogenic control**). 3' and 5'HA: 3' and 5' homology arms. gRNA: guide RNA. ORF: open reading frame. pA: polyadenylation signal. FRT: Flippase recognition target. hPGK: human Phosphoglycerate kinase promoter. (B) Immuno-blot analysis of MERTK expression in wild-type (WT) RPE (hESC-RPE), patient RPE (MERTK-RPE), and isogenic control RPE (TSS-RPE). MERTK is not expressed in patient RPE, while it is over expressed in the isogenic control. (C) Quantification of MERTK expression in A. Data are represented as the mean ± SD. N=3 Biological repeats. (D) Confocal microscopy fluorescence images of isogenic RPE showing MERTK-GFP (green) expression and Immuno-labeled anti MERTK (red). Overlay of MERTK-GFP and anti MERTK images is thus orange. Orthogonal view shows that MERTK-GFP is correctly localized to the apical membrane of the RPE. Nuclei are shown in blue. (E) Immuno-blot analysis of F-POS phagocytosis in wild-type RPE, patient RPE, and isogenic control RPE. Patient RPE fail to internalize POS after six hours, while isogenic RPE have internalization levels similar to wild-type RPE. (F) Quantification of immuno-blot in (D). Data of the isogenic control are represented as the mean ± SD. N=2 biological repeats. (G-J) SEM images. Scale bar: 10 µm. (G) Patient RPE shows no ensheathment or ensheathment mediated fragmentation of W-POS. (H) Isogenic RPE shows rescue of W-POS ensheathment and fragmentation defect in patient RPE. (I) W-POS ensheathment in isogenic RPE. (J) Ensheathment mediated fragmentation of W-POS in isogenic RPE. (K) Quantification of SEM images. N=3 biological replicates.

Supplemental data items: Excel table Legend

Tables S1. RNA sequencing data presented in **Figure S6D**. RPKM values are shown. Log-FC (fold change) shows the fold change in gene expression in RPE compared to hESC.

Supplemental data items: Videos Legends

Video S1a. Related to Figure 7a-b. Time-lapse of hESC-RPE and EX2-RPE cultured in transwells and treated with whole POS and 30% serum. Time-lapse was performed with an upright confocal microscope, LSM880, using the Airy scan function. ACTIN was labeled with SIR-Actin (magenta). POS were labeled with AF488 (green). Images were taken every 4 minutes. Of note is how the majority of POS were fragmented at the start of imaging (0 minutes), which was 3 hours after POS addition, while POS in EX2-RPE remained largely intact. When zooming in on POS, which were still not fragmented at the start of imaging (0 minutes), we observed fragmentation within 52 minutes in hESC-RPE, but not EX2-RPE. Scale bar: 10 µm.

Video S1b. Related to Figure 7a-b. Time-lapse of hESC-RPE cultured in transwells and treated with whole POS and 5µg/ml MFGE8. Time-lapse was performed with an upright confocal microscope LSM880 using the Airy scan function. ACTIN was labeled with SIR-Actin (magenta). POS were labeled with AF488 (green). Images were taken every 4 minutes. Of note is how most of the POS were not fragmented at the start of imaging (0 minutes), which was 3 hours after POS addition, nor at the end of imaging (92 minutes). Scale bar: 10 µm.

Video S2. Related to Figure 7 C-F. Time-lapse of hESC-RPE cultured in 96 well plates and treated with whole POS and 30% serum. Time-lapse was performed with an inverted confocal microscope, Leica Sp5MP. In one well, ACTIN was labeled with SIR-Actin (red), and in another well cells were labeled with Lysotracker (red) to monitor POS co-localization with lysosomes. POS were labeled with AF488 (green). Images were taken every 5 minutes. The same time-lapse is shown from different views to appreciate POS binding, fragmentation, internalization and co-localization with lysosomes.

Video S3. Related to Figure 7 C and F. Time-lapse of EX2-RPE cultured in 96 well plates and treated with whole POS and 30% serum. Time-lapse was performed with an inverted confocal microscope, Leica Sp5MP. **C**ells were labeled with Lysotracker (red) to monitor POS co-localization with lysosomes. POS were labeled with AF488 (green). Images were taken every 5 minutes. The same time-lapse is shown from different views. POS fragmentation, internalization and co-localization with lysosomes could not be observed.

Supplemental Experimental Procedures:

Key resources table		
**Reagent or Resource**	**SOURCE**	**IDENTIFIER**
**Antibodies**		
Alexa Fluor 488 anti-Oct3/4	Pharmingen	560253 BD
PE anti-Sox2	Pharmingen	560291 BD
V450-SSEA-4	Horizon	561156 BD
Alexa Fluor 647 anti-Tra-1-60	Pharmingen	560850 BD
Alexa Fluor® 488 Mouse IgG1 κ Isotype Control	Phosflow	557782 BD
PE Mouse IgG2a, κ Isotype Control	Pharmingen	555574 BD
V450 Mouse IgA, κ Isotype Control	Horizon	562142 BD
Alexa Fluor® 647 Mouse IgM, κ Isotype Control	Pharmingen	560806 BD
rhodopsin (Immunofluorescence)	Sigma	O4886
rhodopsin (Immuno-blot)	Abcam	ab5417
ZO1	Invitrogen	402200,
Bestrophin1	Abcam	ab2182
MITF	Abcam	ab122982
MERTK	Abcam	ab52968
EZRIN	Santa Cruz	sc-58758
β-TUBULIN	Thermo Fisher	PA5-16863
Alexa Fluor 488 rabbit	Invitrogen	A21206
Alexa Fluor 488 mouse	Invitrogen	A21202
Alexa Fluor 647 rabbit	Invitrogen	A31573
Alexa Fluor 647 mouse	Invitrogen	A31571
HRP-rabbit secondary antibody	Invitrogen	32260
HRP- mouse secondary antibody	Thermo Fisher	62-6520
rabbit anti mouse bridging antibody	Sigma	M7023_2ML
protein A gold	CMC Utrecht	https://www.cellbiology-utrecht.nl/products.html
**Plasmids**		
MERTK plasmid	Sino Biologicals Inc	HG10298-ACG
**Biological Samples**		
RP38 patient fibroblasts	This study	N/A
**Chemicals, Peptides, and Recombinant Proteins**		
soybean trypsin inhibitor	Sigma	T6522
trypsin-EDTA	Gibco	T3924
ACTIVIN A	R&D	338-AC-050
Antibiotic-Antimycotic	Gibco	15240-062
Cas9 NLS, *S. pyogenes* protein	New England Biolabs	M0646
TrypLE	Thermo Fisher	12563011
protease inhibitors	Roche	04693132001
Fluorescein	Invitrogen	F2182
Alexa Fluor 488	Invitrogen	A20000
Alexa Fluor 555	Invitrogen	A20009
Alexa Fluor 647 Phalloidin	Invitrogen	A22287

GAS6	R&D systems	885-GSB
MFGE8	R&D systems	2767-MF
PROS1	R&D systems	948-PS-100
VTN	R&D systems	2308-VN-050
Serum (FBS)	gibco	26140-079
trypan blue	Sigma	T8154
Paraformaldehyde	Science Services	E15714-S
Glutaraldehyde	Science Services	E16220
Hoechst 33342	Thermo Fisher	H1399
RIPA	Thermo Fisher	89900
BSA	SERVA	11926.03
10% Mini-PROTEAN® TGX™ Precast Protein Gels	BioRad	4561033
Precision Plus Protein™ WesternC™	BioRad	1610376
SuperSignal™ West	Thermo Fisher	34075
Precision Protein™ StrepTactin-HRP Conjugate	BioRad	1610380
Lowicryl K4M resin	Science Services	14330
osmium tetroxide	Science Services	19190
Protonex beads	ATT Bioquest	21209
mTESR1	StemCell Technologies	85870
SIR-Actin Kit	Cytoskeleton, Inc	CY-SC001
LysoTracker™ Deep Red	Invitrogen	L12492
Dispase	StemCell Technologies	07923
Rock inhibitor Y-27632	StemCell Technologies	78003
Hygromycin B	ThermoFisher Scientific	10687010
**Critical Commercial Assays**		
CytoTune TM-iPS 2.0 Sendai Reprogramming Kit	Thermo Fisher	A16518
human ES cell Primer Array	Takara Clontech	PH016
EnGen sgRNA Synthesis kit, S. pyogenes	New England Biolabs	E3322
P3 Primary Cell 4D-Nucleofector kit	LONZA	
QIAamp DNA Mini kit	Qiagen	51304
NEB PCR Cloning kit	New England Biolabs	E1202
RNeasy Mini kit	Qiagen	74106
NEBNext Poly(A) mRNA Magnetic Isolation Module	NEB	E7490L
NEBNext Ultra Directional RNA Library Prep Kit for Illumina	NEB	E7420L
XP bead purification	Beckman Coulter	A63882
Qubit dsDNA HS Assay Kit	Invitrogen	Q32854
NEBuilder® HiFi DNA Assembly Cloning Kit	NEB	E5520S
EnGen® sgRNA Synthesis Kit	NEB	E3322S
EnGen® Spy Cas9 NLS, NEB, M0646M		
Primary Cell 4D-Nucleofector Kit L (V4XP-3024, Lonza)		
**Deposited Data**		
RNA seq Raw and analyzed data	This paper www.ncbi.nlm.nih.gov/geo/	GSE127352
**Experimental Models: Cell Lines**		
H9 human embryonic stem cell line	WiCell	WA-09
MERTK knockout in H9 (MERTK-EX2)	This study	N/A
MERTK knockout in H9 (MERTK-EX14)	This study	N/A

RP38 patient iPSC (MERTK)	This study	N/A
TSS isogenic iPSC control	This study	N/A
**Software and Algorithms**		
CRISPR Design Tool	Hsu et al., 2013	crispr.mit.edu
CCTop	Stemmer et al., 2015	https://crispr.cos.uni-heidelberg.de/
Geneious software	Kearse *et al.*, 2012	https://www.geneious.com/
GraphPad Prism	La Jolla California USA,	www.graphpad.com
Knime	Berthold et al., 2009	
Cell Profiler	Lamprecht et al., 2007	
GSNAP (v2014-12-17)		http://research-pub.gene.com/gmap.
featureCounts (v1.4.6)	Liao et al., 2014	http://subread.sourceforge.net/
R		https://www.r-project.org/
DESeq2 R package (v1.6.3)	Love et al., 2014	https://bioconductor.org/packages/release/bioc/html/DESeq2.html
**Other**		
384 well plates	Greiner	781091
Transwells	Corning	3470
Nalgene centrifuge tubes	Thermo Fisher	3137-0050

Passaging of RPE cells

For passaging, RPE on transwells were incubated with trypsin-EDTA (TE) for ten minutes at 37ºC and 5% CO2. After incubation, cells were vigorously pipetted to obtain single cells, and transferred to a tube containing RPE medium plus soybean trypsin inhibitor. Next, RPE medium containing TE was removed by centrifugation at 180 g for two minutes. Finally, cells were resuspended with RPE medium containing 10-20 ng/ml ACTIVIN A and 1x Antibiotic-Antimycotic, and 45,000 cells per one 384 well, 200,000 cells per one 96 well plate or 300,000 cells per transwell were plated.

Immunofluorescence labeling of RPE cells

Cells were fixed with 4% paraformaldehyde (PFA) in 0.1 M phosphate buffer pH 7.4 (PFA-PB). After several washes with PBS, they were treated with a quenching solution (1xPBS, 100 mM Glycine, 0.3% Triton X-100) for twenty minutes and then were incubated with a blocking solution (1xPBS, 1% BSA, 0.3% Triton X-100) for one hour. The primary and secondary antibodies were diluted in the blocking solution and incubated overnight at 4°C. Cell nuclei were counterstained by addition of Hoechst 33342 (1mg/ml) to the secondary antibody solution. The primary antibodies and their working dilutions were: EZRIN (1:100), ZO1 (1:200), BEST1 (1:500), MITF (1:500), MERTK (1:1000). The secondary antibodies Alexa Fluor 488/647 rabbit and mouse were used at 1:500 dilution. Phalloidin 647 was added to the cells after the secondary antibody (1:1000).

Confocal imaging

RPE cells grown in transwells were cut out from the well, transferred on slides and imaged using SP5-MP confocal microscope. Alternatively, CV7000S confocal microscope was used for imaging RPE cells in 384 well plates.

POS isolation

POS were isolated from porcine eyes, as described in (Molday, Hicks and Molday, 1987) with some modifications **(Figure S1 A)**. Briefly, retinal tissue was isolated from 50 porcine eyes and homogenized with a Dounce glass tissue homogenizer in a buffer containing protease inhibitors, 0.2 mM Tris-HCl, 0.1 mM Glucose, 130 mM $NaCl_2$, 0.1 mM Taurine, and 0.02 mM $MgCl_2$. The homogenized retinal tissue was split in 6 tubes containing a sucrose

gradient of 27%, 33%, 41%, 50% and 60%, and centrifuged in the Beckmann Coulter ultracentrifuge B409 at 28000 rpm for one hour at 4ºC. The orange band was collected in Nalgene centrifuge tubes, and centrifuged at 13000 g for ten minutes. Finally, the pellets were resuspended with POS storage solution containing protease inhibitors, 10mM phosphate buffer pH 7.2, 100 mM $NaCl_2$ and 2.5% sucrose and stored at -80ºC until they were used. POS obtained using this method are around 0.5-1 µM in size and are referred to as fragmented POS (F-POS) throughout the manuscript. To obtain whole length POS particles, isolated porcine retinas were shaken thoroughly in homogenization buffer containing 20% sucrose, 20mM Tris Acetate, 2mM MgCl2, 10 mM glucose, and 5 mM Taurine. Then retinal tissue was filtered 3 times through double layers of gauze as originally described (Molday, Hicks and Molday, 1987). The filtered retinal tissue was split in 6 tubes containing a sucrose gradient of 27%, 33%, 41%, 50% and 60%, and centrifuged in the Beckmann Coulter ultracentrifuge B409 at 28000 rpm for one hour at 4ºC. The faint orange band was collected from each gradient, and diluted 5x with wash buffer containing, 10% sucrose, 20mM sodium phosphate buffer and 5 mM Taurine, and centrifuged at 4800 rpm for ten minutes. Finally, the pellets were resuspended with POS storage solution containing DMEM with 2.5% sucrose and stored at -80ºC until they were used as described in (Parinot *et al.*, 2014). POS obtained using this method are 5-10 µM in size and are referred to as whole POS (W-POS) throughout the manuscript.

POS labeling

For labeling, Fluorescein (FITC) or Alexa Fluor 488 or 555 (AF488, AF555) were added to the POS after thawing for one hour at 25°C with shaking (500 rpm). Next, F-POS were centrifuged at 9000 rcf at 4°C for 10 minutes, while W-POS were centrifuged at 3600 rcf for 5 minutes. Pellets were washed twice with the washing buffer, containing; 10% sucrose, 20 mM phosphate buffer pH7.2 and 5 mM Taurine.

POS phagocytosis assay variations

To distinguish total and internalized F-POS by means of fluorescence imaging, 0.4% trypan blue was added to half of the wells for 10 minutes and then washed six times with PBS to remove leftover dye before methanol fixation for 5 minutes, as described previously (Mao and Finnemann, 2013). Six images from each replicate were acquired covering the different areas in the well. Acquired images were imported into the Cell Profiler software for image analysis. POS and nuclei were segmented and values of the number, size and intensity were obtained. The data obtained from image analysis was imported into KNIME for statistical analysis. GraphPad Prism was used for statistical significance calculations and final graph presentation. The median count of the POS between the 6 images was divided by the median count of cells per well to obtain the POS/cell count.

To distinguish total and internalized F-POS by means of immuno-blot, half of the samples were treated with 2 mM EDTA for ten minutes at 37 ºC to remove bound POS from the cells before lysis and the other half was lysed without treatment as described previously (Mao and Finnemann, 2013).

Immuno-blots

Around one million cells were lysed with RIPA buffer for 30 minutes at 4°C. Next, the protein lysates were separated from the pellet by centrifugation and around 50 µg of protein was loaded into Mini-PROTEAN® TGX™ Precast Protein Gels. 10 µl of Precision Plus Protein™ WesternC™ was loaded as a standard. Proteins were transferred into PVDF membrane using TE70 Semi-dry transfer unit. Blocking was done for one hour at room temperature in 5% milk powder dissolved in TBST. To look at MERTK expression in the different wild-type and *MERTK* mutant RPE, primary antibody incubation was done with MERTK (1:1000) antibody recognizing the N-terminal of the protein and β-TUBULIN (1:1000) antibody overnight at 4°C in 5% milk-TBST. HRP-rabbit secondary antibody (1:1000) was added for one hour at room temperature in 5% milk-TBST. To analyze POS phagocytosis by means of Immuno-blot membranes were incubated with anti-RHO antibody (1:1000), which recognizes the C-terminal of the protein, followed by HRP-mouse secondary antibody (1:1000). Precision Protein™ StrepTactin-HRP conjugate was also added with the secondary to view the standard in chemiluminescence mode. The blot membrane was incubated with SuperSignal™ West according to Manufacturer´s instructions and imaged with LAS4000.

Live imaging of POS phagocytosis

Two confocal systems were used for POS phagocytosis live imaging. The first system was LSM 880 upright using the Airyscan detector. Dipping objective with 40X magnification was used. Time lapse images were processed with Airyscan Processing method in the ZEN-black software. HESC-RPE and EX2-RPE (grown on transwells) were primed with 30% serum or 5µg/ml MFGE8 for 1 hour, and then they were challenged with AF488 labeled W-POS and left for two-and-a half hours at 37°C with 5% CO2 before imaging. Membranes, containing the RPE monolayer, were cut out from the transwell insert and placed with the apical side of the cells facing the dipping objective in a 35

mm cell culture dish with 2 ml media containing SiR-Actin dye and 30% serum. Images were taken every four minutes. Alternatively, live fluorescence imaging was performed with an inverted Leica confocal microscope (SP5-mp) on hESC-RPE and EX2-RPE plated in 96 well plates, and treated with 30% serum and SiR-Actin or Lysotracker, to monitor W-POS internalization or co-localization with lysosomes respectively. Following priming with 30% serum POS were added to the cells and imaging started without a washing step in between. In both systems, sample preparation and setting up imaging parameters usually took around 30 minutes. Image analysis was done with the Cell Profiler software (Lamprecht, Sabatini and Carpenter, 2007) and data analysis and figure preparation was done with KNIME (Berthold *et al.*, 2009).

Transmission Electron Microscopy (TEM)

POS pellets were either fixed in modified Karnovsky's solution and embedded in Epon as previously described (Zhu *et al.*, 2013), or fixed in 4% PFA-PB for immuno-EM.

For immuno-EM, the samples were fixed in 4% PFA in 0.1 M phosphate buffer pH 7.4. After several washes in PBS, samples were dehydrated and infiltrated in Lowicryl K4M resin using the progressive lowering of temperature (PLT) method (Carlemalm, Garavito and Villiger, 1982). After polymerisation, the blocks were raised up to room temperature, and 70 nm sections were cut on a Leica UC6 ultramicrotome. Sections were mounted onto copper mesh grids for immunolabeling. On-section labeling of ultrathin sections was performed as previously described (Fabig *et al.*, 2012). In brief, sections were blocked with 1% BSA in PBS, incubated for one hour with primary antibodies mouse anti-RHO (1:200) in BSA/PBS. Next, they were washed with PBS, and incubated with bridging antibody (rabbit anti mouse, 1:100 in BSA/PBS) for thirty minutes. Sections were then washed with PBS, incubated with protein A 10 nm gold (1:100) for one hour in BSA/PBS, washed with PBS, post-fixed in 1% glutaraldehyde/PBS, washed with water, stained with 4% uranyl acetate in water, washed with water and dried for TEM inspection. Ultrastructure and gold labeling was imaged with a FEI Morgagni 268D or a Jeol JEM1400 Plus both at 80 kV acceleration voltage.

SEM sample processing:

SEM samples were fixed in modified Karnovsky (2% glutaraldehyde, 2% formaldehyde in 50 mM HEPES), washed with PBS, and post-fixed in 1% osmium tetroxide in PBS for two hours on ice. Next, they were washed with water and dehydrated in a graded series of ethanol/water starting from 30% and up to 100% ethanol on molecular sieve. Then, they were critical-point dried using the Leica CPD 300 (Leica Microsystems, Vienna, Austria), cut out from the transwell, mounted on 12 mm aluminium stubs, and sputter-coated with gold using the Baltec SCD 050 (Leica Microsystems, Vienna, Austria). Finally, filters were analyzed with a Jeol JSM 7500F cold field emission SEM (Jeol, Eching, Germany) at 5 kV acceleration voltage using the lower secondary electron detector. To correlate fluorescence images of rhodopsin positive POS and SEM images, POS were labeled with AF555 and added to hESC-RPE. After three hours the cells were fixed with 4% PFA, and labeled with anti-ZO1 antibody and anti-RHO antibodies followed by anti-mouse AF488. Then, they were post fixed with 4% PFA for twenty minutes, washed in PBS, dehydrated and critical-point dried. Samples were mounted on stubs and imaged first with an upright confocal fluorescence microscope (SP5-I), and then they were sputter-coated with gold and imaged with the SEM.

Generation and characterization of *MERTK* mutant hPSC lines

Reprogramming of patient fibroblasts and genomic engineering in iPSC was done in collaboration with the Stem Cell Engineering Facility in the CRTD. Two *MERTK* knockout hESC lines; namely EX2 (homozygous deletion from Exon 2 onwards), and EX14 (heterozygous deletion of Exon 14 onwards), were generated using CRISPR/Cas9 in hESCs. Nucleotides homozygous deletion in exon 2 was confirmed by allele specific sequencing **(Figure S4 A)**. The deletion resulted in a frameshift mutation and premature stop in both allele as shown in the predicted amino acid sequence **(Figure S4 B)**. In exon 14, two deletions were confirmed by allele specific sequencing **(Figure S4 C)** resulting in two amino acid sequence predictions for each allele. In the first allele (Allele 1) amino acids 646 – 652 (HPNVIRL) were deleted. In the second allele (Allele 2), nucleotides deletion resulted in a frameshift mutation and a premature stop **(Figure S4 D)**.

Guides were designed and finalized by using the Geneious software or the online guide prediction tools; including CRISPR Design Tool, and CCTop. In vitro transcribed guides were generated using the EnGen sgRNA Synthesis kit. We chose guides that were common and showed the least off-target, in the virtual tools. The sequences of the guides used to generate Exon 14 and Exon 2 deletion along with respective PAMs can be found in the table below.

Oligonucleotide sequence	SOURCE
Exon14 Guide 1: CTCTTCACAGCGGGAGATCG AGG	This Study
Exon14 Guide 2: GAAGTCGAATGACATTTGGG TGG	This Study
Exon2 Guide: TAGGGGCTTTGATTCGACAGAGG	This Study

S. pyogenes Cas9 NLS (5.5 µg) was mixed with 1 µg of each in vitro transcribed guide RNA, and incubated for twenty minutes at room temperature before they were electroprated into H9 hESC single cell suspension, using Lonza's 4D-Nucleofector and the P3 Primary Cell 4D-Nucleofector kit.

Colonies were picked and screened by Sanger sequencing (Yusa, 2013) of the *MERTK* Exon 2 or Exon 14 regions. Colonies showing indels at the guide sites were individually expanded. Genomic DNA was extracted from the indel positive clones with the QIAamp DNA Mini kit', and was used as a template to amplify the region flanking the Exon 14 of *MERTK* gene using F-primer1 and R-primer2.

Oligonucleotide sequence	SOURCE
F-primer1: 5' AGAAAGTACAGAGAATGCCCATAGGTCC 3'	This Study
R-primer2: 5' ACAGCATCCACTCCATACTTAATCTCC 3'	This Study

The resulting fragment (~ 1.2 kb) was cloned using the NEB PCR Cloning kit and sequenced to determine allele specific sequences. Similarly, F-primer3 and R-primer4 were used for amplification of genomic fragments (~0.5 kb) for cloning and sequencing Exon 2 deletion.

Oligonucleotide sequence	SOURCE
F-primer3: 5' TTCTTGGCCTAAGAAGTTGGGAACCTACTTGG 3'	This Study
R-primer4: 5' GAACACATACCTGAAGGAAGCGATTATTGCTG 3'	This Study

RPE was differentiated from MERTK-EX14 and MERTK-EX2 knockout hESC cell lines as previously described (Zhu *et al.*, 2013; Zhu, Schreiter and Tanaka, 2014), and is referred to as EX14-RPE and EX2-RPE in this study.

To validate the phenotype observed in *MERTK* knockout cell lines, an iPSC cell line was derived from a patient diagnosed with RP38, harboring a homozygous genomic deletion in *MERTK.* The patient (female) was diagnosed at 12 years of age with RP38. She had night blindness, and on dilated fundus exam, she had a bull´s eye pattern of atrophy on both maculae **(Figure S5 A-B)**. Peripheral fundus showed extensive mottling and intra-retinal pigment migration. The optic nerve was symmetric with a good rim but with temporal pallor. Optical coherence tomography (OCT) images showed thinning of the outer nuclear layer, and almost no RPE layer was left in the macula region. The patient was prescribed vitamin A supplements. However, peripheral vision deteriorated over time and the patient complained of photopsia (constant light flashes). Electroretinogram (ERG) responses were extinguished **(Figure S5 C)**. Genetic analysis of patient derived fibroblasts, using the Affymetrix Cytoscan HD array, showed that the patient had a homozygous loss in the DNA region 2q13 corresponding with the *MERTK* gene with the coordinates 112,726,509-112,803,074 based on the GRCh37 assembly. The 77 bp deletion of hMERTK (exons 7-19) is illustrated in **Figure S5 D**. The deletion was confirmed in both fibroblasts and fibroblast-derived iPSC by means of PCR **(Figure S5 E).** The primers used for PCR based confirmation are listed in the table below:

Primer	Position in Chr 2	Sequence	Amplicon Size (bp)
MERTK-1-fwd	112,686,816<br>112,686,835	CCTGCCTTGATGTTTTCACC	228
MERTK-1-rev	112,687,043<br>112,687,024	TTCCTTCCCATCTTTCCACC	
MERTK-2-fwd	112,725,762<br>112,725,781	ACAGCATTCTGATCTCCTGG	256
MERTK-2-rev	112,726,017<br>112,725,998	ACACAGTATCATGGGCAGAG	
MERTK-3-fwd	112,732,993<br>112,733,012	ATGAAATAGGCTGGTCTGCA	176

MERTK-3-rev	112,733,168<br>112,733,149	CTCCTCCAGAGACAAAGTCC	
MERTK-4-fwd	112,785,993<br>112,786,012	GGCTGCAGCTAGAAAAACTC	355
MERTK-4-rev	112,786,347<br>112,786,328	CAGGAGACCCCATTCCTAAC	
TMEM-1-fwd	112,812,901<br>112,812,920	CACATCCTGGCTCCTATCTC	275
TMEM-1-rev	112,813,175<br>112,813,156	CGACCATCTTGACCAGGAA G	
TMEM-2-fwd	112,824,616<br>112,824,635	GTGATTCACAGGTGTTTCCC	294
TMEM-2-rev	112,824,909<br>112,824,890	CCCAGCCACGTATCTTACTT	

Patient iPSC and *MERTK* hESC knockout cell lines showed a normal karyotype **(Figure S6 A)**. They also expressed high levels of pluripotency markers as determined by flow cytometry **(Figure S6 B)**. The following antibodies were used: Alexa Fluor 488 anti-Oct3/4, PE anti-Sox2, V450-SSEA-4, and Alexa Fluor 647 anti Tra-1-60. Isotype antibodies (resources table) and unstained cells were used for gating. Antibodies dilutions that were recommended by the manufacturer were used. The antibodies with the recommended dilutions did not label fibroblasts, which are negative for these markers. Pluripotency of the three hPSC lines was also confirmed by qRT-PCR according to the International Stem Cell Initiative International (ISCI) using the human ES cell Primer Array **(Figure S6 C)**.

Generation of the isogenic control:
MERTK expression in patient RPE was rescued by inserting hMERTK as cDNA near the **t**ranscription **s**tart **s**ite (TSS) into exon 1 of one of the endogenous alleles using CRISPR/Cas9-assisted gene targeting. The TSS targeting vector was generated from a custom synthesized plasmid backbone from ThermoFisher Scientific containing the ColE1 origin of replication and Ampicillin resistance. The insert from this plasmid was removed by HindIII and SfiI based restriction digestion and replaced with 4 PCR fragments using the NEBuilder® HiFi DNA Assembly Cloning Kit. The regions flanking the targeting site at the 5’ end (851 bp) and the 3’ end (1283 bp) were amplified from RP38 patient iPSC clone hMERTK1125#4 genomic DNA to serve as 5’ and 3’ homology arms **(Figure S7 A)**. The insert consisting of the CMV promoter followed by an ORF containing hMERTK cDNA with GFP-tag and polyadenylation signal was PCR-amplified from a MERTK plasmid, followed by a custom synthesized FRT-flanked PGK-hygro selection cassette from ThermoFisher Scientific.
SgRNAs targeting the region close to the TSS was *in vitro*-transcribed using EnGen® sgRNA Synthesis Kit. The sgRNA target sites for TSS were excluded from the targeting vector to protect it from CRISPR/Cas9-induced double-stranded breaks.
Patient iPSC clone MERTK1125#4 ($8*10^5$ cells) were electroporated with 10 µg linearized targeting vectors, 2 µg sgRNA and 19 µg Cas9-NLS protein (EnGen® Spy Cas9 NLS) with the Lonza 4D X-unit, pulse CB-150 and the Primary Cell 4D-Nucleofector Kit L and seeded at clonal density on Matrigel-coated dishes in mTeSR1 medium supplemented for 4 days with 10 µM Rock inhibitor Y-27632. Cells were selected with 40 µg/ml Hygromycin B starting on day three after nucleofection and up to six days. Resistant colonies were manually picked, clonally expanded and screened for proper targeting by colony PCR amplifying the 5’ and 3’ junction of the targeted alleles from outside of the homology arms into the insert. The presence of an intact second allele was also verified. Heterozygous clones were selected and the complete insert was analysed by Sanger sequencing. Clone hMERTK1125-4-TSS #10 showed an intact 46,XX[cp20] chromosome set like the parental line and was used in this manuscript.

**Oligonucleotide sequence**	**SOURCE**
hMERTK-TSS Guide 1: 5’-ACTGCCCGGGCCGCCCGGAC-3’	This Study
hMERTK-TSS Guide 2: 5’-CCCGGGCAGTGAGTGCCGAG-3’	This Study

Next-generation sequencing (RNA-seq)

hESC-RPE was differentiated on transwells as described previously (Zhu *et al.*, 2013; Zhu, Schreiter and Tanaka, 2014) and total RNA was purified from three consecutive RPE and hESC preps, using the Qiagen RNeasy Mini kit. Using the NEBNext Poly(A) mRNA Magnetic Isolation Module, polyadenylated mRNA was enriched from 1 µg total RNA with an integrity number of ≥ 9, according to the manufacturer's instructions. The mRNA was eluted in 15 µl 2x first strand cDNA synthesis buffer (NEBnext), in order to chemically fragment the samples, followed by reverse transcription, second strand synthesis, end repair, A tailing and adapter ligation according to the manual of NEBNext Ultra Directional RNA Library Prep Kit for Illumina. For ligation, hybridized custom adaptors were used (Adaptor-Oligo 1 and 2). Afterwards, excess of non-ligated adapters were depleted by XP beads purification, adding beads in a ratio of 1:1. Finally, samples were indexed during a PCR enrichment step with 15 cycles of amplification using primers carrying a specific index sequence indicated with 'NNNNNN' (Index Primer1, 2 and 3). After two more rounds of XP beads purifications (1:1), libraries were quantified using the Qubit dsDNA HS Assay Kit. For Illumina flowcell production, samples were equimolarly pooled and distributed over all lanes on the Illumina HiSeq 2500 to sequence 75bp single reads.

**Oligonucleotide sequence**	**SOURCE**
Adaptor-Oligo 1: 5'-ACA CTC TTT CCC TAC ACG ACG CTC TTC CGA TCT 3'	This Study
Adaptor-Oligo 2: 5'-P-GAT CGG AAG AGC ACA CGT CTG AAC TCC AGT CAC-3'	This Study
Index Primer1: AAT GAT ACG GCG ACC ACC GAG ATC TAC ACT CTT TCC CTA CAC GAC GCT CTT CCG ATC T	This Study
Index Primer2: GTG ACT GGA GTT CAG ACG TGT GCT CTT CCG ATC T	This Study
Index Primer3: CAA GCA GAA GAC GGC ATA CGA GAT NNNNNN GTG ACT GGA GTT)	This Study

Next-generation sequencing (RNA-seq) bioinformatics

Resulting reads were mapped with GSNAP (v2014-12-17) to the human genome (hg38) using splice junction information from Ensembl (v81) as support. Uniquely mapped reads were then converted into counts per gene using featureCounts (v1.4.6) and gene annotations from Ensembl (v81). Normalization of the raw counts based on the library size and testing for differential expression between the different cell types/treatments was performed with the DESeq2 R package (v1.6.3). Genes with adjusted p-value (Benjamini-Hochberg) less than 0.05 were considered differentially expressed. RPKM data were generated in R. The heat map presented in supplementary figure S6 was generated using GraphPad Prism. LOG2 of the mean of the RPKM values of the three biological repeats for the genes of interest, was plotted in the heat map shown in **Figure 1**. Individual RPKM values are shown in excel **Table S1.** The raw and analyzed data are deposited in GEO (www.ncbi.nlm.nih.gov/geo/) with the accession number: GSE127352.

Supplemental References:

Berthold, M. R. *et al.* (2009) 'KNIME - the Konstanz information miner', *ACM SIGKDD Explorations Newsletter*, 11(1), p. 26. doi: 10.1145/1656274.1656280.

Carlemalm, E., Garavito, R. M. and Villiger, W. (1982) 'Resin development for electron microscopy and an analysis of embedding at low temperature', *Journal of Microscopy*, 126(2), pp. 123–143. doi: 10.1111/j.1365-2818.1982.tb00362.x.

Fabig, G. *et al.* (2012) 'Labeling of Ultrathin Resin Sections for Correlative Light and Electron Microscopy', *Methods in Cell Biology*, 111, pp. 75–93. doi: 10.1016/B978-0-12-416026-2.00005-4.

Hsu, P. D. *et al.* (2013) 'DNA targeting specificity of RNA-guided Cas9 nucleases', *Nature Biotechnology*. Nature Publishing Group, 31(9), pp. 827–832. doi: 10.1038/nbt.2647.

Lamprecht, M. R., Sabatini, D. M. and Carpenter, A. E. (2007) 'CellProfiler: Free, versatile software for automated biological image analysis', *BioTechniques*, 42(1), pp. 71–75. doi: 10.2144/000112257.

Liao, Y., Smyth, G. K. and Shi, W. (2014) 'featureCounts: an efficient general purpose program for assigning sequence reads to genomic features.', *Bioinformatics (Oxford, England)*, 30(7), pp. 923–30. doi: 10.1093/bioinformatics/btt656.

Love, M. I., Huber, W. and Anders, S. (2014) 'Moderated estimation of fold change and dispersion for RNA-seq data with DESeq2', *Genome Biology*. BioMed Central, 15(12), p. 550. doi: 10.1186/s13059-014-0550-8.

Mao, Y. and Finnemann, S. C. (2013) 'Analysis of Photoreceptor Outer Segment Phagocytosis by RPE Cells in Culture', *Methods in Molecular Biology*, 935, pp. 285–295. doi: 10.1007/978-1-62703-080-9.

Molday, R., Hicks, D. and Molday, L. (1987) ‘Peripherin . A Rim-Specific Membrane Protein of Rod Outer Segment Discs’, *Investigative ophthalmology & visual science*, 28, pp. 50–61.
Parinot, C. *et al.* (2014) ‘Large-scale purification of porcine or bovine photoreceptor outer segments for phagocytosis assays on retinal pigment epithelial cells’, *Journal of Visualized Experiments*, (94), pp. 1–8. doi: 10.3791/52100.
Stemmer, M. *et al.* (2015) ‘CCTop: An Intuitive, Flexible and Reliable CRISPR/Cas9 Target Prediction Tool’, *PLOS ONE*. Edited by S. Maas. Public Library of Science, 10(4), p. e0124633. doi: 10.1371/journal.pone.0124633.
Yusa, K. (2013) ‘Seamless genome editing in human pluripotent stem cells using custom endonuclease-based gene targeting and the piggyBac transposon’, *Nat Protoc*, 8(10), pp. 2061–2078. doi: 10.1038/nprot.2013.126.
Zhu, Y. *et al.* (2013) ‘Three-Dimensional Neuroepithelial Culture from Human Embryonic Stem Cells and Its Use for Quantitative Conversion to Retinal Pigment Epithelium’, *PLoS ONE*, 8(1), p. e54552. doi: 10.1371/journal.pone.0054552.
Zhu, Y., Schreiter, S. and Tanaka, E. M. (2014) ‘Accelerated Three-Dimensional Neuroepithelium Formation from Human Embryonic Stem Cells and Its Use for Quantitative Differentiation to Human Retinal Pigment Epithelium’, *Methods in Molecular Biology (Clifton, N.J.)*, 1307, pp. 345–355. doi: 10.1007/7651_2013_56.
